# The genomic landscape of rare disorders in the Middle East

**DOI:** 10.1186/s13073-023-01157-8

**Published:** 2023-01-27

**Authors:** Maha El Naofal, Sathishkumar Ramaswamy, Ali Alsarhan, Ahmed Nugud, Fatima Sarfraz, Hiba Janbaz, Alan Taylor, Ruchi Jain, Nour Halabi, Sawsan Yaslam, Roudha Alfalasi, Shruti Shenbagam, Fatma Rabea, Martin Bitzan, Lemis Yavuz, Deena Wafadari, Hamda Abulhoul, Shiva Shankar, Munira Al Maazmi, Ruba Rizk, Zeinab Alloub, Haitham Elbashir, Mohamed O. E. Babiker, Nidheesh Chencheri, Ammar AlBanna, Meshal Sultan, Mohamed El Bitar, Safeena Kherani, Nandu Thalange, Sattar Alshryda, Roberto Di Donato, Christos Tzivinikos, Ibrar Majid, Alexandra F. Freeman, Corina Gonzalez, Arif O. Khan, Hisham Hamdan, Walid Abuhammour, Mohamed AlAwadhi, Abdulla AlKhayat, Alawi Alsheikh-Ali, Ahmad N. Abou Tayoun

**Affiliations:** 1 Al Jalila Genomics Center of Excellence, Al Jalila Children’s Specialty Hospital, Dubai, United Arab Emirates; 2 General Pediatrics Department, Al Jalila Children’s Specialty Hospital, Dubai, United Arab Emirates; 3 College of Medicine, Mohammed Bin Rashid University of Medicine and Health Sciences, Dubai, United Arab Emirates; 4 Kidney Center of Excellence, Al Jalila Children’s Specialty Hospital, Dubai, United Arab Emirates; 5 Department of Metabolic Medicine, Al Jalila Children’s Specialty Hospital, Dubai, United Arab Emirates; 6 Critical Care Centre of Excellence, Al Jalila Children’s Specialty Hospital, Dubai, United Arab Emirates; 7 Adolescent Medicine, Al Jalila Children’s Specialty Hospital, Dubai, United Arab Emirates; 8 Neurodevelopment Section, Al Jalila Children’s Specialty Hospital, Dubai, United Arab Emirates; 9 Neuroscience Center of Excellence, Al Jalila Children’s Specialty Hospital, Dubai, United Arab Emirates; 10 Neurology Department, Al Jalila Children’s Specialty Hospital, Dubai, United Arab Emirates; 11 Mental Health Center of Excellence, Al Jalila Children’s Specialty Hospital, Dubai, United Arab Emirates; 12 ENT Department, Al Jalila Children’s Specialty Hospital, Dubai, United Arab Emirates; 13 Endocrinology Department, Al Jalila Children’s Specialty Hospital, Dubai, United Arab Emirates; 14 Orthopedics Department, Al Jalila Children’s Specialty Hospital, Dubai, United Arab Emirates; 15 Cardiology Department, Al Jalila Children’s Specialty Hospital, Dubai, United Arab Emirates; 16 Gastroenterology Department, Al Jalila Children’s Specialty Hospital, Dubai, United Arab Emirates; 17 National Institute of Allergy and Infectious Diseases, NIH, Bethesda, MD USA; 18 Immune Deficiency Cellular Therapy Program, National Cancer Institute, NIH, Bethesda, MD USA; 19 Eye Institute, Cleveland Clinic Abu Dhabi, Abu Dhabi, United Arab Emirates; 20 Pulmonology and Sleep Medicine Department, Al Jalila Children’s Specialty Hospital, Dubai, United Arab Emirates; 21 Infectious Diseases Department, Al Jalila Children’s Specialty Hospital, Dubai, United Arab Emirates; 22 Al Jalila Children’s Specialty Hospital, Dubai, United Arab Emirates; 23 Dubai Health Authority, Dubai, United Arab Emirates; 24 Center for Genomic Discovery, Mohammed Bin Rashid University of Medicine and Health Sciences, Dubai, United Arab Emirates

**Keywords:** Genomics, Rare diseases, Whole-exome sequencing, Middle East, Diagnostic yield, Clinical utility

## Abstract

**Background:**

Rare diseases collectively impose a significant burden on healthcare systems, especially in underserved regions, like the Middle East, which lack access to genomic diagnostic services and the associated personalized management plans.

**Methods:**

We established a clinical genomics and genetic counseling facility, within a multidisciplinary tertiary pediatric center, in the United Arab Emirates to locally diagnose and manage patients with rare diseases. Clinical genomic investigations included exome-based sequencing, chromosomal microarrays, and/or targeted testing. We assessed the diagnostic yield and implications for clinical management among this population. Variables were compared using the Fisher exact test. Tests were 2-tailed, and *P* < .05 was considered statistically significant.

**Results:**

We present data on 1000 patients with rare diseases (46.2% females; average age, 4.6 years) representing 47 countries primarily from the Arabian Peninsula, the Levant, Africa, and Asia. The cumulative diagnostic yield was 32.5% (95% CI, 29.7–35.5%) and was higher for genomic sequencing-based testing than chromosomal microarrays (37.9% versus 17.2%, *P* = 0.0001) across all indications, consistent with the higher burden of single gene disorders. Of the 221 Mendelian disorders identified in this cohort, the majority (*N* = 184) were encountered only once, and those with recessive inheritance accounted for ~ 62% of sequencing diagnoses. Of patients with positive genetic findings (*N* = 325), 67.7% were less than 5 years of age, and 60% were offered modified management and/or intervention plans. Interestingly, 24% of patients with positive genetic findings received delayed diagnoses (average age, 12.4 years; range 7–37 years), most likely due to a lack of access to genomic investigations in this region. One such genetic finding ended a 15-year-long diagnostic odyssey, leading to a life-threatening diagnosis in one patient, who was then successfully treated using an experimental allogenic bone marrow transplant. Finally, we present cases with candidate genes within regions of homozygosity, likely underlying novel recessive disorders.

**Conclusions:**

Early access to genomic diagnostics for patients with suspected rare disorders in the Middle East is likely to improve clinical outcomes while driving gene discovery in this genetically underrepresented population.

**Supplementary Information:**

The online version contains supplementary material available at 10.1186/s13073-023-01157-8.

## Background

Although individually rare, the cumulative prevalence of the roughly 7000 known rare diseases can be as high as 6%; nearly 450 million individuals may be affected globally [1, 2]. Despite the fact that most of these diseases have genetic origins, affected patients go through extended diagnostic odysseys averaging 6–8 years, characterized by multiple hospitalizations, unnecessary diagnostic workup, and/or inefficient management or treatment plans, leading to substantial social and economic burden on families and healthcare systems [3, 4].

The prevalent close relative marriages and large family structures in populations of the Middle East are expected to lead to high rates of rare Mendelian disorders [5]. On the other hand, the lack of specialized care centers and the limited access to genomic services [6] are likely to contribute to longer diagnostic odysseys than have been described elsewhere, resulting in missed opportunities for personalized management plans.

To address this gap, we established a dedicated clinical genomics center in one of the first standalone children’s specialty hospitals in the Middle East, Al Jalila Children’s Specialty Hospital (AJCH). Unlike previous studies which focused on specific homogeneous populations (mainly Saudis [7] and Qataris [8]) from this region, our center is located within Dubai, a regionally accessible city with high population diversity, which enabled us to recruit, genetically diagnose, and care for patients with rare diseases from underserved populations, representing at least 41 countries of the Middle East, Africa, and Asia, which have historically been underrepresented in genetic studies.

## Methods

### Study design and participants

This study includes patients referred for clinical genomic testing from April 2019 to November 2022. Patients’ physicians ordered the tests, and the physician or a certified genetic counselor explained the benefits, limitations, and risks of testing, and obtained written informed consent.

Peripheral blood samples were obtained from each patient and, in the case of trio whole-exome sequencing, their parents. Clinical data were provided by the referring physician either through the electronic medical record (internal patients) or on the requisition forms (outside patients). All clinical, demographic, and genetic data were summarized and reported by the laboratory molecular geneticist. The de-identified, aggregate reporting in this study was approved by the Dubai Healthcare Authority Research Ethics Committee (AJCH – 44), which determined that this study meets the exemption criteria with a waiver of informed consent. Case 297 was further enrolled and consented to an experimental study at the National Institutes of Health (NIH) (IRB Number: 00I0159).

### Exome sequencing

All testing was performed in our College of American Pathologists (CAP)-accredited genomics facility. Following DNA fragmentation by ultrasonication (Covaris, USA), the coding regions of the genome, also known as the exome, were captured using the Agilent Clinical Research Exome V2 (CREv2) capture probes (Agilent, USA). Libraries were prepared using the SureSelectXT protocol (Agilent, USA) and then sequenced (2 × 150 bp) using the NovaSeq system (Illumina, USA) to a minimum average depth of 100×^9^.

### Bioinformatic analysis

Sequencing data were then processed using an in-house custom-made bioinformatics pipeline to retain high-quality sequencing reads across all coding regions. High-quality variants were annotated for allele frequency (using mainly the Genome Aggregation Database and the Greater Middle East variome database), predicted protein effects, and presence or absence in disease databases, such as ClinVar and the Human Gene Mutation Database (HGMD). Only rare variants (< 0.5% minor allele frequency if novel, and < 1% if present in disease databases) were retained for downstream filtration and analysis [9]. During the final interpretation of reportable variants, the analysts retrieved the variants’ allele frequencies from the Middle East Variation (MEV) database, which includes whole exome and genome data from 2116 individuals from the Middle East [10].

For indication-based analysis, only rare, known pathogenic, or novel variants in the relevant genes associated with the patient’s indication were retained for interpretation.

For whole-exome sequencing (WES), all known pathogenic variants in ClinVar/HGMD and novel loss-of-function variants in disease genes were retained. In addition, segregation analysis was performed for trio WES to identify dominant, de novo, homozygous, X-linked, and compound heterozygous variants [10]. Copy number variants (CNVs), mainly hemizygous or homozygous events, were called using normalized NGS read depth data as previously described [11–13]. Copy number changes detected by NGS were confirmed by microarrays, specific multiplex ligation-dependent probe amplification (MLPA), PCR and gel electrophoresis, or customized droplet digital PCR (ddPCR) assays.

### Chromosomal microarrays

Chromosomal microarrays were preformed using the Affymetrix CytoScanHD system and the Chromosome Analysis Suite (ChAS) (Thermo Fisher Scientific, USA). Variants were classified following ACMG-AMP recommendations. Losses larger than 200 kb (with ≥ 25 probes) or gains larger than 400 kb (with ≥ 50 probes) are reported, along with smaller variants of pathogenic potential. Regions of homozygosity (ROH) greater than 10 Mb on a single chromosome are reported. Smaller ROHs may be reported in regions associated with known imprinting disorders.

### Targeted genetic analysis

Targeted testing included droplet digital PCR (ddPCR) for *SMN1* and *SMN2* copy number determination (Bio-Rad, USA), triplet repeat (CGG) expansion analysis in the promoter region of the *FMR1* gene using the FragileX AmplideX® PCR kit (Asuragen, USA) and capillary electrophoresis (Thermo Fisher Scientific, USA), and methylation-specific multiplex ligation-dependent probe amplification (MS-MLPA) for Angelman, Prader Willi, Russel Silver, and Beckwith Weidman syndromes (MRC-Holland, The Netherlands).

### Variant interpretation and reporting

All retained sequence and copy number variants were classified following the American College of Medical Genetics and Genomics/Association for Molecular Pathology (ACMG-AMP) or the American College of Medical Genetics and Genomics/Clinical genome Resource (ACMG-ClinGen) variant interpretation guidelines, respectively [14, 15]. Pathogenic and likely pathogenic variants in genes relevant to the patients’ primary indications were reported and were considered diagnostic if the patient’s phenotype (based on physician’s notes and feedback), disease mechanism, and inheritance were all consistent. Clinically significant heterozygous variants in genes with recessive inheritance and all variants of uncertain significance relevant to patients’ primary indications were also reported, though were not considered diagnostic, leading to inconclusive reports. All diagnostic and uncertain variants in this study, and the applied ACMG codes for each, can be found in Additional file 1.

When identified, medically actionable secondary findings were reported if patients opted in for such findings. Only pathogenic or likely pathogenic variants in the 59 genes recommended by ACMG (ACMG SFv2.0) were reported from April 2019 to February 2022. Subsequently, the 73 ACMG recommended gene list (ACMG SFv3.0) was implemented [16, 17].

### Clinical indications

The indications for genomic testing were categorized into 11 major groups based on involved systems(s). Since many patients have a combination of neurological and neurodevelopmental disorders, we combined both into a single group. For “complex multiple systemic involvement,” we gathered all the cases that can be under more than 2 groups. Finally, for a minority of patients who did not fit into any category, we grouped them as “others.”

### Clinical utility

For patients with diagnostic findings, we documented the changes in the management plans—either management or intervention—that occurred or were recommended after obtaining the genetic results. “Intervention” is defined as any planned process applied to the patient that requires medication introduction, change or discontinuation, diagnostic imaging, further testing, or surgical procedure. “Management” is defined as any planned process applied to the patient that does not fit the definition of “intervention,” including recommendations or advice, referral to other services, or follow-up plans.

### Statistical analyses

Variables, mainly diagnostic yields of different testing modalities, were compared using the Fisher exact test. Tests were 2-tailed, and *P* < .05 was considered statistically significant.

## Results

### Demographic and clinical characteristics of patients

**Fig. 1:**
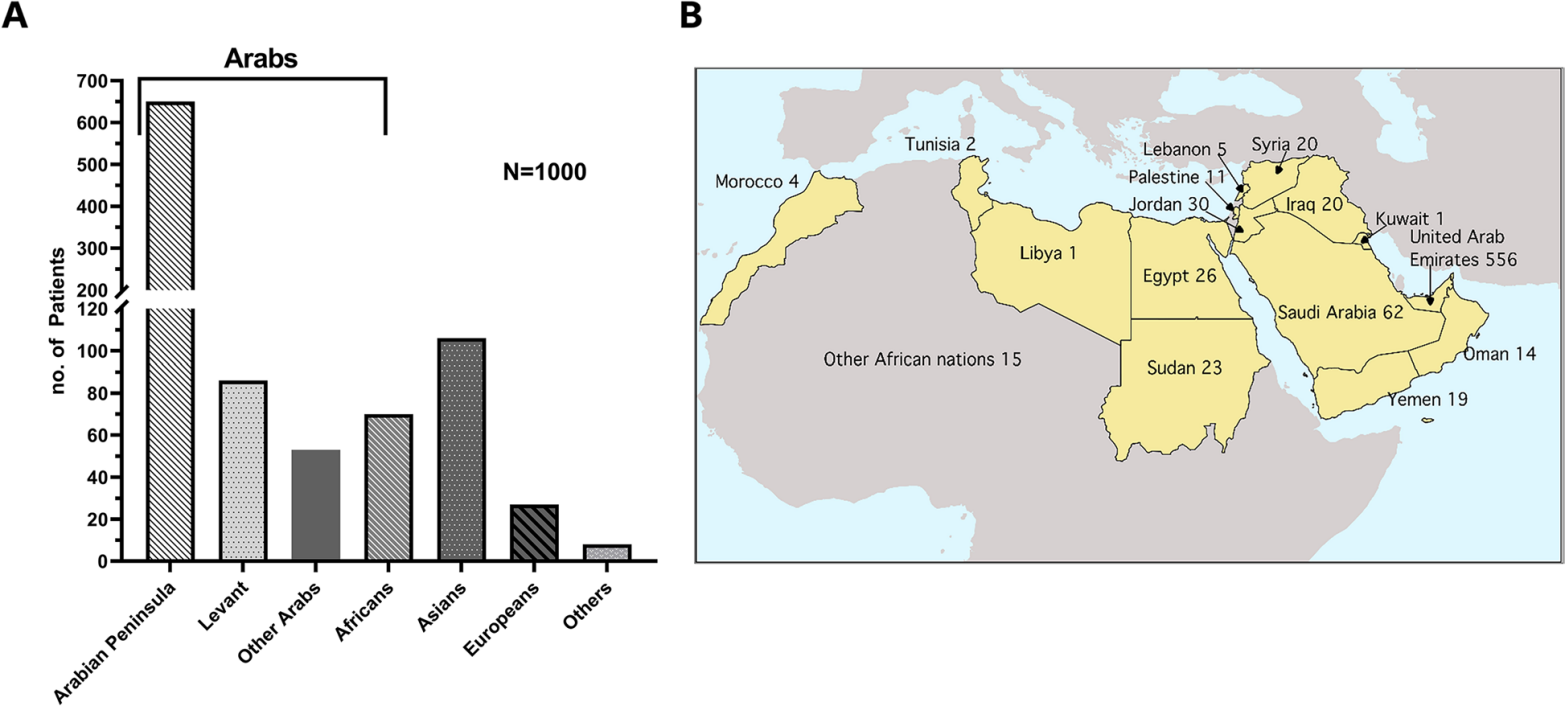
Cohort summary. **A** Distribution of patients’ origins by geographical regions. The bracket denotes patients of Arab origins, in the Middle East and North Africa, whose distribution by country is shown in **B**. Note that other Arabs (*N* = 53) of unknown countries of origin in **A** are not included on the map

A thousand patients (46.2% females; average age, 4.6 years) representing 47 countries (Additional file 1: Table S1) in the Arabian Peninsula (65%), the Levant (8.6%), Africa (7.6%), Asia (10.6%), and other geographic regions (8.2%) (Fig. 1A, B and Table 1) either presented (*N* = 857) or had their genetic testing referred (*N* = 143) to AJCH. Most patients (43.1%) were between 0 and 2 years, while 20.3% were 2–5 years and 36.6% ≥ 5 years of age (Table 1).

**Table 1:** Patient demographic information and ancestry

	All (***n***; %)	Arabs								Non-Arabs					
		Arabian Peninsula		Levant		North Africans		Others Unspecified		Asians		Europeans/North Americans		Africans/others	
		Total no.	Proportion% (95% CI)	Total no.	Proportion% (95% CI)	Total no.	Proportion% (95% CI)	Total no.	Proportion% (95% CI)	Total no.	Proportion% (95% CI)	Total no.	Proportion% (95% CI)	Total no.	Proportion% (95% CI)
**Age, years**															
**0–2**	432; 43.1%	246	56.9% (52.2–61.5)	44	10.2% (7.7–13.4)	25	5.8% (4.0–8.4)	32	7.4% (5.3–10.3)	57	13.2% (10.3–16.7)	16	3.7% (2.3–5.9)	12	2.8% (1.6–4.8)
**2–5**	203; 20.3%	142	70.0% (63.3–75.8)	19	9.4% (6.1–14.2)	13	6.4% (3.8–10.6)	8	3.9% (2.0–7.6)	14	6.9% (4.2–11.2)	4	2.0% (0.8–5.0)	3	1.5% (0.5–4.3)
**≥ 5**	365; 36.6%	262	71.8% (67.0–76.2)	23	6.3% (4.2–9.3)	23	6.3% (4.2–9.3)	13	3.6% (2.1–6.0)	35	9.6% (7.0–13.0)	9	2.5% (1.3–4.6)	0	0
**Sex**															
**Male**	538; 53.8%	350	65.1% (60.9–69.0)	45	8.4% (6.3–11.0)	39	7.2% (5.3–9.8)	32	5.9% (4.2–8.3)	49	9.1% (7.0–11.8)	15	2.8% (1.7–4.5)	8	1.5% (0.8–2.9)
**Female**	462; 46.2%	300	64.9% (60.5–69.1)	41	8.9% (6.6–11.8)	22	4.8% (3.2–7.1)	21	4.5% (3.0–6.8)	57	12.3% (9.6–15.7)	14	3.0% (1.8–5.0)	7	1.5% (0.7–3.1)
**Overall**	**1000**	**650**	**65.0%** (62.0–67.9)	**86**	**8.6%** (7.0–10.5)	**61**	**6.1%** (4.8–7.8)	**53**	**5.3%** (4.1–6.9)	**106**	**10.6%** (8.8–12.7)	**29**	**2.9%** (2.0–4.1)	**15**	**1.5%** (0.9–2.5)

Overall, 54% of patients presented with neurological phenotypes (Table 2). Comprehensive genomic sequencing-based testing (55.6%), in the form of whole or indication-based exome sequencing (Table 2 and Additional file 1: Table S2), or chromosomal microarrays (32.6%) were the most common clinical genomic investigations. Targeted testing, mainly for spinal muscular atrophy (SMA), fragile X, or methylation disorders, was performed for 29.1% of patients. Around 15.4% of patients (154 out of 1000) had a combination of more than one test (Table 2). AJCH is a referral center for SMA rapid testing and gene therapy in the Middle East: 154 of our patients received rapid *SMN1/2* analysis (Additional file 2: Fig. S1), and a subset (*N* = 12) received additional testing.

**Table 2:** Testing modality per clinical indication

	NGS		CMA		***P*** Value^a^	SMA		Fragile X		MS-MLPA/MLPA		Multiple testing		Overall^b^	
	Total no.	%Positive (95% CI)	Total no.	%Positive (95% CI)		Total no.	%Positive (95% CI)	Total no.	%Positive (95% CI)	Total no.	%Positive (95% CI)	Total no.	%Positive (95% CI)	Total no.	%Positive (95% CI)
**Clinical indication**															
Neurological	223	39.5% (33.3–46.0)	190	15.3% (10.8–21.1)	**0.0001**	154	33.1% (26.2–40.9)	103	1.0% (0.2–5.3)	20	45.0% (25.8–65.8)	132	31.1% (23.8–39.4)	540	32.2% (28.4–36.3)
Non-neurological	333	36.9% (31.9–42.2)	136	19.9% (14.0–27.3)	**0.0003**	0	0.0%	3	0.0%	11	27.3% (9.7–56.6)	22	40.9% (23.3–61.3)	460	32.8% (28.7–37.2)
**Overall**	556	37.9% (34.0–42.1)	326	17.2% (13.5–21.6)	**0.0001**	154	33.1% (26.2–40.9)	106	0.9 % (0.2–5.2)	31	38.7% (23.7–56.2)	154	32.5% (25.6–40.2)	1000	32.5% (29.7–35.5)

^a^Comparisons between NGS and CMA diagnostics yields

^b^Overall count per patient; therefore, the sum/percentage will differ due to multiple tests performed per patient

The analyses presented below focus on patients who received comprehensive genomic investigations (*N* = 882) for whom primary clinical indications were neurological/neurodevelopmental (25%), multisystem involvement (including the nervous system) (36.8%), dysmorphic structural defects (8.1%), and inflammatory disorders (4.5%). The remaining 25.3% of patients were referred for other primary indications including pulmonary, gastroenterology, sensory (vision and hearing), or hematological disorders (Additional file 1: Table S3).

### Genomic testing outcomes

**Fig. 2:**
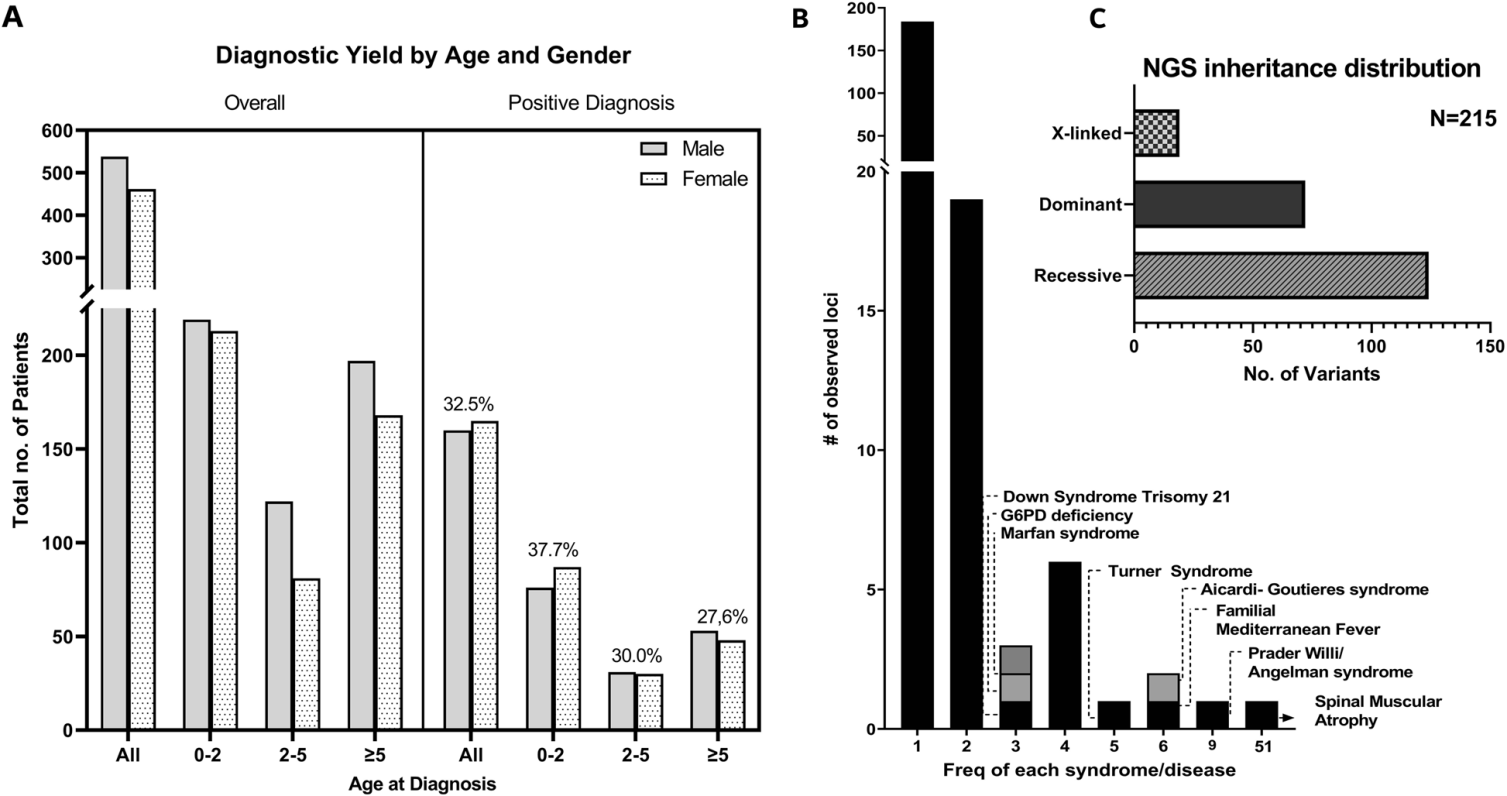
Diagnostic yield, rare disease frequency, and inheritance patterns. **A** Distribution of patients and diagnostic yield by age and gender. **B** Frequencies of rare diseases (*N* = 221) observed in this cohort. Most diseases (> 180) are seen only once. **C** Breakdown of inheritance patterns of diagnoses for cases with positive findings by sequencing (six cases had dual diagnoses and were counted twice in this figure)

Of the 1000 probands, 325 received a positive molecular finding (Additional file 1: Table S4) for an overall diagnostic yield of 32.5% (95% CI, 29.7–35.5) (Fig. 2A), which was higher for sequencing-based testing compared to chromosomal microarrays (37.9%; 95% CI, 34–42.1% versus 17.2%; 95% CI, 13.5–21.6%, respectively, *P* = 0.0001) across all indications (Table 2 and Additional file 1: Table S3). Specifically, a molecular diagnosis was more likely to be obtained by sequencing relative to microarrays for patients presenting with neurological disorders (38.5%; 95% CI, 30.4–47.4% for sequencing versus 11.1%; 95% CI, 6.3–18.8% for microarrays, *P* = 0.0001) or multisystem disease (42.4%; 95% CI, 35.5–49.6% for sequencing versus 22.7%; 95% CI, 16.6–30.3% for microarrays, *P* = 0.0002) (Table 2 and Additional file 1: Table S3).

Of these 325 cases with positive findings, 51 (15.7%) were originally referred for a combination of different testing modalities, yet sequencing was required to identify diagnostic pathogenic variants in most of those cases (36 cases, 70.5%) (Additional file 1: Table S4).

Most patients with positive genetic findings were less than 5 years of age (70.2%). Younger patients between 0 and 2 years of age tended to have a significantly higher diagnostic yield relative to patients who are 5 years or older (37.7%; 95% CI, 33.3–42.4% versus 27.7%; 95% CI, 23.3–32.5%, respectively, *P* = 0.0032) (Fig. 2A and Table 3).

**Table 3:** Molecular diagnosis rates by age and phenotype groups

	Neurological		Non-neurological		Overall	
	Total no.	%Positive (95% CI)	Total no.	%Positive (95% CI)	Total no.	%Positive (95% CI)
**Age, years**						
**0–2**	223	40.4% (34.1–46.9)	209	34.9% (28.8–41.6)	432	37.7% (33.3–42.4)
**2–5**	132	28.0% (21.1–36.2)	71	33.8% (23.9–45.4)	203	30.0% (24.2–36.7)
**≥ 5**	185	25.4% (19.7–32.1)	180	30.0% (23.8–37.1)	365	27.7% (23.3–32.5)
**Overall**	540	32.2% (28.4–36.3)	460	32.8% (28.7–37.2)	1000	32.5% (29.7–35.5)

### Genetic findings

A total of 221 rare diseases had an underlying genetic cause; the majority of these (*N* = 184) were observed only once. Thirty-two disorders were seen 2 to 9 times (Fig. 2B and Additional file 1: Table S5). Overall, the diagnostic variants were attributed to 180 unique genes/loci and 41 genomic intervals by CMA. All patients referred for SMA testing (*N* = 154), received rapid results within 4 days, on average, and a molecular diagnosis was made for 51 patients (33%) (Additional file 2: Fig. S1).

Of the 211 cases with positive findings by sequencing (Additional file 1: Table S6), 121 (57.3%) had biallelic pathogenic variants in genes associated with autosomal recessive disease (Table 4 and Fig. 2C). Homozygous pathogenic variants were the most common in this group (100 out of 121 cases, 82.6%). On the other hand, 66 out of the 211 cases (31.3%) carried heterozygous pathogenic variants, including 16 confirmed de novo and 2 mosaic variants, in genes causing autosomal dominant disease. X-linked findings were reported in 17 cases (8%) (Table 4 and Fig. 2C). Small exon-level copy number changes, detected by next-generation sequencing read depth, were reported in 9 patients in this group (4.3%). Six patients (2.8%) had dual diagnoses, although 4 of those patients were diagnosed by whole-exome sequencing bringing the dual diagnoses rate to 6.25% (4 out of total 64 positive cases) by whole-exome sequencing. A medically actionable secondary finding met the ACMG criteria for reporting in 2 out of 187 families (1%) undergoing whole-exome sequencing and consenting to receiving such findings (Table 4 and Additional file 1: Table S6). Finally, of the 235 clinically significant sequence variants identified in this group, 67 (28.5%) were novel or not previously reported in global disease databases (Additional file 1: Table S6).

**Table 4:** Molecular findings

Exome sequencing	
Mode of inheritance	No. of cases
Autosomal dominant (*n* = 66)	
Heterozygous SNV/INDELs	
De novo	16
Inherited	6
Unknown	41
Mosaic heterozygous SNV	2
Heterozygous CNV	1
Autosomal Recessive (*n* = 121)	
Homozygous SNV/INDELs	100
Compound heterozygous SNV/INDELs	15
Homozygous CNV	5
X-linked recessive/X-linked dominant (*n* = 17)	
Hemizygous CNV	
Inherited	1
Unknown	2
Hemizygous SNV	5
Heterozygous SNV	9
Two diagnosis (*n* = 6)	
Autosomal recessive + autosomal recessive	1
Autosomal dominant + autosomal dominant	2
Autosomal recessive + autosomal dominant	1
Autosomal recessive + X-linked	1
Autosomal dominant + X-linked	1
Risk factor	1
Secondary diagnosis	2
Chromosomal microarrays	
Copy number variants (*n* = 49)	
Heterozygous deletion	28
Homozygous deletion	1
Heterozygous duplication	5
Homozygous duplication	1
Partial trisomy	1
Partial mosaic tetrasomy	1
Whole-chromosome trisomy	8
Whole-chromosome tetrasomy	1
Whole-chromosome monosomy	2
Mosaic whole-chromosome trisomy and monosomy	1
Suspected unbalanced translocation (*n* = 4)	
Inverted duplication deletion	1
Inverted duplication deletion	1
Heterozygous mosaic deletion and heterozygous deletion	1
Unbalanced translocation	1
Two diagnoses (*n* = 1)	
Heterozygous deletion + heterozygous deletion	1
Loss of heterozygosity (*n* = 2)	
Uni-parental disomy^a^	2

^a^UPD7 (case 212) and UPD15 (case 438)

Of the 56 cases with positive microarray findings (Additional file 1: Table S7), 28 (50%) had single heterozygous pathogenic deletions. Complete or partial chromosomal aneuploidies and uniparental disomies were detected in 14 and 2 cases, respectively (Table 4). One patient had dual diagnoses due to two non-overlapping heterozygous deletions (Table 4 and Additional file 1: Table S7). Of the 326 cases referred for chromosomal microarrays, 120 (36.8%; 95% CI, 31.76–42.17%) had significant regions of homozygosity averaging 7.36% of the autosomal genome (Additional file 1: Table S8), a finding consistent with the prevalent close relative marriages. Interestingly, 105 out of the 120 patients (87.5%) with significant regions of homozygosity did not have conclusive findings by microarrays (Additional file 1: Table S8) suggesting that these cases can be candidates for recessive novel gene discovery. We highlight 8 cases with putative candidate genes (*SOAT2*, *AOX1*, *MYBPC2*, *CYP4X1*, *DTHD1*, *FBXO22*, *MAN2B2*, and *SATL1*) underlying novel recessive disorders in this cohort (Additional file 1: Table S9).

### Clinical utility

**Fig. 3:**
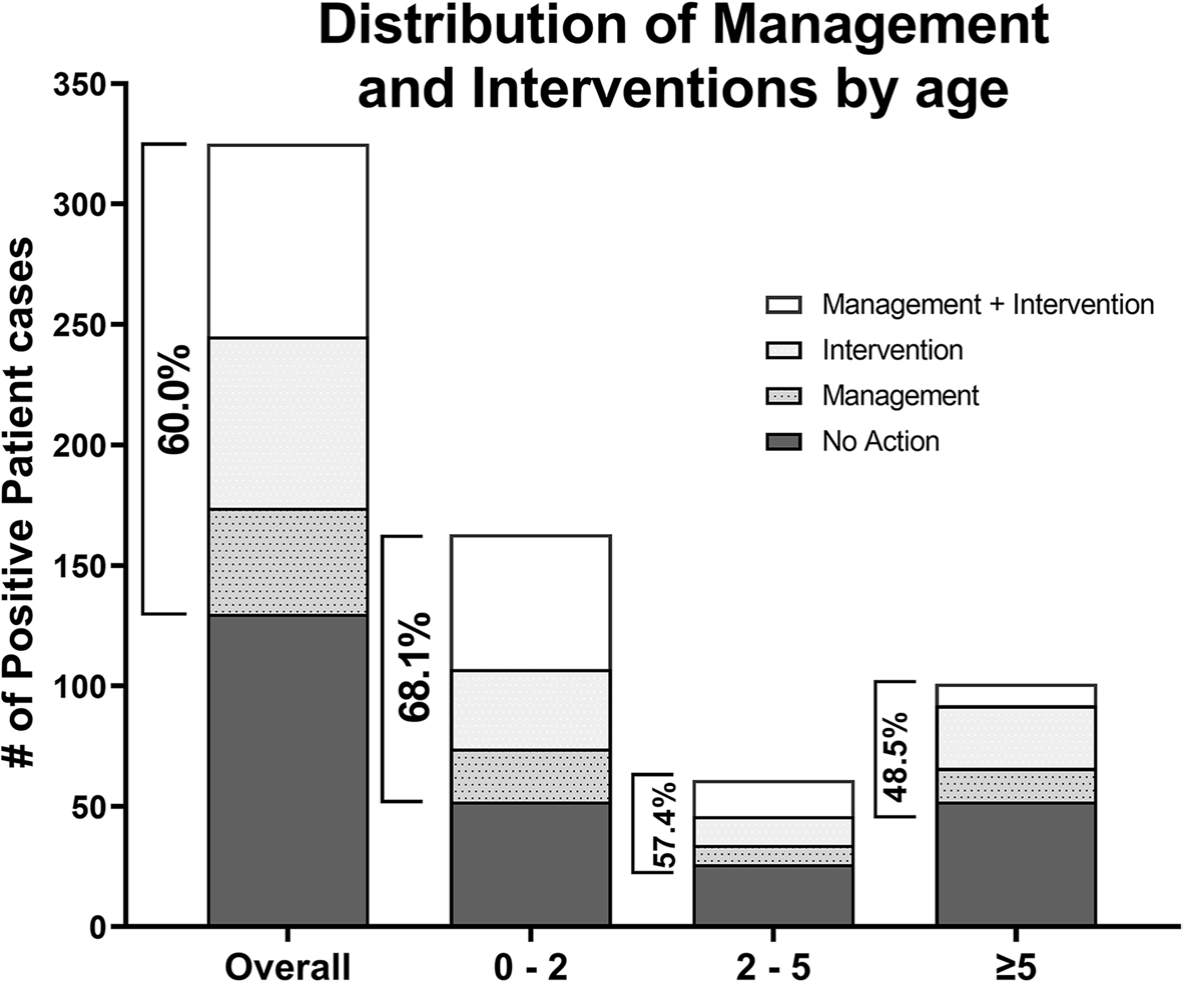
Age distribution of patients with positive findings who received modified management and/or intervention plans

Genetic findings offered new management and/or intervention plans for 195 out of the 325 cases (60%) with positive genetic findings across all ages (Fig. 3 and Additional file 1: Tables S6 and S7). All 51 patients with diagnostic SMA findings were referred for gene therapy (ZOLGENSMA®). Excluding SMA, genetic findings uncovered new interventions and/or management plans for the remaining 144 out of 274 positive cases (52.55%) as summarized in Additional file 1: Tables S6 and S7. Younger patients (0–2 years) were more likely to receive altered management and/or intervention plans (as defined in the “Methods” section) based on their positive genetic findings when compared to older patients (≥ 5 years) (68.1% versus 48.5%, *P* = 0.0019) (Fig. 3).

In addition to direct patient management, all families referred for genetic testing within AJCH (*N* = 857) were supported by certified genetic counselors for test selection, pre-test counseling, reporting, and/or post-test counseling. Families were therefore informed about their recurrence risks and options for avoidance in future pregnancies.

### Impact on diagnostic odyssey

Although most patients with positive genetic findings were relatively young (Fig. 2A), 78 of those patients (24%) were over 7 years of age (average 12.4 years, range 7–37 years) suggesting delayed diagnoses due to the lack of access to genomic services in this region. We present a case example to highlight this issue.

Case 297 is a 20-year-old male with a long history of recurrent infections including eczema and ear infections during early childhood and recurrent pneumonia later in life leading to bronchiectasis. He had consistently elevated serum IgE and eosinophils throughout his course of illness and was symptomatically managed, including recurrent hospitalizations, without a clear working diagnosis. This patient was referred to AJCH for indication-based exome sequencing which revealed a homozygous pathogenic variant (c.4241+1G>A) in the *DOCK8* gene which led to abnormal splicing (Additional file 1: Methods S1 and Additional file 3: Fig. S3). This finding ended over a 15-year-long diagnostic odyssey, and the patient was diagnosed with *DOCK8*-combined immunodeficiency syndrome, a life-threatening condition characterized by recurrent skin and respiratory infections, hyper IgE, and a high risk of developing blood or skin cancers. No treatments were available for this condition.

The patient was then transferred to the National Institute of Health (NIH) where additional investigations revealed that he also has non-Hodgkin’s lymphoma. He then underwent allogenic hematopoietic stem cell transplant from a healthy sibling. Post-transplant, previously present eczematous dermatitis, recurrent infections, and lymphoma were all resolved.

## Discussion

We present a cohort of highly diverse patients with rare diseases from 47 countries primarily within the Middle East, Africa, and Asia, a population which has been historically underserved in genomic services and underrepresented in global genetic studies. Our study demonstrates a high cumulative diagnostic yield (32.5%) for genomic investigations in this cohort. This yield was highest for sequencing-based testing (~ 38%); recessive (autosomal and X-linked) inheritance accounted for ~ 62% of sequencing diagnoses in this study, consistent with the expected burden of single gene disorders in this population. Genomic sequencing testing was consequently the most effective testing strategy across several clinical indications. Genetic findings frequently led to early diagnoses, indicating new management and/or intervention plans, besides offering families information about recurrence risks and options to avoid disease in future pregnancies.

Around 37% of all patients who received chromosomal microarray testing (*N* = 326) had significant regions of homozygosity, a finding that is highly expected given the presence of close relative, including first cousin and marriages in the Middle East. Such regions can be enriched in novel candidate recessive genes. In fact, we are currently pursuing further investigations of 8 cases with putative candidate genes underlying novel recessive disorders in this cohort (Additional file 1: Table S9).

Our overall sequencing-based testing diagnostic yield (~ 38%) was similar to that reported in another study which focused on Saudi patients [7], while our whole-exome sequencing diagnostic yield was higher than that previously reported in other populations (34% versus 25% [18, 19] to 29% [20]). On the other hand, a substantial number of patients (27.1%; 95% CI, 23.6–30.9%) also received inconclusive results due to sequence variants of uncertain clinical significance or VUSs (Additional file 1: Tables S2 and S10). Similarly, 29% (95 out of 326) of microarray cases were inconclusive due to VUSs (Additional file 1: Table S11). As expected, trio whole-exome sequencing had significantly less inconclusive findings relative to single or proband-only exome sequencing in our setting (32.5% inconclusive rate for trio sequencing versus 55.2% for single exome sequencing, *P* = 0.03). On the other hand, the inconclusive rate was also significantly lower for indication-based panels (22.5%) compared to all exome sequencing tests (36.4%; *P* = 0.0006). These findings suggest that targeted panels, and trio whole-exome sequencing for complex indications, should be the preferred testing approaches in our setting (and likely any other settings as well). Nonetheless, this rate of VUSs (> 20%) highlights the need for greater collection of sequencing data from this region—currently underrepresented in global genetic databases [5, 6]—which will allow us to better characterize both the “benign” and “disease” variation landscape, therefore improving variant classification and overall genetic data interpretation.

Despite the utility of genomic testing in our setting, this service is largely inaccessible to patients in the region due to a lack of specialized centers with local genomic services. Furthermore, aside from Emirati citizens, who have free access to public healthcare services in the UAE, expatriate patients with private health insurance plans often do not get coverage for genetic testing. Of 373 genetic tests ordered for non-Emirati patients, the overall reimbursement rate was 16.4% and was lowest for whole-exome sequencing (8.2%). A limited understanding of the importance of genetic testing in the management of rare diseases in general, and specifically in this population, likely contributes to this low reimbursement rate, a trend we believe is prevalent in other countries with limited access to specialized services. In addition to studies demonstrating the clinical utility of genomic investigations in this population, as we show in this cohort, more studies are needed to quantify the economic value of implementing genomic investigations to diagnose and guide the management of patients with rare diseases. Eventually, all such studies will strengthen the case for reimbursement of genomics workup in this population.

## Conclusions

Our study demonstrates that clinical genomic investigations should become the standard of care for patients with rare diseases in this patient population. However, significant local investments are needed to establish multidisciplinary specialized centers where genomic investigations and subsequent management and intervention plans are accessible for patients with rare disorders. Furthermore, integrated research programs are essential to characterize the novel disorders in this population and to expand the clinical annotation of the human genome. It is important to note that the reported diagnostic yield in this study is based on gene-disease associations primarily established in other populations. We therefore expect this yield will increase over time as more novel genes are characterized through the study of this population. Establishing clinical genomics and research programs in this region will therefore not only benefit patients locally but will also enhance genomic data representation globally, expanding our understanding of the human genome.

## Supplementary Information

**Additional file 1: Methods S1. ***DOCK8* GENOMIC AND RNA ANALYSIS. **Table S1.** Patient’s Country-of-origin breakdown. **Table S2.** Outcomes and Breakdown of Comprehensive Genomic Sequencing-based Testing. **Table S3.** Clinical Indications for Comprehensive Genomic Testing. **Table S4.** 325 Positive Cases. **Table S5.** Disease Frequency. **Table S6.** Clinically significant sequence variants in patients with positive findings (N = 211). **Table S7.** Clinically significant microarrays variants in patients with positive findings (N = 56). **Table S8.** Cases with extended regions of loss of heterozygosity by microarrays. **Table S9:** Candidate genes. **Table S10.** Sequencing Variants of Uncertain Clinical Significance (VUS). **Table S11.** CMA Variants of Uncertain Clinical Significance (VUS).

**Additional file 2: Figure S1.** SpinalMuscular Atrophy (SMA) cohort (N = 138). **A**) Distribution of patient origins bycountry. Patients represent 19 countries; majority are Arabs mostly from SaudiArabi, United Arab Emirates, Iraq, Sudan, and Syria. **B**) Testing was positive in48 cases for a diagnostic yield of ~35%. **C**) Average Turnaround time was 4 days, with majority (61%) receiving results in ≤ 4days.

**Additional file 3: Figure S2.** Genomicand RNA analysis of *DOCK8* in case 297. **A**) Family pedigree. Filledsquare denotes affected proband (arrow); half filed squares/circles denote confirmed (female sibling) or obligate (parents) heterozygous carriers. Parentswere first cousins. **B**) PCR amplification and Sanger sequencing ofgenomic DNA, using primers targeting exon 33 and splice regions (table below),confirmed the *DOCK8* (NM_203447.3):c.4241+1G>A variant in thehomozygous state in case 297 and heterozygous state in the female sibling. **C**)cDNA from patient, his female sibling, and a control sample was synthesized andimpact of the *DOCK8* (NM_203447.3):c.4241+1G>A variant on splicing was assessed using PCR primers targeting exons 33 and 34 (table below). A normalPCR product with an expected size of 169bp was observed in thecontrol sample suggesting normal splicing between exons 33 and 34. The normalcDNA 169bp product was not detected in the patient suggesting abnormal splicingdue to the c.4241+1G>A variant. Instead, a cryptic cDNA product >300bp insize is identified in the patient. Female sibling carried both the normal(169bp) and cryptic (>300bp) products, a finding which is consistent withher heterozygous carrier status (panel B). A control PCR product (186bp) wasamplified from cDNA of all samples using primers targeting exons 5 and 6. Allprimer sequences are provided below.

## Data Availability

All data used and generated is available within the main manuscript and supporting files. All identified variants were submitted to ClinVar (Submission IDs: SUB10670505, SUB10465084, SUB8609948, SUB8556325, SUB8555056, SUB8155288, SUB12445851, SUB12446306, and SUB12446498).

## Abbreviations

AJCH: Al Jalila Children’s Specialty Hospital
NIH: National Institutes of Health
CAP: College of American Pathologists
CREv2: Clinical Research Exome V2
HGMD: Human Gene Mutation Database
MEV: Middle East Variation
WES: Whole-exome sequencing
CNVs: Copy number variants
MLPA: Multiplex ligation-dependent probe amplification
ddPCR: Droplet digital PCR
ChAS: Chromosome Analysis Suite
ROH: Regions of homozygosity
MS-MLPA: Methylation-specific multiplex ligation-dependent probe amplification
ACMG-AMP: American College of Medical Genetics and Genomics/Association for Molecular Pathology
ACMG-ClinGen: American College of Medical Genetics and Genomics/Clinical genome Resource
NGS: Next-generation sequencing
CMA: Chromosomal microarray
SMA: Spinal muscular atrophy
