# Supplementary material for: The genomic landscape of rare disorders in the Middle East
Source: Genome Med. 2023 Jan 27;15:5. doi: 10.1186/s13073-023-01157-8 (PMC9881316; doi:10.1186/s13073-023-01157-8)
Supplement: Supplementary file 1 — Additional file 1: Methods S1. DOCK8 GENOMIC AND RNA ANALYSIS. Table S1. Patient’s Country-of-origin breakdown. Table S2. Outcomes and Breakdown of Comprehensive Genomic Sequencing-based Testing. Table S3. Clinical Indications for Comprehensive Genomic Testing. Table S4. 325 Positive Cases. Table S5. Disease Frequency. Table S6. Clinically significant sequence variants in patients with positive findings (N = 211). Table S7. Clinically significant microarrays variants in patients with positive findings (N = 56). Table S8. Cases with extended regions of loss of heterozygosity by microarrays. Table S9: Candidate genes. Table S10. Sequencing Variants of Uncertain Clinical Significance (VUS). Table S11. CMA Variants of Uncertain Clinical Significance (VUS). [file 13073_2023_1157_MOESM1_ESM.docx]

| **Methods S1**  *DOCK8* GENOMIC AND RNA ANALYSIS  DNA and RNA extracted from case 297 and his sibling respectively underwent Sanger sequencing and cDNA synthesis followed by amplification and gel electrophoresis. Refer to **Additional file 3: Fig. S2** legend for more details. *DOCK8* primers are listed below.   \| **Oligo Name** \| **Sequence (M13 sequences in red)** \| **Amplicon Size (bp)** \| \| --- \| --- \| --- \| \| \| DOCK8.FWD.EX33-34* \| GTAAAACGACGGCCAGTAACGACCGATTTCCAGGCCT \| 169 \| \| DOCK8.REV.EX33-34* \| CAGGAAACAGCTATGACCCCTGCATATCCAGGATGA \| 169 \| \| DOCK8.FWD.EX5-6* \| GTAAAACGACGGCCAGTCACAAGACGCTTCCGAAACA \| 187 \| \| DOCK8.REV.EX5-6* \| CAGGAAACAGCTATGACCCTTGCTGCAGGAGGTTTTCTAG \| 187 \| \| DOCK8.EXON.33.FWD** \| GTAAAACGACGGCCAGTTCTTGGAGGGTTTCATGCTAA \| 243 \| \| DOCK8.EXON.33.REV** \| CAGGAAACAGCTATGACCTAACCCTTGGAAGGATGCAG \| 243 \|   *Primers used for cDNA analysis; **primers used for Sanger sequencing of genomic DNA.  **Additional file 2: Fig. S1**. Spinal Muscular Atrophy (SMA) cohort (N = 138). A) Distribution of patient origins by country. Patients represent 19 countries; majority are Arabs mostly from Saudi Arabi, United Arab Emirates, Iraq, Sudan, and Syria. B) Testing was positive in 48 cases for a diagnostic yield of ~35%. C) Average Turnaround time was 4 days, with majority (61%) receiving results in ≤ 4days.  **Additional file 3: Fig. S2**. Genomic and RNA analysis of *DOCK8* in case 297. **A**) Family pedigree. Filled square denotes affected proband (arrow); half filed squares/circles denote confirmed (female sibling) or obligate (parents) heterozygous carriers. Parents were first cousins. **B**) PCR amplification and Sanger sequencing of genomic DNA, using primers targeting exon 33 and splice regions (table below), confirmed the *DOCK8* (NM_203447.3):c.4241+1G>A variant in the homozygous state in case 297 and heterozygous state in the female sibling. **C**) cDNA from patient, his female sibling, and a control sample was synthesized and impact of the *DOCK8* (NM_203447.3):c.4241+1G>A variant on splicing was assessed using PCR primers targeting exons 33 and 34 (table below). A normal PCR product with an expected size of 169bp was observed in the control sample suggesting normal splicing between exons 33 and 34. The normal cDNA 169bp product was not detected in the patient suggesting abnormal splicing due to the c.4241+1G>A variant. Instead, a cryptic cDNA product >300bp in size is identified in the patient. Female sibling carried both the normal (169bp) and cryptic (>300bp) products, a finding which is consistent with her heterozygous carrier status (panel B). A control PCR product (186bp) was amplified from cDNA of all samples using primers targeting exons 5 and 6. All primer sequences are provided below.   \| **Table S1. Patient’s Country-of-origin breakdown** \| \| \| \| --- \| --- \| --- \| \| Nationality \| No of patients \| Region \| \| Emirati \| 556 \| Arabian Peninsula \| \| Saudi \| 60 \| Arabian Peninsula \| \| Arabs of Unknown Origin \| 43 \| Others - Arabs \| \| Pakistani \| 37 \| Asians \| \| Indian \| 36 \| Asians \| \| Jordanian \| 30 \| Levant \| \| Egyptian \| 26 \| North Africa \| \| Sudanese \| 23 \| North Africa \| \| Iraqi \| 20 \| Levant \| \| Syrian \| 20 \| Levant \| \| Yemini \| 19 \| Arabian Peninsula \| \| Omani \| 14 \| Arabian Peninsula \| \| Palestinian \| 11 \| Levant \| \| British \| 10 \| Europeans \| \| Comoron \| 10 \| Others- Arabs \| \| Filipino \| 9 \| Asians \| \| Other Asians \| 6 \| Asians \| \| Iranian \| 6 \| Asians \| \| Afghan \| 5 \| Asians \| \| Lebanese \| 5 \| Levant \| \| Serbian \| 4 \| Europeans \| \| Turkish \| 4 \| Europeans \| \| Moroccan \| 4 \| North Africa \| \| Somali \| 4 \| North Africa \| \| Other - Non Arab \| 4 \| Others - non Arabs \| \| Nigerian \| 3 \| Africans \| \| Russian \| 3 \| Europeans \| \| Tanzanian \| 2 \| Africans \| \| Ugandan \| 2 \| Africans \| \| Sri Lankan \| 2 \| Asians \| \| Italian \| 2 \| Europeans \| \| Tunisian \| 2 \| North Africa \| \| Kenyan \| 1 \| Africans \| \| Ethiopian \| 1 \| Africans \| \| Kuwaiti \| 1 \| Arabian Peninsula \| \| Bangladishi \| 1 \| Asians \| \| Japanese \| 1 \| Asians \| \| Maldivian \| 1 \| Asians \| \| Indonesian \| 1 \| Asians \| \| Tajikistani \| 1 \| Asians \| \| Dutch \| 1 \| Europeans \| \| Irish \| 1 \| Europeans \| \| Polish \| 1 \| Europeans \| \| Ukranian \| 1 \| Europeans \| \| Libyan \| 1 \| North Africa \| \| Algerian \| 1 \| North Africa \| \| Brazilian \| 1 \| South America \| \| Canadian \| 1 \| North America \| \| New Zealander \| 1 \| Others - non Arabs \| \| Vanuatu \| 1 \| Others - non Arabs \| |
| --- | --- | --- | --- | --- | --- | --- | --- | --- | --- | --- | --- | --- | --- | --- | --- | --- | --- | --- | --- | --- | --- | --- | --- | --- | --- | --- | --- | --- | --- | --- | --- | --- | --- | --- | --- | --- | --- | --- | --- | --- | --- | --- | --- | --- | --- | --- | --- | --- | --- | --- | --- | --- | --- | --- | --- | --- | --- | --- | --- | --- | --- | --- | --- | --- | --- | --- | --- | --- | --- | --- | --- | --- | --- | --- | --- | --- | --- | --- | --- | --- | --- | --- | --- | --- | --- | --- | --- | --- | --- | --- | --- | --- | --- | --- | --- | --- | --- | --- | --- | --- | --- | --- | --- | --- | --- | --- | --- | --- | --- | --- | --- | --- | --- | --- | --- | --- | --- | --- | --- | --- | --- | --- | --- | --- | --- | --- | --- | --- | --- | --- | --- | --- | --- | --- | --- | --- | --- | --- | --- | --- | --- | --- | --- | --- | --- | --- | --- | --- | --- | --- | --- | --- | --- | --- | --- | --- | --- | --- | --- | --- | --- | --- | --- | --- | --- | --- | --- | --- | --- | --- | --- | --- | --- | --- | --- | --- | --- |

| **Table S2. Outcomes and Breakdown of Comprehensive Genomic Sequencing-based Testing** | | | | | | | | | | | |
| --- | --- | --- | --- | --- | --- | --- | --- | --- | --- | --- | --- |
|  |  |  | All | Positive | |  | Negative | |  | Inconclusive | |
|  |  |  |  | Total no. | Rate% |  | Total no. | Rate% |  | Total no. | Rate% |
|  |  |  |  |  | (95% CI) |  |  | (95% CI) |  |  | (95% CI) |
| Next Generation Sequencing Test Types | | |  |  |  |  |  |  |  |  |  |
|  | Indication based exomes/panels | | 377 | 147 | 39.0% |  | 145 | 38.5% |  | 85 | 22.5% |
|  |  |  |  |  | (34.2 - 44.0) |  |  | (33.7 - 43.5) |  |  | (18.6 - 27.0) |
|  | Single WES/WESTRIO/WESQUAD | | 187 | 64 | 34.2% |  | 55 | 29.4% |  | 68 | 36.4% |
|  |  |  |  |  | (27.8 - 41.3) |  |  | (23.3 - 36.3) |  |  | (29.8 - 43.5) |
|  |  | Single WES | 29 | 8 | 27.6% |  | 5 | 17.2% |  | 16 | 55.2% |
|  |  |  |  |  | (14.7 - 45.7) |  |  | (7.6 - 34.5) |  |  | (37.5 - 71.6) |
|  |  | WES DUO | 2 | 1 | 50.0% |  | 0 | 0.0% |  | 1 | 50.0% |
|  |  |  |  |  | (9.5 - 90.5) |  |  | 0 |  |  | (9.5 - 90.5) |
|  |  | WESTRIO | 151 | 54 | 35.8% |  | 48 | 31.8% |  | 49 | 32.5% |
|  |  |  |  |  | (28.6 - 43.7) |  |  | (24.9 - 39.6) |  |  | (25.5 - 40.3) |
|  |  | WESQUAD | 5 | 1 | 20.0% |  | 2 | 40.0% |  | 2 | 40.0% |
|  |  |  |  |  | (3.6 - 62.4) |  |  | (11.8 - 76.9) |  |  | (11.8 - 76.9) |
| Overall | | | 564 | 211 | 37.4% |  | 200 | 35.5% |  | 153 | 27.1% |
|  |  |  |  |  | (33.5 - 41.5) |  |  | (31.6 - 39.5) |  |  | (23.6 - 30.9) |

| **Table S3. Clinical Indications for Comprehensive Genomic Testing** | | | | | | | | |
| --- | --- | --- | --- | --- | --- | --- | --- | --- |
|  |  | **NGS** | |  | **CMA** | | P Value |  |
|  |  | Total no. | %Positive  (95% CI) |  | Total no. | %Positive  (95% CI) |  |  |
| **Clinical Indication** | |  |  |  |  |  |  |  |
|  | Neurological or Neurodevelopmental | 122 | 38.5% |  | 99 | 11.1% | **0.0001** |  |
|  |  |  | (30.4 - 47.4) |  |  | (6.3 - 18.8) |  |  |
|  | Complex multiple systemic involvement | 184 | 42.4% |  | 141 | 22.7% | **0.0002** |  |
|  |  |  | (35.5 - 49.6) |  |  | (16.6 - 30.3) |  |  |
|  | Dysmorphic/Structural defect | 22 | 40.9% |  | 50 | 22.7% | 0.15 |  |
|  |  |  | (23.3 - 61.3) |  |  | (5.6 - 23.8) |  |  |
|  | Inflammatory process | 40 | 35.0% |  | 1 | 100% | NA |  |
|  |  |  | (22.1 - 50.5) |  |  | (20.7 - 100) |  |  |
|  | Growth/ Maturation disturbance | 17 | 23.5% |  | 17 | 23.5% | 1.00 |  |
|  |  |  | (9.6 - 47.3) |  |  | (9.6 - 47.3) |  |  |
|  | Visual disturbance | 20 | 55.0% |  | 2 | 50.0% | NA |  |
|  |  |  | (34.2 - 74.2) |  |  | (9.5 - 90.5) |  |  |
|  | Pulmonology | 16 | 31.3% |  | 4 | 25.0% | 1.00 |  |
|  |  |  | (14.2 - 55.6) |  |  | (4.6 - 69.9) |  |  |
|  | Gastroenterological | 28 | 21.4% |  | 0 | 0 | NA |  |
|  |  |  | (10.2 - 39.5) |  |  |  |  |  |
|  | Haematology | 20 | 40.0% |  | 0 | 0 | NA |  |
|  |  |  | (21.9 - 61.3) |  |  |  |  |  |
|  | Hearing disturbance | 11 | 54.5% |  | 0 | 0 | NA |  |
|  |  |  | (28.0 - 78.7) |  |  |  |  |  |
|  | Others | 76 | 30.3% |  | 12 | 0.0% | NA |  |
|  |  |  | (21.3 - 41.3) |  |  |  |  |  |
| **Overall** | | 556 | 37.9% |  | 326 | 17.2% | **0.0001** |  |
|  |  |  | (34.0 - 42.1) |  |  | (13.5 - 21.6) |  |  |

| **Table S4. 325 Positive Cases** | | | | | | |
| --- | --- | --- | --- | --- | --- | --- |
| Age At Diagnostic Test (Yrs) | Region | NGS Results | CMA Results | SMN1 Results | FMR1 Results | MSMLPA/MLPA Results |
| ≥5 | GCC |  | Positive |  |  |  |
| 2-5 | North Africans |  |  | Positive |  |  |
| ≥5 | GCC |  | Positive |  |  |  |
| 0-2 | GCC |  |  | Positive |  |  |
| 0-2 | GCC |  |  | Positive |  |  |
| 2-5 | GCC | Positive |  |  |  |  |
| ≥5 | Levant | Positive |  |  |  |  |
| 0-2 | North Africans | Positive |  |  |  |  |
| 0-2 | Asian | Positive |  |  |  |  |
| 0-2 | Asian | Positive |  |  |  |  |
| ≥5 | Asian | Positive |  |  |  |  |
| 0-2 | GCC |  |  | Positive |  |  |
| 0-2 | North Africans | Positive |  |  |  |  |
| ≥5 | GCC | Positive |  |  |  |  |
| 0-2 | North Africans | Positive |  |  |  |  |
| 0-2 | GCC |  |  | Positive |  |  |
| 0-2 | GCC | Positive |  |  |  |  |
| 0-2 | Other Arab | Positive |  |  |  |  |
| 0-2 | Africans |  |  | Positive |  |  |
| ≥5 | GCC | Positive |  |  |  |  |
| 2-5 | Other | Positive |  |  |  |  |
| 0-2 | North Africans |  | Positive |  |  |  |
| ≥5 | Levant | Positive |  |  |  |  |
| 2-5 | GCC | Positive |  |  |  |  |
| 2-5 | GCC |  |  | Positive |  |  |
| 0-2 | North Africans | Positive | Negative |  | Negative |  |
| ≥5 | North Africans | Positive |  |  |  |  |
| ≥5 | North Africans | Positive |  |  |  |  |
| 0-2 | GCC | Positive |  |  |  |  |
| 0-2 | Asian | Positive |  |  |  |  |
| ≥5 | GCC | Positive | Inconclusive |  |  |  |
| 0-2 | GCC |  | Positive | Negative |  | Positive |
| ≥5 | Levant |  | Positive |  |  |  |
| 0-2 | GCC | Positive |  |  |  |  |
| 0-2 | GCC |  |  | Positive |  |  |
| 2-5 | GCC |  |  | Positive |  |  |
| 0-2 | GCC |  |  | Positive |  |  |
| 0-2 | Other | Positive | Negative |  |  |  |
| 0-2 | Asian | Positive |  |  |  |  |
| ≥5 | GCC |  | Positive |  | Negative |  |
| ≥5 | Asian | Positive |  |  |  |  |
| 0-2 | Levant | Positive |  |  |  |  |
| 0-2 | GCC |  |  | Positive |  |  |
| 0-2 | GCC | Positive |  | Negative |  |  |
| 0-2 | GCC |  |  | Positive |  |  |
| 0-2 | GCC | Inconclusive | Positive | Negative |  |  |
| 2-5 | GCC | Positive |  |  |  |  |
| 0-2 | Asian |  |  | Positive |  |  |
| 2-5 | Other Arab | Positive |  |  |  |  |
| 0-2 | Other Arab | Positive | Inconclusive |  |  |  |
| ≥5 | Levant | Positive |  |  |  |  |
| ≥5 | GCC | Positive | Inconclusive |  |  |  |
| ≥5 | GCC | Positive |  |  |  |  |
| 0-2 | GCC | Positive |  |  |  |  |
| ≥5 | Levant | Positive |  |  |  |  |
| ≥5 | GCC | Positive |  |  |  |  |
| 2-5 | Levant | Positive |  |  |  |  |
| ≥5 | GCC | Positive |  |  |  |  |
| 0-2 | GCC | Positive |  |  |  |  |
| 2-5 | Levant | Positive |  |  |  |  |
| 0-2 | GCC |  | Positive |  |  |  |
| 2-5 | Europeans | Positive |  |  |  |  |
| 2-5 | Levant |  | Positive |  | Negative | Positive |
| 0-2 | GCC | Positive | Inconclusive | Negative |  |  |
| 0-2 | Asian |  | Positive |  |  |  |
| ≥5 | GCC | Positive |  |  |  |  |
| 2-5 | GCC | Positive |  |  |  |  |
| 0-2 | Asian | Positive |  |  |  |  |
| 2-5 | GCC | Positive | Negative |  |  |  |
| 0-2 | GCC | Positive |  | Negative |  |  |
| 0-2 | GCC | Positive |  |  |  |  |
| 0-2 | GCC | Positive |  |  |  |  |
| 2-5 | Europeans | Positive |  |  |  |  |
| ≥5 | GCC | Positive |  |  |  |  |
| ≥5 | GCC |  |  |  | Positive |  |
| ≥5 | GCC | Positive |  |  |  |  |
| 0-2 | Asian |  | Positive |  |  |  |
| 2-5 | Asian |  | Positive |  |  |  |
| 0-2 | GCC | Negative | Positive |  |  |  |
| 0-2 | GCC | Positive | Positive |  |  |  |
| ≥5 | GCC | Positive |  |  |  |  |
| 2-5 | GCC | Positive | Inconclusive |  |  |  |
| 2-5 | GCC | Positive | Inconclusive |  | Negative |  |
| 0-2 | GCC |  | Positive |  |  |  |
| 0-2 | Europeans |  | Positive |  |  | Positive |
| 0-2 | Asian | Positive |  |  |  |  |
| 0-2 | Asian |  |  | Positive |  |  |
| ≥5 | GCC | Positive | Negative |  |  |  |
| ≥5 | GCC | Positive |  |  |  |  |
| 0-2 | GCC | Positive |  |  |  |  |
| 0-2 | GCC |  | Positive |  |  |  |
| 0-2 | Levant | Positive |  | Negative |  |  |
| 2-5 | GCC |  | Positive |  | Negative |  |
| 2-5 | GCC | Positive |  |  |  |  |
| 0-2 | North Africans |  |  | Positive |  |  |
| ≥5 | GCC | Positive |  |  |  |  |
| 0-2 | Levant | Positive |  |  |  |  |
| 0-2 | GCC | Positive | Inconclusive |  |  |  |
| 0-2 | GCC |  | Positive |  |  |  |
| ≥5 | Levant | Positive |  |  |  |  |
| ≥5 | Europeans | Positive |  |  |  |  |
| ≥5 | Levant | Positive |  |  |  |  |
| ≥5 | Levant | Positive |  |  |  |  |
| 0-2 | GCC |  |  | Positive |  |  |
| 0-2 | GCC |  |  | Positive |  |  |
| 2-5 | Asian | Positive |  |  |  |  |
| 0-2 | Levant |  |  | Positive |  |  |
| 2-5 | Africans | Positive |  |  |  |  |
| ≥5 | GCC | Positive |  |  |  |  |
| 0-2 | Asian | Positive |  |  |  |  |
| 0-2 | Levant |  |  | Positive |  |  |
| 2-5 | Other Arab | Positive |  |  |  |  |
| ≥5 | Asian | Positive |  |  |  |  |
| ≥5 | North Africans |  |  |  |  | Positive |
| 0-2 | GCC |  |  | Positive |  |  |
| 0-2 | Europeans | Positive |  |  |  |  |
| 0-2 | Other Arab |  |  | Positive |  |  |
| 0-2 | North Africans |  |  | Positive |  |  |
| 0-2 | Europeans | Positive |  |  |  |  |
| 0-2 | Levant | Positive |  |  |  |  |
| ≥5 | Asian | Positive |  |  |  |  |
| 2-5 | Levant | Positive |  | Negative |  |  |
| 0-2 | Levant |  |  | Positive |  |  |
| 0-2 | Levant |  |  | Positive |  |  |
| 2-5 | Asian | Positive |  |  |  |  |
| 0-2 | GCC |  |  | Positive |  |  |
| 0-2 | Other |  |  | Positive |  |  |
| 2-5 | GCC |  |  | Positive |  |  |
| 0-2 | Levant |  |  | Positive |  |  |
| 2-5 | Levant | Positive |  |  |  |  |
| 0-2 | Asian |  |  | Positive |  |  |
| 2-5 | Levant |  |  | Positive |  |  |
| ≥5 | Europeans | Positive |  |  |  |  |
| 0-2 | Levant |  |  | Positive |  |  |
| 0-2 | Asian | Positive |  |  |  |  |
| ≥5 | GCC |  |  | Positive |  |  |
| 0-2 | Asian |  | Positive |  |  |  |
| 2-5 | Levant | Positive |  | Negative |  |  |
| 0-2 | GCC |  | Positive |  |  |  |
| 0-2 | Europeans | Positive |  |  |  |  |
| ≥5 | Asian | Positive |  |  |  |  |
| ≥5 | Levant | Positive |  |  |  |  |
| 0-2 | Levant | Positive |  | Negative |  |  |
| 2-5 | North Africans |  |  | Positive |  |  |
| ≥5 | Other Arab |  | Positive |  |  |  |
| 2-5 | GCC | Positive |  |  |  |  |
| ≥5 | GCC | Positive |  |  |  |  |
| 0-2 | Other Arab | Positive | Negative |  |  |  |
| 0-2 | GCC |  | Positive |  |  |  |
| 0-2 | Levant | Positive |  | Negative |  |  |
| 0-2 | North Africans |  |  | Positive |  |  |
| 0-2 | North Africans |  |  | Positive |  |  |
| 0-2 | GCC |  |  | Positive |  |  |
| ≥5 | GCC | Negative | Positive |  |  |  |
| 2-5 | Europeans |  |  | Positive |  |  |
| 0-2 | Asian | Positive |  |  |  |  |
| 2-5 | GCC | Positive |  |  |  |  |
| ≥5 | GCC |  | Positive |  |  | Positive |
| 0-2 | Levant |  |  | Positive |  |  |
| 0-2 | Levant | Positive |  |  |  |  |
| ≥5 | GCC | Positive |  |  |  |  |
| 0-2 | GCC |  |  | Positive |  |  |
| 0-2 | GCC | Positive | Negative |  |  |  |
| 0-2 | GCC | Positive | Negative |  |  |  |
| 0-2 | GCC | Positive | Negative |  |  |  |
| 0-2 | Asian |  | Positive |  |  |  |
| 2-5 | North Africans |  |  |  |  | Positive |
| ≥5 | GCC | Positive |  |  |  |  |
| 0-2 | GCC | Positive | Negative |  |  |  |
| ≥5 | GCC | Positive | Negative |  |  |  |
| 0-2 | Levant |  |  | Positive |  |  |
| 0-2 | Other Arab |  |  |  |  | Positive |
| 0-2 | North Africans |  |  | Positive |  |  |
| 2-5 | GCC | Positive |  |  |  |  |
| 2-5 | GCC |  |  | Positive |  |  |
| 0-2 | North Africans |  |  | Positive |  |  |
| ≥5 | GCC | Positive |  |  |  |  |
| ≥5 | North Africans |  |  |  |  | Positive |
| ≥5 | GCC | Positive |  |  |  |  |
| 0-2 | GCC | Positive |  |  |  |  |
| 0-2 | Levant |  |  | Positive |  |  |
| ≥5 | GCC | Positive |  |  |  |  |
| 0-2 | GCC | Positive | Inconclusive |  |  |  |
| 0-2 | North Africans |  | Positive |  |  |  |
| ≥5 | GCC | Positive |  |  |  |  |
| ≥5 | GCC | Positive |  |  | Negative |  |
| ≥5 | GCC | Positive |  |  |  |  |
| 0-2 | GCC | Positive |  |  |  |  |
| ≥5 | Asian |  |  | Positive |  |  |
| 0-2 | Africans |  |  | Positive |  |  |
| 0-2 | GCC | Positive |  |  |  |  |
| 0-2 | Europeans | Positive |  |  |  |  |
| 0-2 | GCC |  |  | Positive |  |  |
| 2-5 | Europeans |  |  | Positive |  |  |
| 2-5 | GCC | Positive |  |  |  |  |
| ≥5 | GCC | Positive |  |  |  |  |
| 0-2 | GCC | Positive |  |  |  |  |
| 0-2 | GCC | Positive |  |  |  |  |
| ≥5 | GCC | Positive |  |  |  |  |
| ≥5 | GCC | Positive |  |  |  |  |
| 0-2 | GCC | Positive |  |  |  |  |
| 2-5 | GCC | Positive |  |  |  |  |
| 0-2 | Levant | Positive |  |  |  |  |
| 0-2 | GCC | Positive |  |  |  |  |
| 0-2 | Levant |  | Positive |  |  |  |
| 2-5 | GCC | Positive |  |  |  |  |
| ≥5 | GCC | Positive |  |  |  |  |
| ≥5 | GCC | Positive |  |  |  |  |
| ≥5 | GCC |  |  |  |  | Positive |
| ≥5 | Asian | Positive |  |  |  |  |
| 0-2 | GCC | Positive | Inconclusive |  |  |  |
| ≥5 | Europeans |  | Positive |  |  |  |
| 0-2 | GCC |  | Positive |  |  |  |
| 2-5 | GCC |  | Negative |  |  | Positive |
| ≥5 | GCC | Positive |  |  |  |  |
| 0-2 | GCC | Positive |  |  |  |  |
| ≥5 | GCC |  | Positive |  |  |  |
| 0-2 | GCC | Positive |  |  |  |  |
| 0-2 | GCC | Positive |  |  |  |  |
| 0-2 | GCC | Positive |  |  |  |  |
| ≥5 | Asian | Positive |  |  |  |  |
| 0-2 | North Africans | Positive |  |  |  |  |
| ≥5 | GCC | Positive |  |  |  |  |
| 0-2 | GCC |  | Positive |  |  |  |
| 0-2 | GCC | Positive |  |  |  |  |
| ≥5 | GCC | Positive |  |  |  |  |
| ≥5 | GCC | Positive |  |  |  |  |
| ≥5 | GCC | Positive |  |  |  |  |
| ≥5 | GCC | Positive |  |  |  |  |
| 0-2 | GCC |  | Positive |  |  |  |
| ≥5 | Levant | Positive |  |  |  |  |
| 0-2 | GCC |  | Positive |  |  |  |
| ≥5 | GCC |  | Positive |  |  |  |
| 0-2 | Europeans | Positive |  |  |  |  |
| 2-5 | GCC | Positive |  |  |  |  |
| 2-5 | North Africans | Positive |  |  |  |  |
| 0-2 | Africans | Negative | Positive |  |  |  |
| 0-2 | Levant | Positive |  |  |  |  |
| 2-5 | Other Arab |  |  |  |  | Positive |
| ≥5 | GCC | Positive |  |  | Negative |  |
| ≥5 | GCC | Positive |  |  |  |  |
| 0-2 | GCC |  | Positive |  |  |  |
| 2-5 | GCC | Positive |  |  |  |  |
| ≥5 | GCC | Positive |  |  |  |  |
| ≥5 | GCC | Positive |  |  |  |  |
| ≥5 | Asian | Positive |  |  |  |  |
| ≥5 | GCC | Positive |  |  |  |  |
| ≥5 | GCC | Positive |  |  |  |  |
| ≥5 | GCC | Positive |  |  |  |  |
| 0-2 | Levant |  | Positive |  |  |  |
| 0-2 | North Africans |  | Positive |  |  |  |
| 0-2 | GCC | Positive |  |  |  |  |
| 0-2 | GCC | Positive |  | Negative |  |  |
| ≥5 | GCC | Positive |  |  |  |  |
| 0-2 | Asian | Positive |  |  |  |  |
| ≥5 | Levant |  |  | Positive |  |  |
| 0-2 | Other Arab | Positive |  |  |  |  |
| 0-2 | GCC | Positive |  |  |  |  |
| 2-5 | GCC |  | Positive |  | Negative |  |
| 0-2 | GCC | Positive |  |  |  |  |
| 0-2 | GCC |  | Positive |  |  |  |
| 0-2 | GCC |  | Positive |  |  |  |
| 0-2 | GCC | Positive | Negative |  |  |  |
| 2-5 | GCC | Positive |  |  |  |  |
| 0-2 | Levant |  | Positive |  |  |  |
| ≥5 | Europeans | Positive |  |  |  |  |
| ≥5 | GCC | Positive |  |  |  |  |
| 0-2 | Asian |  | Positive |  |  |  |
| 2-5 | GCC | Positive |  |  |  |  |
| 2-5 | Asian | Positive |  |  |  |  |
| 2-5 | GCC | Positive |  |  |  |  |
| ≥5 | GCC | Positive |  |  |  |  |
| 0-2 | GCC |  | Positive |  |  |  |
| 0-2 | Levant | Positive |  |  |  |  |
| 2-5 | Levant | Positive |  |  |  |  |
| ≥5 | GCC | Positive |  |  |  |  |
| 0-2 | GCC |  | Positive |  |  |  |
| 0-2 | Other Arab |  | Positive |  |  |  |
| ≥5 | GCC | Positive |  |  |  |  |
| 2-5 | GCC | Positive |  |  |  |  |
| 0-2 | Other Arab | Positive |  |  |  |  |
| 2-5 | GCC | Positive |  |  |  |  |
| ≥5 | Asian | Positive |  |  |  |  |
| 0-2 | GCC | Positive |  |  |  |  |
| ≥5 | Europeans | Positive |  |  |  |  |
| 0-2 | GCC | Positive | Positive |  |  |  |
| ≥5 | GCC | Positive |  |  |  |  |
| 0-2 | GCC | Negative | Positive |  |  |  |
| 2-5 | Asian | Positive |  |  |  |  |
| ≥5 | Asian | Positive |  |  |  |  |
| 0-2 | GCC |  | Positive |  |  |  |
| ≥5 | Asian | Positive |  |  |  |  |
| 2-5 | GCC | Positive |  |  |  |  |
| 0-2 | GCC | Positive |  |  |  |  |
| ≥5 | GCC | Positive |  |  |  |  |
| 0-2 | GCC | Positive | Inconclusive |  |  |  |
| 0-2 | Other Arab | Positive |  |  |  |  |
| 2-5 | GCC | Positive |  |  |  |  |
| 2-5 | GCC | Positive |  |  |  |  |
| 0-2 | GCC | Positive |  |  |  |  |
| 0-2 | GCC | Positive |  |  |  |  |
| 2-5 | GCC |  | Positive |  |  |  |
| 0-2 | Asian | Positive |  |  |  |  |
| 0-2 | GCC | Positive |  | Negative |  |  |
| 0-2 | GCC |  | Positive |  |  |  |
| 2-5 | GCC | Positive |  |  |  |  |
| 2-5 | GCC | Positive |  |  |  |  |
| ≥5 | Asian | Positive |  |  |  |  |
| ≥5 | GCC | Positive |  |  |  |  |
| ≥5 | GCC | Positive |  |  |  |  |
| 0-2 | GCC | Positive |  |  |  |  |
| 0-2 | GCC |  | Positive |  |  |  |
| 0-2 | GCC | Positive | Negative |  |  |  |
| ≥5 | GCC |  | Positive |  |  |  |
| 0-2 | GCC | Positive | Negative |  |  |  |
| ≥5 | Asian | Positive |  |  |  |  |
| 0-2 | GCC |  | Negative |  |  | Positive |
| ≥5 | GCC | Positive |  |  |  |  |
| ≥5 | GCC |  | Positive |  |  |  |
| 0-2 | GCC | Positive |  |  |  |  |
| 0-2 | Levant |  |  | Positive |  |  |
| 2-5 | GCC |  | Positive |  | Negative |  |
| 0-2 | GCC |  |  | Positive |  |  |
| 0-2 | Levant | Positive |  |  |  |  |
| ≥5 | Asian | Positive |  |  |  |  |

| **Table S5. Disease Frequency** |  |  |
| --- | --- | --- |
| **locus/ gene** | **Syndrome/Disease** | **Disease frequency** |
| *ABCA4* | Stargardt disease / Retinitis pigmentosa | 1 |
| *ABCG2* | Susceptibility to hyperuricemia | 1 |
| *ABCG8* | Sitosterolemia | 1 |
| *ACO2* | Infantile cerebellar-retinal degeneration Isolated optic atrophy | 1 |
| *AGXT* | *Hyperoxaluria* | 1 |
| *AIRE* | Autoimmune polyendocrinopathy syndrome, type | 1 |
| *ALOX12B* | Congenital Ichthyosis | 1 |
| *ANO5* | ANO5 Muscle Disease | 1 |
| *ARID1B* | ARID1B-related disorder | 1 |
| arr[GRCh37] 10p15.3p14(116046_8337621)x1 | 10p15.3p14 contiguous deletion syndrome (HDR Syndrome) | 1 |
| arr[GRCh37] 11p13(31208677_31940343)x1 | Aniridia | 1 |
| arr[GRCh37] 11q24.2q25(126678645_134938470)x1 | Jacobsen Syndrome | 1 |
| arr[GRCh37] 12p13.33p11.1(173787_34835837)x3 | Trisomy 12p | 1 |
| arr[GRCh37] 12p13.33p11.1(173787_34835837)x3-4 | Pallister-Killian mosaic syndrome. | 1 |
| arr[GRCh37] 13q34(112059331_115107733)x1 | 13q34 terminal deletion | 1 |
| arr[GRCh37] 14q11.2q13.1(20,511,673_35,017,859)x1 | 14.5Mb 14q11.2q13.1 interstitial deletion | 1 |
| arr[GRCh37] 16p12.2(21946522_22431357)x4, 18p11.21(12818457_13097560)x4 | developmental delay and microcephaly, malar flattening, short palpebral fissures and abnormal nose. Clinical Features includes congenital heart disease, feeding difficulties, hypotonia, and mild motor delays. | 1 |
| arr[GRCh37] 16p13.11(15358445_16507781)x1 | 16p13.11 microdeletions | 1 |
| arr[GRCh37] 16q11.2q12.2(46503192_54771478)x1 | 16q11.2q12.2 microdeletion syndrome | 1 |
| arr[GRCh37] 17p12p11.2(15,754,174_20,552,548)x3 | Potocki-Lupski syndrome | 1 |
| arr[GRCh37] 17p13.3(1101215_2097377)x1 | postnatal growth retardation, cognitive impairment, facial dysmorphism, Chiari type 1 malformation and white matter abnormalities. | 1 |
| arr[GRCh37] 18q21.2q23(48935926_78014123)x3 | 29.1Mb 18q21.2q23 duplication | 1 |
| arr[GRCh37] 22q11.21(18,648,867_21,800,471)x3 | 22q11.2 duplication syndrome | 1 |
| arr[GRCh37] 22q13.31q13.33(46435927_51175776)x1 | Phelan-McDermid syndrome | 1 |
| arr[GRCh37] 2q11.1q11.2(96,732,520_98,249,638)x1 | 2q11.1q11.2 microdeletion syndrome | 1 |
| arr[GRCh37] 2q23.1(148971529_149068912)x1 | Mental retardation, autosomal dominant 1 | 1 |
| arr[GRCh37] 3p24.3p22.1(23810041_43429821)x3 | *19.6Mb duplication at 3p24.3p22.1* | 1 |
| arr[GRCh37] 4p13q11(43612839_52685687)x1, | *Developmental and epileptic encephalopathy 45* | 1 |
| arr[GRCh37] 4q31.1(140,294,194_140,401,566)x1 | 4q31.1 microdeletion | 1 |
| arr[GRCh37] 4q34.1q35.2(174744964_190957473)x1 | Craniofacial anomalies, musculoskeletal abnormalities, and intellectual disability with ocular, cardiac, genitourinary defects and pelvic/limb dysmorphism | 1 |
| arr[GRCh37] 4q34.3q35.2(183182681_190957473)x1 arr[GRCh37] 18q21.2q23(52890318_78014123)x3 | Two pathogenic copy number variants were identified on two separate chromosomes, which is suggestive of an unbalanced translocation | 1 |
| arr[GRCh37] 5p15.33p15.2(113,577_14,093,389)x1 | Cri-Du-chat syndrome | 1 |
| arr[GRCh37] 6q21(108,276,298_108,386,559)x0 | autosomal recessive osteopetrosis | 1 |
| arr[GRCh37] 6q24.1q24.2(141799351_145591148)x1 | Mental retardation, autosomal dominant 43 | 1 |
| arr[GRCh37] 7q11.21q36.3(62461704_159119220)x2 hmz | Full chromosome 7 LoH suggestive of uniparental isodisomy (UPD) and a diagnosis of Russell-Silver Syndrome | 1 |
| arr[GRCh37] 7q34q36.3(139,213,938_159,119,707)x3 | 7q microduplication syndrome | 1 |
| arr[GRCh37] Xp22.33q28(168547_155233731)x3 | TripleX Syndrome | 1 |
| arr[GRCh37] Xp22.33q28(535,235_155,233,731)x3 | 48,XXXY syndrome | 1 |
| arr[GRCh37]4q32.2q34.2(162,097,160_177,074,674)x1 | 14.98Mb Partial 4q32.2q34.2 deletion | 1 |
| *ARSA* | Metachromatic leukodystrophy | 1 |
| *ATM* | *Ataxia-telangiectasia* | 1 |
| *ATP1A3* | ATP1A3-related neurologic disorders | 1 |
| *ATP8B1* | ATP8B1 deficiency | 1 |
| *AVIL* | *Nephrotic syndrome* | 1 |
| *BBS10* | Bardet-Biedl Syndrome | 1 |
| *C4B* | C4B Deficiency | 1 |
| *CARMIL2* | Immunodeficiency 58 | 1 |
| *CASK* | CASK disorders | 1 |
| *CD36* | Platelet glycoprotein IV deficiency | 1 |
| *CD3D* | Immunodeficiency | 1 |
| *CD40LG* | X-Linked Hyper IgM Syndrome | 1 |
| *CDH3* | *Congenital hypotrichosis with juvenile macular dystrophy* | 1 |
| *CDKL5* | Developmental and epileptic encephalopathy | 1 |
| *CEL* | Maturity onset diabetes of the young | 1 |
| *CHRNE* | Myasthenic syndrome | 1 |
| *COL4A3* | Alport syndrome | 1 |
| *COL7A1* | Epidermolysis bullosa | 1 |
| *CPLANE1* | Joubert syndrome | 1 |
| *CPS1* | Carbamoylphosphate synthetase I deficiency | 1 |
| *CRB1* | CRB1 - associated retinopathies | 1 |
| *CTRC* | Susceptibility to chronic pancreatitis | 1 |
| *CYP21A2* | 21-Hydroxylase-Deficient Congenital Adrenal Hyperplasia | 1 |
| *DEPDC5* | DEPDC5-Related Epilepsy | 1 |
| *DMD* | Dystrophinopathy | 1 |
| *DNASE1L3* | *Hypocomplementemic urticarial vasculitis syndrome* | 1 |
| *DOCK6* | Adams-Oliver syndrome 2 | 1 |
| *DOCK8* | DOCK8 deficiency | 1 |
| *DUOX2* | *Thyroid dyshormonogenesis* | 1 |
| *EDA* | Ectodermal dysplasia | 1 |
| *EMC10* | Neurodevelopmental disorder with dysmorphic facies and variable seizures | 1 |
| *EXOSC9* | *Pontocerebellar hypoplasia type 1D* | 1 |
| *EXT1* | Hereditary multiple osteochondromas | 1 |
| *EYA1* | Branchiootorenal syndrome | 1 |
| *FAH* | Tyrosinemia | 1 |
| *FANCC* | Fanconi anemia | 1 |
| *FBXO22* | FBXO22 Associated Syndrome  * | 1 |
| *FGF12* | Early infantile epileptic encephalopathy | 1 |
| *FGFR3* | *Achondroplasia* | 1 |
| *FGFR3* | Hypochondroplasia | 1 |
| *FKBP10* | *Osteogenesis imperfecta type XI / Bruck syndrome* | 1 |
| *FKRP* | Muscular dystrophy-dystroglycanopathy | 1 |
| *FLNC* | Cardiomyopathy Myopathy | 1 |
| *FMR* | *Fragile X syndrome* | 1 |
| *FOXG1* | FOXG1-related disorder | 1 |
| *FOXP1* | *FOXP1-related neurodevelopmental disorder* | 1 |
| *FRRS1L* | Developmental and epileptic encephalopathy | 1 |
| *GAA* | GAA-related glycogen storage disease 2 | 1 |
| *GABRB2* | Developmental and epileptic encephalopathy | 1 |
| *GABRB3* | Developmental and epileptic encephalopathy | 1 |
| *GALT* | Galactosemia | 1 |
| *GATA3* | Hypoparathyroidism, sensorineural deafness, and renal dysplasia syndrome | 1 |
| *GHR* | Laron syndrome | 1 |
| *GLMN* | Hereditary glomuvenous malformations | 1 |
| *HIVEP2* | Intellectual developmental disorder 43 | 1 |
| *HSD11B2* | Apparent mineralocorticoid excess | 1 |
| *IC1 hypomethylation* | Russell-Silver Syndrome | 1 |
| *IGHMBP2* | Spinal muscular atrophy with respiratory distress /Charcot-Marie-Tooth disease | 1 |
| *IMPDH1* | Leber congenital amaurosis 11/ Retinitis pigmentosa 10 | 1 |
| *IQCB1* | Senior-Loken syndrome 5 | 1 |
| *JAG1* | *Alagille Syndrome* | 1 |
| *KLHL7* | PERCHING syndrome / Retinitis pigmentosa | 1 |
| *LAMA2* | Limb-girdle muscular dystrophy | 1 |
| *LAMB3* | Epidermolysis bullosa | 1 |
| *LMNA* | *LMNA-related disorders* | 1 |
| *LORICRIN* | Loricrin keratoderma | 1 |
| *LOXL3* | Myopia | 1 |
| *MCIDAS* | Primary ciliary dyskinesia | 1 |
| *METTL23* | Intellectual disability 44 | 1 |
| *MMACHC* | Methylmalonic aciduria and homocystinuria, cblC type | 1 |
| *MMUT* | Methylmalonic aciduria | 1 |
| *MPV17* | Mitochondrial DNA depletion syndrome | 1 |
| *MVK* | Hyper-IgD periodic fever syndrome | 1 |
| *MYO5B* | Microvillus inclusion disease | 1 |
| *NANS* | Infantile-onset developmental delay & skeletal dysplasia | 1 |
| *NCF1* | Chronic granulomatous disease | 1 |
| *NCKAP1L* | *Immunodeficiency with autoinflammation* | 1 |
| *NEB* | Nemaline myopathy | 1 |
| *NKX2-1* | NKX2-1 Related Disorders | 1 |
| *NPHP1* | Joubert Syndrome | 1 |
| *NPHS1* | Nephrotic syndrome type 1 | 1 |
| *NPR2* | NPR2-related disorders | 1 |
| *NRL* | Enhanced S-cone syndrome | 1 |
| *NSD1* | Sotos syndrome | 1 |
| *OFD1* | OFD1-related ciliopathy | 1 |
| *OPA1* | Optic atrophy | 1 |
| *PACS2* | Developmental and epileptic encephalopathy | 1 |
| *PAH* | Phenylalanine Hydroxylase Deficiency | 1 |
| *PCDH19* | PCDH19-related epilepsy | 1 |
| *PDHA1* | Pyruvate dehydrogenase E1-alpha deficiency | 1 |
| *PDZD7* | Deafness | 1 |
| *PJVK* | Deafness | 1 |
| *PLCE1* | Nephrotic syndrome, type 3 | 1 |
| *PNPO* | Pyridoxamine 5-prime-phosphate oxidase deficiency | 1 |
| *POLD1* | POLD1-related disorders; lipodystrophy | 1 |
| *POLG* | POLG-related disorder Progressive external ophthalmoplegia | 1 |
| *POMT1* | Muscular dystrophy-dystroglycanopathy | 1 |
| *PRRT2* | PRRT2-Associated Paroxysmal Movement Disorders | 1 |
| *PRSS1* | PRSS1-Related Hereditary Pancreatitis | 1 |
| *PTPN11* | Noonan syndrome / Noonan syndrome with multiple lentigines / metachondromatosis | 1 |
| *PTRH2* | Infantile-onset multisystem neurologic, endocrine, and pancreatic disease | 1 |
| *RDH12* | Retinal dystrophy | 1 |
| *RIT1* | Noonan syndrome | 1 |
| *RNF13* | Developmental and epileptic encephalopathy | 1 |
| *RPGRIP1* | Leber congenital amaurosis | 1 |
| *RS1* | Retinoschisis | 1 |
| *RUNX2* | Cleidocranial dysplasia spectrum disorder | 1 |
| *RYR1* | Central core disease / {susceptibility to malignant hyperthermia} | 1 |
| *SAMD9* | MIRAGE syndrome | 1 |
| *SBF1* | Charcot-Marie-Tooth disease | 1 |
| *SCN4A* |  | 1 |
| *SCN8A* | SCN8A-Related Epilepsy with Encephalopathy | 1 |
| *SCNN1A* | Pseudohypoaldosteronism type I | 1 |
| *SFTPB* | Pulmonary surfactant metabolism dysfunction | 1 |
| *SGCB* | Limb-girdle muscular dystrophy | 1 |
| *SHOC2* | Noonan syndrome-like with loose anagen hair | 1 |
| *SIGMAR1* | Distal motor neuropathy | 1 |
| *SLC26A3* | Congenital secretory chloride diarrhea / intestinal obstruction | 1 |
| *SLC3A1* | Cystinuria | 1 |
| *SLC5A1* | Glucose – galactose malabsorption | 1 |
| *SLC5A7* | Distal hereditary motor neuronopathy / congenital myasthenic syndrome | 1 |
| *SMC1A* | SMC1A-related disorder | 1 |
| *SNRPN/ 15q11* | Maternal 15q duplication syndrome | 1 |
| *SNX10* | Osteopetrosis | 1 |
| *SPAST* | Spastic paraplegia 4 | 1 |
| *SPINK5* | Netherton syndrome | 1 |
| *SPINT2* | Congenital syndromic sodium secretory diarrhea / congenital tufting enteropathy | 1 |
| *SPTAN1* | Epileptic encephalopathy, early infantile, 5 | 1 |
| *SPTB* | Spherocytosis | 1 |
| *STXBP1* | STXBP1 related Encephalopathy with Epilepsy | 1 |
| *SVBP* | Neurodevelopmental disorder with ataxia, hypotonia, and microcephaly | 1 |
| *SYN1* | Epilepsy | 1 |
| *TANGO2* | TANGO2-Related Metabolic Encephalopathy and Arrhythmias | 1 |
| *TBL1XR1* | Autosomal dominant intellectual disability / Pierpont syndrome | 1 |
| *TCN2* | Transcobalamin II deficiency | 1 |
| *TMC1* | Nonsyndromic Hearing loss | 1 |
| *TMEM67* | Joubert syndrome | 1 |
| *TNFAIP3* | Autoinflammatory syndrome, familial, Behcet-like | 1 |
| *TRAPPC12* | Early-onset progressive encephalopathy with brain atrophy and spasticity | 1 |
| *TRPM1* | Congenital stationary night blindness | 1 |
| *UNC13D* | Hemophagocytic lymphohistiocytosis, familial, 3 | 1 |
| *USP7* | Hao-Fountain syndrome | 1 |
| *VWF* | Von Willebrand Disease | 1 |
| *WAS* | WAS-related disorders | 1 |
| *WDR45* | Beta-Propeller Protein-Associated Neurodegeneration | 1 |
| *ALDH7A1* | Pyridoxine-dependent Epilepsy | 2 |
| arr[GRCh37] 13q11q34(19,436,287_115,038,009)x3 | Patau Syndrome Trisomy 13 | 2 |
| arr[GRCh37] 15q11.2(22,770,422_23,688,962)x1 | 15q11.2 microdeletion syndrome | 2 |
| arr[GRCh37] 18p11.32q23(136,227_78,014,123)x3 | Edwards Syndrome Trisomy 18 | 2 |
| arr[GRCh37] 22q11.21(18916843_21465662)x1 | DiGeorge Syndrome | 2 |
| arr[GRCh37] 8p23.3p23.2(158,049_2,411,410)x1 arr[GRCh37] 8p23.2p22(2,412,280_17,131,869)x3 arr[GRCh37] 8p23.1(8175258_11858261)x1 | 8p inverted duplication/deletion syndrome and/or 8p23.1 deletion syndrome | 2 |
| *BTD* | Biotinidase Deficiency | 2 |
| *CCNO* | Primary ciliary dyskinesia | 2 |
| *CDKN1C  KCNQ1OT1/H19* 11p15 | Beckwith-Wiedemann syndrome | 2 |
| *CFHR3/CFHR1* | atypical hemolytic-uremic syndrome (aHUS) | 2 |
| *CHD7* | CHARGE syndrome | 2 |
| *CYP1B1* | Primary Congenital Glaucoma | 2 |
| *DGAT1* | Congenital diarrhoeal disorder | 2 |
| *GJB2* | Deafness | 2 |
| *KCNQ2* | KCNQ2-Related Disorders | 2 |
| *NLRP12* | Familial cold autoinflammatory syndrome Common variable immunodeficiency | 2 |
| *PKHD1* | Polycystic kidney disease | 2 |
| *TSC2* | Tuberous sclerosis 2 | 2 |
| *UGT1A1* | Crigler-Najjar Syndrome/Gilbert Syndrome | 2 |
| arr[GRCh37] 21q11.2q22.3(15006458_48097372)x3 | Down Syndrome Trisomy 21 | 3 |
| *FBN1* | Marfan syndrome/ FBN1-associated disorders | 3 |
| *G6PD* | G6PD deficiency | 3 |
| arr[GRCh37] 7q11.23(72,643,632_74,142,190)x1 | Williams-Beuren Syndrome | 4 |
| *CFTR* | Cystic Fibrosis / Congenital Absence of the Vas Deferens | 4 |
| *DMD* | Duchenne muscular dystrophy | 4 |
| *HBB* | Sickle cell anemia / abnormal hemoglobin types Β- Thalassemia | 4 |
| *NF1* | Neurofibromatosis type 1 | 4 |
| *SLC26A4* | Pendred syndrome / Deafness with enlarged vestibular aqueduct | 4 |
| arr[GRCh37] Xp22.33p11.21(168547_55476636)x1, Xp11.21q28(55548946_155233731)x3 | Turner Syndrome | 5 |
| arr[GRCh37] Xp22.33q21.33(168547_96275443)x1-2, Xq22.1q28(98849076_155233731)x1 | Turner Syndrome |  |
| arr[GRCh37] Xp22.33q28(168,547_155,233,731)x1 | Turner Syndrome |  |
| arr[GRCh37] Xp22.33q28(168,567_155,233,731)x1 | Turner Syndrome |  |
| arr[GRCh37] Xp22.33q28(168547_155233731)x2-3 | Mosaic chromosome X duplication/ Mosiac Turner Syndrome |  |
| *IFIH1* | Aicardi-Goutieres syndrome / Singleton-Merten Syndrome | 6 |
| *MEFV* | Familial Mediterranean Fever | 6 |
| *SNRPN/ 15q11* | Prader Willi/ Angelman syndrome | 9 |
| *SMN1* | Spinal Muscular Atrophy | 51 |

| **Table S6. Clinically significant sequence variants in patients with positive findings (N = 211)** | | | | | | | | | | |  |
| --- | --- | --- | --- | --- | --- | --- | --- | --- | --- | --- | --- |
| **Mode of Inheritance** | | **Type of NGS testing** | **Variants (cDNA; protein)** | **ACMG-AMP codes** | **Genomic Coordinates** | **Novel or** | **Zygosity** | **Variant Origin** | **Syndrome/Disease** | **Management and Intervention** |  |
|  |  |  |  |  |  | **Reported** |  |  |  |  |  |
| AR/AD | | Indication based Exome | *SCN4A* (NM_000334.4): c.4382T>G; (p.Leu1461Arg) | PM2, PM1, PM5, PP3, PP4 | 17:62019260 | Novel | Het. | NA | Muscle stiffness |  |  |
| AR/AD | | Indication based Exome | *MEFV* (NM_000243.2): c.2040G>C; p.(Met680Ile) | PS4, PM3_Strong, PP4, PP1 | 16:3293447 | Reported | Hom. | NA | Familial Mediterranean Fever | Started the patient on Colchicine |  |
| AD | | Indication based Exome | *SAMD9* (NM_017654.3): c.3877C>T; p.(Arg1293Trp) | PS2, PS3, PM5, PM2, PP4, PP3 | 7:92731534 | Reported | Het. | NA | MIRAGE syndrome | Adrenal replacement therapy, initiation of antibiotic prophylaxis. |  |
| AR | | Whole exome Trio | *MYO5B* (NM_001080467.2): c.2062C>T; p.(Arg688*) | PVS1, PM2, PP4 | 18:47455910 | Reported | Hom. | Mother & Father | Microvillus inclusion disease | Enrolled in Phase II study (Shylicine^TM^) for microvillus inclusion disease by Vanessa Research Hungary LTD. Shylicine^TM^ works by increasing absorptive capacity of intestinal epithelium. |  |
| AR | | Indication based Exome | *UGT1A1* (NM_000463.2): c.1021C>T; p.(Arg341*) | PVS1, PM3_Strong, PM2, PS3 | 2:234676519 | Reported | Hom. | NA | Crigler-Najjar Syndrome/Gilbert Syndrome | Avoidance of unnecessary testing for further episodes of jaundice. |  |
| AR | | Indication based Exome | *SGCB* (NM_000232.4): c.622-1G>C; p? | PVS1, PM2, PP4 | 4:52894266 | Reported | Hom | NA | Limb-girdle muscular dystrophy | Cardiology evaluation and follow up for cardiomyopathy |  |
| AD | | Indication based Exome | *SPTAN1* (NM_001130438.2): c.6908_6916dup; p.(Asp2303_Leu2305dup) | PS2, PS4, PM1, PS3, PP4 | 9:131394540 | Reported | Het. | NA | Epileptic encephalopathy, early infantile, 5 |  |  |
| AR | | Indication based Exome | (*CFHR3*/*CFHR1* homozygous deletion) arr[GRCh37] 1q31(19674397_196801319)x1 | PVS1, PP4, PM3_Strong | 1:196743970-196801319 | Reported | Hom | NA | atypical hemolytic-uremic syndrome (aHUS) | Started the patient on Eculizumab |  |
| AD | | Whole exome Trio | *CHD7* (NM_017780.3): c.4120_4121dupAA; p.(Asn1374Lysfs*31) | PVS1, PS2, PM2, PP4 | 8:61749505 | Novel | Het. | De Novo | CHARGE syndrome | (1) Abdominal ultrasound for renal lesions, (2) Brain MRI for cranial nerves defects |  |
| AD | | Indication based Exome | *JAG1* (NM_000214.2): c.1052delG; p.(Cys351Leufs*61) | PVS1, PM2, PP4 | 20:10632296 | Novel | Het. | NA | Alagille Syndrome | (1) Annual Alpha feta protein and U/S (for liver cancer) (2) Referral to Nephrology and Neurology |  |
| AD | | Indication based Exome | *CHD7* (NM_017780.3): c.5677G>T; p.(Glu1893Ter) | PVS1, PM2, PP4 | 8:61764589 | Novel | Het. | NA | CHARGE syndrome | (1) Abdominal ultrasound for renal lesions, (2) Brain MRI for cranial nerves defects |  |
| AR | | Indication based Exome | *C4B* NM_001002029.3:c.(?_3231)_(3387_?)del | PVS1, PM2, PP4 | 6:31996470-31996626 | REPORTED | Hom. | NA | *C4B* Deficiency |  |  |
| AR | | Indication based Exome | *CFTR* (NM_000492.3): c.1210-12T[5]; p.? | PS4, PS3, PP4 | 7:117188682 | Novel | Hom | NA | Cystic Fibrosis / Congenital Absence of the Vas Deferens | Multidisciplinary follow up with pulmonology, Gastroenterology, general paediatrics, and dietician. |  |
| AR | |  | CFTR (NM_000492.3): c.3208C>T; p.(Arg1070Trp) | PM3_VeryStrong, PM2, PS3 | 7:117251703 | Reported | Hom | NA | Cystic Fibrosis / Congenital Absence of the Vas Deferens |  |  |
| AD | | Indication based Exome | *IFIH1* (NM_022168.3): c.769+3A>G; p.? | PM2, PP3, PP4 | 2:163163216 | Reported | Het. | NA | Aicardi-Goutieres syndrome / Singleton-Merten syndrome |  |  |
| AR | | Whole exome Trio | *EXOSC9* (NM_001034194.1): c.41T>C; p.(Leu14Pro) | PM3_Strong, PS3, PP4 | 4:122722620 | Novel | Hom | Mother & Father | Pontocerebellar hypoplasia type 1D |  |  |
|  |  |  |  |  |  |  |  |  |  |  |  |
| AR | | Whole exome Trio | *NANS (*NM_018946.3): c.452G>A; p.(Arg151His) | PM3, PM2, PP4, PP3 | 9:100840478 | Reported | Hom. | Mother & Father | Infantile-onset developmental delay & skeletal dysplasia |  |  |
| AD | | Indication based Exome | *PRRT2* (NM_145239.2): c.649dupC; p.(Arg217Profs*8) | PVS1, PS4, PP4, PM2 | 16:29825016 | Reported | Hom* | NA | PRRT2-Associated Paroxysmal Movement Disorders |  |  |
| AR/AD | | Indication based Exome | *NPR2* (NM_003995.3): c.2326C>T; p.(Arg776Trp) | PM3, PM2, PP3, PS3 | 9:35806184 | Reported | Hom | NA | *NPR2*-related disorders |  |  |
| AR | | Whole exome Trio | *RPGRIP1* (NM_020366.3): c.1107del; p.(Glu370Asnfs*5) | PVS1, PM2, PP4 | 14:21780617 | Reported | Hom | Mother & Father | Leber congenital amaurosis |  |  |
| AR | | Indication based Exome | *NPHS1* (NM_004646.3): c.2071+1G>T; p.? | PVS1, PM2 | 19:36335220 | Reported | Hom | NA | Nephrotic syndrome type 1 | (1) Clarification of aetiology of nephrotic syndrome in view of congenital CMV infection. (2) Avoidance of immunosuppression (3) Planned nephrectomy and future kidney transplantation |  |
| AD | | Indication based Exome | *RIT1* (NM_006912.5): c.246T>G; p.(Phe82Leu) | PS2_VeryStrong, PP4, PM2 | 1:155874285 | Reported | Het | NA | Noonan syndrome | Growth Hormone |  |
| AR/AD | | Indication based Exome | *COL7A1* (NM_000094.3): c.6734G>A; p.(Gly2245Asp) | PM2, PM5, PM1, PP4 | 3:48610470 | Novel | Het | NA | Epidermolysis bullosa | (1) Surveillance for squamous cell carcinoma (2) screening for anemia and deficiencies of iron, zinc, vitamin D, selenium, and carnitine every 6-12 months (3) Yearly echocardiograms to identify dilated cardiomyopathy |  |
| AD | | Whole exome Trio | *TBL1XR1 (*NM_024665.5): c.226C>T; p.(Arg76*) | PVS1, PM6, PM2, PP4 | 3:176769493 | Reported | Het | De Novo | Autosomal dominant intellectual disability / Pierpont syndrome | Speech monitoring |  |
| AR | | Indication based Exome | *ATP8B1* (NM_005603.4): c.3040C>T; p.(Arg1014*) | PVS1, PM2, PP4 | 18:55319937 | Reported | Hom | NA | ATP8B1 deficiency |  |  |
| AR | | Whole exome Trio | *CRB1* (NM_201253.2): c.997G>C; p.(Gly333Arg) | PM2, PM5, PP4, PP3 | 1:19732596 | Reported | Cmpd Het. | Father | *CRB1* - associated retinopathies |  |  |
|  |  |  | *CRB1* (NM_201253.2): c.2573A>G; p.(Asn858Ser) | PM2, PM5, PP4, PP3, PM3 | 1:197397028 | Novel |  | Mother |  |  |  |
| AD | | Indication based Exome | *RUNX2* (NM_001024630.3): c.568C>T; p.(Arg190Trp) | PS4, PP1, PM6, PM5, PM1, PM2, PP3 | 6:45399744 | Reported | Het | NA | Cleidocranial dysplasia spectrum disorder |  |  |
| AD | | Whole exome Trio | *ATP1A3* (NM_152296.4): c.2267G>A; p.(Arg756His) | PS4, PS2, PM1, PM5, PP3, PM2, PP3 | 19:42474691 | Reported | Het. | De Novo | *ATP1A3*-related neurologic disorder (Fever-Induced Paroxysmal Weakness and Encephalopathy) | *(1) Aggressive treatment of fever episodes and immediate physical therapy after hypotonic episodes. (2) Oxcarbazepine (modest response)* |  |
| X-Linked Dominant | |  | *G6PD* (NM_001042351.3): c.563C>T; p.(Ser188Phe) | PS4, PS3, PP4 | X:153762634 | Reported | Hemi | Mother | *G6PD* deficiency | *Avoidance of foods and drugs that can lead to haemolysis.* |  |
| AD | | Indication based Exome | *IFIH1* (NM_022168.3): c.1764del; p.(Ala589Leufs*16) | PVS1, PM2 | 2:163134716 | Reported | Het. | NA | Aicardi-Goutières syndrome / Singleton Merten syndrome |  |  |
| AD | | Indication based Exome | *IFIH1* (NM_022168.3): c.1641+1G>C; p.? | PVS1, PS3 | 2:163136505 | Reported | Het. | NA | Aicardi-Goutières syndrome / Singleton Merten syndrome |  |  |
| AR | | Whole exome Trio | *DUOX2* (NM_014080.4): c.1709A>T; p.(Gln570Leu) | PM3, PS3, PP3 | 15:45399152 | Reported | Cmpd Het. | Mother | Thyroid |  |  |
|  |  |  | *DUOX2* (NM_014080.4): c.144C>A; p.(His48Gln) | PM2, PM3, PP4 | 15:45405201 | Reported |  | Father | dyshormonogenesis |  |  |
| AR/AD | | Indication based Exome followed by whole exome proband re-analysis | *MEFV* (NM_000243.2): c.2040G>A p.(Met680Ile) | PS4, PM3_Strong, PP4, PP1 | 16:3293447 | Reported | Het. | NA | Familial Mediterranean Fever | Started the patient on Colchicine |  |
| AD | |  | *PRSS1* (NM_002769.4): c.365G>A; p.(Arg122His) | PS4, PS3, PM2, PP4 | 7:142459789 | Reported | Het | NA | PRSS1-Related Hereditary Pancreatitis |  |  |
| AD | | Whole exome Proband | *CEL* (NM_001807.4): c.346C>T; p.(Gln116*) | PVS1, PM2 | 9:135940146 | Novel | Het | NA | Maturity onset diabetes of the young | Appropriate selection of anti-diabetic medication |  |
| AD | |  | *KLHL7* (NM_001031710.2): c.1229G>A; p.(Trp410*) | PVS1, PM2 | 7:23207506 | Novel | Het | NA | PERCHING syndrome / Retinitis pigmentosa | Referral to Ophthalmology |  |
| AR | | Expedited Whole Exome Quad | *CD36* (NM_001001547.2): c.(?_-183)_(120_?)del | PVS1, PM2, PP4 | 7:80275404-80276176 | Novel | Hom | NA | Platelet glycoprotein IV deficiency |  |  |
| AD | | Indication based Exome | *FGFR3* (NM_000142.4): c.1138G>A; p.(Gly380Arg) | PS4, PP4, PP3, PM5, PM2 | 4:1806119 | Reported | Het | NA | Achondroplasia | (1) MRI brain and spine (2) Cardiology follow up |  |
| AD | | Indication based Exome | *TNFAIP3* (NM_001270508.1): c.1939A>C; p.(Thr647Pro) | PS3, PP3, PP4 | 6:138201240 | Novel | Het. | NA | Autoinflammatory syndrome, familial, Behcet-like | Started the patient on Colchicine |  |
| AD | | Whole exome Trio | *IFIH1* (NM_022168.3): c.2016del; p.(Asp673Ilefs*5) | PVS1, PM2, PP4 | 2:163133953 | Reported | Het | Father | Aicardi-Goutieres syndrome / Singleton-Merten Syndrome |  |  |
| AD | |  | *MYBPC3* (NM_000256.3): c.2148+1G>A p.?  **(Secondary Finding)** | PVS1, PM5, PM2 | 11:47360874 | Novel | Het | Father | Familial hypertrophic cardiomyopathy /Dilated cardiomyopathy | Cardiology follow up. |  |
| AR/AD | | Indication based Exome | *MEFV* (NM_000243.2): c.2177T>C; p.(Val726Ala) | PS4, PM3_VeryStrong, PS3 | 16:3293310 | Reported | Hom. | NA | Familial Mediterranean Fever | Started the patient on Colchicine |  |
| AD | | Whole exome Trio | *NLRP12* (NM_144687.3): c.3046C>T; p.(Arg1016*) | PVS1 | 19:54299165 | Reported | Het | Mother | Familial cold autoinflammatory syndrome Common variable immunodeficiency |  |  |
| AR | | Indication based Exome followed by whole exome proband re-analysis | *SLC26A3* (NM_000111.2): c.559G>T; p.(Gly187*) | PVS1, PM3_VeryStrong, PM2 | 7:107431504 | Reported | Hom | NA | Congenital secretory chloride diarrhea / intestinal obstruction |  |  |
| AD | |  | *IFIH1* (NM_022168.4): c.2016del; p.(Asp673Ilefs*5) | PVS1 | 2:163133953 | Reported | Het | NA | Aicardi-Goutieres syndrome / Singleton-Merten Syndrome |  |  |
| AR/AD | | Whole exome Proband | *MEFV* (NM_000243.2): c.2177T>C; p.(Val726Ala) | PS4, PM3_VeryStrong, PS3 | 16:3293310 | Reported | Cmpd Het. | NA | Familial Mediterranean Fever | Started the patient on Colchicine |  |
|  |  |  | *MEFV* (NM_000243.2): c.442G>C; p.(Glu148Gln) | Risk Allele | 16:3304626 | Reported |  |  |  |  |  |
| AD | | Indication based Exome | *EYA1* (NM_000503.5): c.1051-5T>G; p.? | PP1_Moderate, PM2, PP3, PP4 | 8:72156932 | Novel | Het | NA | Branchiootorenal syndrome |  |  |
| AD | | Whole exome Trio | *RYR1* (NM_000540.2): c.14581C>T; p.(Arg4861Cys) | PS2_VeryStrong, PP4, PM2, PM5, PM1 | 19:39071079 | Reported | Het | De Novo | Central core disease / {susceptibility to malignant hyperthermia} | Precaution with General anaesthesia. |  |
| AR | | Indication based Exome | *PKHD1* (NM_138694.3): c.107C>T: p.(Thr36Met) | PS4,PM2,PM3,PP3,PP5 | 6:51947999 | Reported | Cmpd Het. | Mother | Polycystic kidney disease |  |  |
|  |  |  | *PKHD1* (NM_138694.3):c.5134G>A:p.(Gly1712Arg) | PM2,PP3 | 6:51889474 | Reported |  | Father |  |  |  |
| AR | | Indication based Exome | *FANCC* (NM_000136.2): c.165+1G>T; p.? | PVS1, PM3, PS3 | 9:98011408 | Reported | Hom | NA | Fanconi anemia | Bone marrow transplant. |  |
| AD | | Expedited Whole Exome Trio | *NLRP12* (NM_144687.3): c.1952C>A; p.(Ser651*) | PVS1, PM2 | 19:54312961 | Reported | Het. | Mother | Familial cold autoinflammatory syndrome |  |  |
| AR | | Indication based Exome | *SBF1* (NM_002972.3): c.5463C>G; p.(Tyr1821*) | PVS1, PM2 | 22:50885871 | Novel | Hom | NA | Charcot-Marie-Tooth disease |  |  |
| AR | | Whole Exome Trio | *DGAT1* (NM_012079.5): c.1374G>A; p.(Trp458*) | PVS1, PM3, PM2 | 8:145540310 | Reported | Hom | Mother | Congenital diarrhoeal disorder | Suggesting bowel transplant |  |
| X-Linked Recessive | | Indication based Exome | *RS1* (NM_000330.3): c.52+3A>G; p.? | PS4, PM2, PP3, PP4 | X:18690134 | Reported | Hemi | NA | Retinoschisis | Follow up with ophthalmologist specialized in vitreoretinal diseases. Correct diagnosis confirmed (formally misdiagnosed) |  |
| AR | | Indication based Exome | *CYP1B1* (NM_000104.3): c.917G>A; p.(Gly306Glu) | PM3 | 2:38301615 | Reported | Cmpd Het. | NA | Primary Congenital Glaucoma |  |  |
| AR | |  | *CYP1B1* (NM_000104.3): c.1103G>A; p.(Arg368His) | PM3_Strong, PS3 | 2:38298394 | Reported |  |  |  |  |  |
| X-linked Recessive | | Indication based Exome | *DMD* (NM_004006.2): c.(?_6439)_ (8217_?) | PVS1, PM2, PP4, PM3 | X:31986631-31645791 | REPORTED | Hemi | NA | Dystrophinopathy | (1) long term steroids, (2) consideration for emerging gene-based therapies (e.g. exon skipping) (3) Cardiology referral |  |
| AR | | Indication based Exome | *NRL* (NM_006177.4): c.339C>G; p.(Tyr113*) | PVS1, PM3, PM2 | 14:24551719 | Reported | Hom | NA | Enhanced S-cone syndrome |  |  |
| AD (reduced penetrance) | | Whole exome Trio | *OPA1* (NM_015560.2): c.2708_2711del; p.(Val903Glyfs*3) | PVS1, PM3_Strong, PS3, PM2 | 3:193384957 | Reported | Het | Father | Optic atrophy |  |  |
| AD | | Indication based Exome | arr[GRCh37] 11p13(31208677_31940343)x1 | PVS1, PM2, PP4 | 11:31208677- 31940343 | REPORTED | Het | NA | Aniridia |  |  |
| AR | | Indication based Exome | *ALOX12B* (NM_001139.2): c.944T>C; p.(Leu315Pro) | PM3, PM2, PP3, PP4 | 17:7982841 | Reported | Hom | NA | Congenital Ichthyosis |  |  |
| X-linked Recessive / X-linked Dominant | | Indication based Exome | *SYN1* (NM_133499.2): c.1166dup; p.(Ser390Phefs*8) | PVS1, PM2, PP4 | X:47434665 | Novel | Hemi. | NA | Epilepsy |  |  |
| AR | | Whole exome Trio | *SVBP* (NM_199342.4): c.82C>T; p.(Gln28*) | PVS1, PM3, PP1, PM2, PP4 | 1:43282134 | Reported | Hom | Mother and Father | Neurodevelopmental disorder with ataxia, hypotonia, and microcephaly |  |  |
| AD | | Indication based Exome | *FGF12* (NM_021032.4): c.341G>A; p.(Arg114His) | PS2_VeryStrong, PM2, PP4, PS3 | 3:192053223 | Reported | Het. | NA | Early infantile epileptic encephalopathy 47 | Sodium channel blocking antiepileptic agents (Lacosamide, Oxcarbazepine and Phenytoin) with relatively good response. |  |
| AD | | Whole exome Trio | *POLD1(*NM_002691.4): c.3199G>A; p.(Glu1067Lys) | PP1, PM2, PM1, PS2, PP4 | 19:50920507 | Reported | Het | De Novo | *POLD1*-related disorders; lipodystrophy | *Ezetrol/atorvastatin, Pioglitazone, Cholecalciferol, Metformin, Iodine supplements and Beta HCG* |  |
| AR | | Indication based Exome | *TRPM1* (NM_002420.5): c.1197G>A; p.(Pro399=) | PM3, PM2, PP3, PP4 | 15:31352747 | Reported | Hom | NA | Congenital stationary night blindness |  |  |
| AR | | Indication based Exome | *SLC26A4* (NM_000441.1): c.706C>G; p.(Leu236Val) | PM2_Supporting, PM3_Strong, PM5, PP1, PP4 | 7:107315495 | Reported | Cmpd Het. | NA | Deafness with enlarged vestibular aqueduct / Pendred syndrome | Underwent Bilateral Cochlear implantation |  |
|  |  |  | *SLC26A4* (NM_000441.1): c.1963A>G; p.(Ile655Val) | PM2, PP4, PM3, PP1 | 7:107342431 | Reported |  |  |  |  |  |
| AR | | Indication based Exome | *FKRP (*NM_024301.5): c.649C>A; p.(Pro217Thr) | PM3, PP4, PS3_Moderate, PM2, PP3 | 19:47259356 | Reported | Hom | NA | Muscular dystrophy-dystroglycanopathy |  |  |
| AD | | Indication based Exome | *NF1* (NM_000267.3): c.499_502del; p.(Cys167Glnfs*10) | PVS1, PS4, PM2 | 17:29496924 | Reported | Het | NA | Neurofibromatosis | Follow American academy of paediatrics guidelines. Health Supervision for Children with Neurofibromatosis Type 1 |  |
| AR | | Indication based Exome | *CCNO* (NM_021147.4): c.259_268dup; p.(Val90Glyfs*49) | PVS1, PM2, PP4 | 5:54529084 | Reported | Hom | NA | Primary ciliary dyskinesia | Pneumococcal vaccine |  |
| AR | | Indication based Exome | *LAMB3 (*NM_000228.2): c.3247C>T; p.(Gln1083*) | PVS1, PM2, PP4 | 1:209789951 | Reported | Hom | Biparental | Epidermolysis bullosa | (1) Surveillance for squamous cell carcinoma (2) screening for anemia and deficiencies of iron, zinc, vitamin D, selenium, and carnitine every 6-12 months (3) Yearly echocardiograms to identify dilated cardiomyopathy |  |
| AR | | Whole exome Trio | *FBXO22* (NM_147188.2): c.159_162del; p.(Arg53Serfs*13) | PVS1, PM3_VeryStrong, PS3, PM2 | 15:76196845 | Novel | Hom | Mother and Father | ? |  |  |
| AD | | Whole exome Trio | *ABCG2* (NM_004827.2): c.791_792del; p.(Leu264Hisfs*14) | PVS1, PM2 | 4:89039310 | Reported | Het | Mother | Susceptibility to hyperuricemia |  |  |
| AR | | Indication based Exome | *GJB2* (NM_004004.5): c.313_326del; p.(Lys105Glyfs*5) | PVS1, PM3_VeryStrong | 13:20763395 | Reported | Cmpd Het. | NA | Deafness |  |  |
|  |  |  | *GJB2* (NM_004004.5): c.235del; p.(Leu79Cysfs*3) | PVS1, PM3_VeryStrong, PS3_Moderate | 13:20763486 | Reported |  |  |  |  |  |
| AD | | Whole exome Trio | *SHOC2* (NM_007373.3): c.4A>G; p.(Ser2Gly) | PS2, PM2, PS3 | 10:112724120 | Reported | Het | De Novo | Noonan syndrome-like with loose anagen hair |  |  |
| AR | | Indication based Exome | *DOCK8* (NM_203447.3): c.4241+1G>A; p.? | PVS1, PM2, PP4 | 9:422136 | Novel | Hom | NA | *DOCK8* deficiency | *Bone Marrow Transplant* |  |
| AR | | Whole exome Trio | *ARSA* (NM_000487.6): c.449C>T; p.(Pro150Leu) | PM3, PP2, PM2, PM5, PS3_Moderate, PP3 | 22:51065610 | Reported | Hom | Mother and Father | Metachromatic leukodystrophy |  |  |
| AD | | Indication based Exome | *NKX2-1* (NM_001079668.2): c.727C>T; p.(Arg243Cys) | PP1, PM2, PM5, PP3, PP4 | 14:36986962 | Reported | Het | ? NA(Symptomatic father) | *NKX2-1* Related Disorders | *Screening for hypothyroidism and subsequent thyroxine therapy* |  |
| X-linked Recessive | | Indication based Exome | *DMD* (NM_004006.3): c.(7309+1_7310-1)_(8217+1_8218-1)del | PVS1, PM2, PP4, PM3 | X:31792309-31645791 | REPORTED | Hemi. | NA | Duchenne muscular dystrophy | (1) long term steroids, (2) consideration for emerging gene-based therapies (e.g. exon skipping) (3)Cardiology referral |  |
| X-linked Dominant | | Indication based Exome | *G6PD* (NM_001042351.3): c.563C>T; p.(Ser188Phe) | PS4, PS3, PP4 | X:153762634 | Reported | Het | NA | G6PD deficiency | Avoidance of foods and drugs that can lead to haemolysis. |  |
| AR | |  | *HBB* (NM_000518.5): c.20A>T; p.(Glu7Val) | PM3_VeryStrong, PS3_VeryStrong, PP4 | 11:5248232 | Reported | Cmpd Het. | NA | Sickle cell anemia / abnormal hemoglobin types |  |  |
|  |  |  | *HBB* (NM_000518.5): c.92+5G>C; p.? | PM3_VeryStrong, PS3_VeryStrong, PP4 | 11:5248155 | Reported |  |  | β-thalassemia |  |  |
| X-Linked Recessive | | Indication based Exome | *DMD* (NM_004006.3): c.(6438+1_6439-1)_(8217+1_8218-1)del | PVS1, PM2, PP4, PM3 | X:31986631-31645791 | REPORTED | Hemi. | Mother | Duchenne muscular dystrophy | (1) long term steroids, (2) consideration for emerging gene-based therapies (e.g. exon skipping) (3)Cardiology referral |  |
| AD | | Indication based Exome | *EXT1* (NM_000127.2): c.1468dupC; p.(Leu490Profs*31) | PVS1, PS4, PM2, PP4 | 8:118831983 | Reported | Het | NA | Hereditary multiple osteochondromas | On routine follow up with orthopaedics, will need resection surgery in future. |  |
| AD | | Indication based Exome | *NF1 (*NM_000267.3): c.2410-12T>G; p.? | PS4_Supporting, PM2, PP4, PS3_Moderate, PP3 | 17:29556031 | Reported | Het. | NA | Neurofibromatosis type 1 | Follow American academy of paediatrics guidelines. Health Supervision for Children with Neurofibromatosis Type 1 |  |
| AD | | Indication based Exome | *FGFR3* (NM_000142.4): c.1620C>A; p.(Asn540Lys) | PS4, PM5, PM2, PS3 | 4:1807371 | Reported | Het. | NA | Hypochondroplasia |  |  |
| AR | | Indication based Exome | *SNX10* (NM_001199835.1): c.213-2A>G; p.? | PVS1, PM2, PP4 | 7:26404665 | Novel | Hom | NA | Osteopetrosis | Treated with hematopoietic stem cells transplantation |  |
| X-linked Recessive / X-linked Dominant | | Indication based Exome | *EDA* (NM_001399.5): c.553_588del; p.(Asn185_Pro196del) | PS4, PS2, PM2, PP4 | X:69247727 | Reported | Hemi | NA | Ectodermal dysplasia |  |  |
| AR | |  | *ANO5* (NM_213599.3): c.191dup; p.(Asn64Lysfs*15) | PVS1, PM2, PP4 | 11:22242647 | Reported | Hom | NA | *ANO5* Muscle Disease |  |  |
| AR | | Indication based Exome | *IGHMBP2* (NM_002180.3): c.2540del; p.(Gln847Argfs*131) | PVS1, PM3, PP4 | 11:68704488 | Reported | Hom | NA | Spinal muscular atrophy with respiratory distress /Charcot-Marie-Tooth disease | Tracheostomy and long-term ventilation, not a candidate for current gene therapy |  |
| AR | | Indication based Exome | *RDH12* (NM_152443.3): c.506G>A; p.(Arg169Gln) | PM3_VeryStrong, PM2, PM5, PP3, PP4 | 14:68193755 | Reported | Hom | NA | Retinal dystrophy |  |  |
| AR | | Whole exome Trio | *CPLANE1* (NM_023073.3): c.c.8797-2A>G; p.? | PVS1, PM2, PP4 | 5:37122592 | Novel | Hom | Mother and Father | Joubert syndrome |  |  |
| AR | | Indication based Exome | *CHRNE (*NM_000080.4): c.966_967ins103; p.(Val323*) | PVS1, PM2, PP4 | 17:4802828 | Novel | Hom | Mother and Father | Myasthenic syndrome | Started the patient on Pyridostigmine |  |
| AR | | Indication based Exome | *SFTPB* (NM_198843.3): c.68-8A>G; p.? | PM2, PP3, PP4 | 2:85894937 | Novel | Hom | NA | Pulmonary surfactant metabolism dysfunction | Suggestive of lung transplant |  |
| AR | | Indication based Exome | *NEB (*NM_001271208.2): c.24559C>T; p.(Arg8187*) | PVS1, PM3, PM2 | 2:152352822 | Reported | Hom | NA | Nemaline myopathy |  |  |
| AR | | Indication based Exome | *MMACHC* (NM_015506.3): c.271dup; p.(Arg91Lysfs*14) | PVS1, PM3_VeryStrong | 1:45973217 | Reported | Hom | NA | Methylmalonic aciduria and homocystinuria, cblC type | Humemar et al 2017 guidelines Guidelines for diagnosis and management of the cobalamin-related remethylation disorders cblC, cblD, cblE, cblF, cblG, cblJ and MTHFR deficiency |  |
| AR | | Indication based Exome | *CDH3* (NM_001793.6): c.830del; p.(Gly277Alafs*20) | PVS1, PM3_Strong, PM2 | 16:68713839 | Reported | Hom | NA | Congenital hypotrichosis with juvenile macular dystrophy |  |  |
| AR | | Whole exome Trio | *PTRH2* (NM_016077.5): c.324G>A; p.(Trp108*) | PVS1, PM3, PP2, PM2 | 17:57775016 | Reported | Hom | Mother and Father | Infantile-onset multisystem neurologic, endocrine, and pancreatic disease | Assessment for Thyroid dysfunction, Evaluation for Diabetes |  |
| AR | | Indication based Exome | *FRRS1L (*NM_014334.4): c.246dup; p.(Phe83Leufs*22) | PVS1, PM2, PP4 | 9:111911993 | Novel | Hom | NA | Developmental and epileptic encephalopathy |  |  |
| AD | | Indication based Exome | *SPTB* (NM_001355436.2): c.1628G>A; p.(Trp543*) | PVS1, PM3, PM2 | 14:65262071 | Novel | Het | NA | Spherocytosis |  |  |
| AD | | Indication based Exome | *GABRB2* (NM_021911.2): c.902A>T; p.(Tyr301Phe) | PM2, PM5, PM1, PP3, PP4 | 5:160758065 | Reported | Het | NA | Developmental and epileptic encephalopathy |  |  |
| AD | | Indication based Exome | *GATA3* (NM_001002295.2): c.708del; p.(Ser237Alafs*29) | PVS1, PM2, PS4_Supporting | 10:8100728 | Reported | Het | NA | Hypoparathyroidism, sensorineural deafness, and renal dysplasia syndrome |  |  |
| AR/AD | | Indication based Exome | *LMNA* (NM_170707.4): c.116A>G; p.(Asn39Ser) | PS4, PM6, PM2, PM1, PM5, PP3 | 1:156084825 | Reported | Het | NA | LMNA-related disorders |  |  |
| AR | | Indication based Exome | *MPV17* (NM_002437.5): c.280G>C; p.(Gly94Arg) | PM3_Strong, PM2, PP3 | 2:27535456 | Reported | Hom | NA | Mitochondrial DNA depletion syndrome | Liver transplant |  |
| AD | | Indication based Exome | *KCNQ2* (NM_172107.4): c.1678C>T; p.(Arg560Trp) | PS4, PS2, PM2, PM5, PM1 | 20:62044888 | Reported | Het | NA | KCNQ2-Related Disorders | Opted for antiepileptics with sodium channel blocking mechanism |  |
| AR | | Indication based Exome | *POMT1* (NM_007171.3): c.793C>T; p.(Arg265*) | PVS1, PM3, PM2 | 9:134385674 | Reported | Hom | NA | Muscular dystrophy-dystroglycanopathy |  |  |
| AR | | Indication based Exome | *ABCA4* (NM_000350.2): c.3642_3644del; p.(His1215del) | PM3, PM2, PP3, PP2, PP4 | 1:94502870 | Reported | Hom | NA | Stargardt disease / Retinitis pigmentosa |  |  |
| AD | | Whole exome Trio | *NSD1* (NM_022455.5): c.4242del; p.(Glu1414Aspfs*5) | PVS1, PM2, PP4 | 5:176666804 | NOVEL | Het | De Novo | Sotos syndrome |  |  |
| AD | | Indication based Exome followed by whole exome trio re-analysis | *RNF13* (NM_007282.4) c.901G>T p.(Glu301*) | PVS1, PS2, PM2, PM5 | 3:149678646 | NOVEL | Het | De Novo | Developmental and epileptic encephalopathy |  |  |
| AR | | Whole exome Trio | *AGXT* (NM_000030.3): c.466G>A; p.Gly156Arg | PM3_VeryStrong, PM2, PS3 | 2:241810808 | Reported | Hom | Mother and Father | Hyperoxaluria | (1) novel RNA therapy (2) Combined liver and kidney transplant. |  |
| AR | | Indication based Exome | *MCIDAS* (NM_001190787.3): c.607-1G>T; p.? | PVS1, PM2, PP4 | 5:54516955 | Novel | Hom | NA | Primary ciliary dyskinesia | Pneumococcal vaccine |  |
| AD | | Whole exome Trio | *ARID1B* (NM_001374820.1): c.4359+1G>T; p.? | PVS1, PM2, PS2, PM5 | 6:157520042 | NOVEL | Het | De Novo | ARID1B-related disorder |  |  |
| AR | | Whole exome Trio | *EMC10* (NM_206538.4): c.287del; p.(Gly96Alafs*9) | PVS1, PM3_VeryStrong, PM2 | 19:50982310 | Reported | Hom | Mother and Father | Neurodevelopmental disorder with dysmorphic facies and variable seizures |  |  |
| X-linked dominant | | Indication based Exome | SMC1A (NM_006306.4): c.127G>A; p.(Asp43Asn) | PM2, PP3, PM5, PS2 | X:53442101 | Novel | Het | De Novo | SMC1A-related disorder |  |  |
| AD | | Indication based Exome | *NF1* (NM_000267.3): c.3826C>T; p.(Arg1276*) | PVS1, PS4, PM2, PP4 | 17:29562746 | Reported | Het mosiac | NA | Neurofibromatosis type 1 | Follow American academy of paediatrics guidelines. Health Supervision for Children with Neurofibromatosis Type 1 |  |
| AR | | Indication based Exome | *FAH* (NM_000137.4): c.1062+5G>A; p.? | PM3_VeryStrong, PS3 | 15:80472572 | Reported | Hom | NA | Tyrosinemia |  |  |
| AR | | Indication based Exome | *BTD* (NM_001370658.1): c.1270G>C; p.Asp424His | PM3_VeryStrong, PS3 | 3:15686693 | Reported | Hom | NA | Biotinidase Deficiency | Treated with Biotin |  |
| AD | | Indication based Exome | *TSC2* (NM_000548.5): c.1372C>T; p.(Arg458*) | PVS1, PS4_moderate, PM2 | 16:2112983 | Reported | Het Mosiac | NA | Tuberous sclerosis 2 | Referral to ophthalmology and nephrology, Underwent brain MRI and abdomen US |  |
| AD | | whole exome trio | *PTPN11* (NM_002834.5): c.853T>G; p.Phe285Val | PM2, PM5_Strong, PP3, PP4 | 12:112910844 | Reported | Het | Father | Noonan syndrome / Noonan syndrome with multiple lentigines / metachondromatosis | Growth hormone |  |
| AR | | Indication based Exome | *CFTR* (NM_000492.4): c.(?_2491)_(2619_?)del; p.? | PVS1, PM2, PP4 | 7:117234984-117235112 | Novel | Hom | NA | Cystic Fibrosis | Multidisciplinary follow up with pulmonology, Gastroenterology, general paediatrics, and dietician. |  |
| AD | | Indication based Exome | *CDKN1C* (NM_000076.2): c.703C>T; p.(Gln235*) | PVS1, PM2, PP4 | 11:2906017 | Reported | Het | NA | Beckwith-Wiedemann syndrome | (1) Regular check of Alpha feta protein (2) referral to neurodevelopment team |  |
| AR | | Indication based Exome | *BBS10* (NM_024685.4): c.804_805del; p.(Ser269Hisfs*34) | PVS1, PM2, PP4 | 12:76740959 | Reported | Hom | NA | Bardet-Biedl Syndrome |  |  |
| AR | | Indication based Exome | *FKBP10* (NM_021939.4): c.831dup; p.(Gly278Argfs*95) | PVS1, PM3_VeryStrong, PM2 | 17:39975559 | Reported | Hom | Mother and Father | Osteogenesis imperfecta type XI / Bruck syndrome |  |  |
| AD/AR | | Indication based Exome | *TMC1* (NM_138691.3): c.846dup; p.(Met283Tyrfs*20) | PVS1, PM2, PP1 | 9:75387431 | Novel | Hom | NA | Non syndromic Hearing loss |  |  |
| AD | | Indication based Exome | *STXBP1* (NM_003165.6): c.1315A>T; p.(Ile439Phe) | PM2, PP3, PS2 | 9:130438988 | Reported | Het | De Novo | STXBP1 related Encephalopathy with Epilepsy |  |  |
| AD | | Whole exome Trio | *FOXP1* (NM_032682.6): c.1652+5G>A; p.? | PM2, PP3, PS2 | 3:71021701 | Reported | Het | De Novo | FOXP1-related neurodevelopmental disorder | referral to nephrology, neurodevelopment and psychiatry |  |
| AR | | Whole exome Trio | *IFIH1* (NM_022168.4): c.2807+1G>A; p.? | PVS1, PM3_Strong, PS3 | 2:163124596 | Reported | Hom | Mother and Father | Susceptibility to respiratory viruses |  |  |
| AD | | Indication based Exome | *PACS2* (NM_001100913.3): c.625G>A; p.(Glu209Lys) | PS4, PS2, PP4, PM2, PS3 | 14:105834449 | Reported | Het | NA | Developmental and epileptic encephalopathy |  |  |
| X-linked | | Indication based Exome | *PCDH19* (NM_001184880.2): c.2566C>T; p.(Gln856*) | PVS1, PM2, PP4 | X:99657572 | NOVEL | Het | NA | PCDH19-related epilepsy | neurodevelopmental referral |  |
| AD/AR | | Indication based Exome | *SLC5A7* (NM_021815.5): c.320G>A; p.(Arg107His) | PM3_Strong, PM2, PP3 | 2:108609455 | Reported | Hom | NA | Distal hereditary motor neuronopathy / congenital myasthenic syndrome |  |  |
| X-linked Dominant | | Indication based Exome | *G6PD* (NM_001042351.3): c.563C>T; p.(Ser188Phe) | PS4, PS3, PP4 | X:153762634 | Reported | Het | NA | G6PD deficiency | Avoidance of particular medications and triggerers |  |
| AR | | Indication based Exome | *BTD* (NM_001370658.1): c.1270G>C; p.(Asp424His) | PM3_VeryStrong, PS3 | 3:15686693 | Reported | Cmpd Het. | Mother | Biotinidase deficiency | Lifelong Biotin supplementation |  |
|  |  |  | *BTD* (NM_001370658.1): c.1429C>T; p.(Pro477Ser) | PM3_VeryStrong, PM2, PP4 | 3:15686852 | Reported |  | Father |  |  |  |
| AR | | Indication based Exome | *CYP21A2* (NM_000500.9): c.293-13C>G; p.? | PM3_VeryStrong, PS3 | 6:32006858 | Reported | Hom | NA | 21-Hydroxylase-Deficient Congenital Adrenal Hyperplasia | Cortisol replacement, sick day plan |  |
| AR | | Indication based Exome | *LAMA2* (NM_000426.4): c.3829C>T; p.(Arg1277*) | PVS1, PM3, PM2 | 6:129637000 | Reported | Hom | NA | Limb-girdle muscular dystrophy | maintain adequate hydration, avoid high-intensity exercise, Regular ECHO |  |
| X-linked Dominant | | Indication based Exome | *CASK* (NM_003688.3): c.1915C>T; p.(Arg639*) | PVS1, PS2, PM2 | X:41413096 | Reported | Het | DeNovo | CASK disorders |  |  |
| AD | | Indication based Exome | *DEPDC5* (NM_001242896.3): c.1093_1103del; p.(Asp365Hisfs*16) | PVS1, PM2, PP4 | 22:32200150 | NOVEL | Het | NA | DEPDC5-Related Epilepsy |  |  |
| AD | | Whole exome Trio | *GABRB3* (NM_000814.6): c.756G>A; p.(Met252Ile) | PM2, PP3, PS2, PM1 | 15:26812807 | NOVEL | Het | DeNovo | Developmental and epileptic encephalopathy |  |  |
| AR | | Indication based Exome | *SPINK5* (NM_006846.4): c.882+1_882+3del; p.? | PVS1, PM3, PM2 | 5:147475466 | Reported | Hom | NA | Netherton syndrome |  |  |
| AR | | Indication based Exome | *ATM* (NM_000051.4): c.8977C>T; p.(Arg2993*) | PVS1, PM3_VeryStrong, PM2 | 11:108235935 | Reported | Cmp Het | NA | Ataxia-telangiectasia | Immunological screening, referral to physcial therapy, moniroting of pulmonary function, avoidance of radiation , cancer surveillance |  |
| AR | |  | *ATM* (NM_000051.4): c.8787-1G>A; p.? | PVS1, PM2, PM3 | 11:108225537 | Novel |  | NA |  |  |  |
| X-linked Dominant | | Whole exome Trio | *PDHA1* (NM_000284.4): c.1142_1145dup; p.(Trp383Serfs*6) | PVS1, PS4, PM2, PS2 | X:19377738 | Reported | Het | DeNovo | Pyruvate dehydrogenase E1-alpha deficiency | ketogenic diet |  |
| AR | | Indication based Exome | *AVIL* (NM_006576.3): c.595C>T; p.(Arg199*) | PVS1, PP4 | 12:58204298 | Novel | Hom | NA | Nephrotic syndrome |  |  |
| AD | |  | *PMS2* (NM_000535.7): c.2192_2196delTAACT; p.(Leu731Cysfs*3)  **(Secondary Finding)** | PVS1, PM3_Strong, PM2 | 7:6018306 | Reported | Het | NA | Secondary finding: Lynch Syndrome |  |  |
| AR | | Indication based Exome | CCNO (NM_021147.5): c.307C>T; p.(Gln103*) | PVS1, PM3, PM2 | 5:54529045 | Reported | Hom | NA | Primary ciliary dyskinesia | Chest clearance therapy |  |
| AR | | Indication based Exome | GJB2 (NM_004004.6): c.35del; p.(Gly12Valfs*2) | PVS1_PM3_VertStrong | 13:20763686 | Reported | Hom | NA | Deafness | cochlear implants |  |
| AR | | Indication based Exome | *HBB* (NM_000518.5): c.20A>T; p.Glu7Val | PM3_VeryStrong, PS3_VeryStrong, PP4 | 11:5248232 | Reported | Cmpd Het | NA | Sickle cell anemia / abnormal hemoglobin types Β- Thalassemia | started on Folic acid and penicillin V |  |
|  |  |  | *HBB* (NM_000518.5): c.93-22_95del; p.? | PM3_VeryStrong, PS3_VeryStrong, PP4 | 11:5248027 | Reported |  |  |  |  |  |
| AR | | Whole exome Trio | *DGAT1* (NM_012079.6): c.1095G>A; p.(Trp365*) | PVS1, PM2 | 8:145540915 | NOVEL | Hom | Mother and Father | Protein-losing enteropathy type diarrhea | Life-long TPN, sugegsting intestinal transplant |  |
| AD | | Whole Exome Proband | *GLMN* (NM_053274.3): c.1406_1409del; p.(Asp469Glyfs*6) | PVS1, PM2, PP4 | 1:92729179 | NOVEL | Het | NA | Hereditary glomuvenous malformations |  |  |
| AR | | Whole exome Trio | *NCKAP1L* (NM_005337.5): c.784+2T>G; p.? | PVS1, PM2 | 12:54905637 | NOVEL | Hom | Mother and Father | Immunodeficiency with autoinflammation |  |  |
| X-linked Recessive | | Indication based Exome | *DMD* (NM_004006.3): c.1966C>T; p.(Gln656*) | PVS1, PS2, PM2, | X:32583845 | Reported | Het | De Novo | Dystrophinopathy |  |  |
| AR | | Indication based Exome | *HBB* (NM_000518.5): c.20A>T; p.Glu7Val | PM3_VeryStrong, PS3_VeryStrong, PP4 | 11:5248232 | Reported | Cmpd Het | NA | Sickle cell anemia / abnormal hemoglobin types Β- Thalassemia | started on Folic acid and penicillin V |  |
|  |  |  | *HBB* (NM_000518.5): c.92+5G>C; p.? | PM3_VeryStrong, PS3_VeryStrong, PP4 | 11:5248027 | Reported |  |  |  |  |  |
| AR | | Indication based Exome | *PNPO* (NM_018129.4): c.363+5G>A; p.? | PM3, PP1, PM2, PP3, PS3_Supporting | 17:46022086 | Reported | Hom | NA | Pyridoxamine 5-prime-phosphate oxidase deficiency | Start Pyridoxine |  |
| AD | | Indication based Exome | *SPAST* (NM_014946.4): c.55C>G; p.(Pro19Ala) | PS2, PM2 | 2:32288955 | NOVEL | Het | De Novo | Spastic paraplegia 4 |  |  |
| AR | | Indication based Exome | *ALDH7A1* (NM_001182.5): c.1489+5G>A; p.? | PM3_VeryStrong, PS3_Moderate, PM2 | 5:125885616 | Reported | Cmpd Het | Mother | Pyridoxine-dependent Epilepsy | Start pyridoxine |  |
|  |  |  | *ALDH7A1* (NM_001182.5): c.1574A>G; p.(Asn525Ser) | PM2, PP3, PM3, PP4 | 5:125880703 | Novel |  | Father |  |  |  |
| AR | | Whole exome Trio | *HBB* (NM_000518.5): c.92+5G>C; p.? | PM3_VeryStrong, PS3_VeryStrong, PP4 | 11:5248155 | Reported | Cmpd Het | Father | Beta-thalassemia | suggesting bone marrow transplant |  |
|  |  |  | *HBB* (NM_000518.5): c.25_26del; p.(Lys9Valfs*14) | PVS1, PM3_VeryStrong, PP4 | 11:5248226 | Reported |  | Mother |  |  |  |
| AR | | Indication based Exome | *SIGMAR1* (NM_005866.4): c.270G>A; p.(Met90Ile) | PM2, PP3, PP4 | 9:34637299 | NOVEL | Hom | Mother and Father | Distal motor neuropathy |  |  |
| AR | | Indication based Exome | *UNC13D* (NM_199242.3): c.2346_2349del; p.(Arg782Serfs*12) | PVS1, PM3_VeryStrong, PP4 | 17:73830174 | Reported | Cmpd Het | Father | Hemophagocytic lymphohistiocytosis, familial, 3 | Treatment for primary HLH, suggesting gene therapy |  |
|  |  |  | *UNC13D* (NM_199242.3): c.3053C>A; p.(Ala1018Asp) | PM3, PM2, PP1, PP3, PP4 | 17:73824966 | Reported |  | Mother |  |  |  |
| AR | | Whole exome Trio | *TRAPPC12* (NM_016030.6): c.1603+5G>C; p.? | PM2, PP3, PP4 | 2:3461469 | Reported | Hom | Mother and Father | Early-onset progressive encephalopathy with brain atrophy and spasticity |  |  |
| AR/AD | | Indication based Exome | *COL4A3* (NM_000091.5): c.3752-2A>C; p.? | PVS1, PM2, PM5 | 12:52200908 | Reported | Hom | Mother and Father | Alport syndrome | suggested renal transplant |  |
| AR | | Indication based Exome | *CFTR* (NM_000492.4): c.1521_1523del; p.(Phe508del) | PVS1, PM3_VeryStrong, PS3, PP4 | 7:107342431 | Reported | Hom | NA | Cystic Fibrosis | chest clearance therapy, starting creons |  |
| AR | | Whole Exome Proband | *IQCB1* (NM_001023570.4): c.1130-1G>C; p.? | PVS1, PM3, PM2 | 16:8992432 | Reported | Hom | NA | Senior-Loken syndrome 5 |  |  |
| AR | | Whole Exome Proband | *NCF1* (NM_000265.6): c.579G>A; p.(Trp193*) | PVS1, PM3_VeryStrong, PP4 | 21:45717609 | Reported | Hom | NA | Chronic granulomatous disease | Antibiotics prophylaxis |  |
| AR | | Whole exome Trio | *TANGO2* (NM_152906.7): c.57-1G>C; p.? | PVS1, PM2 | 22:20030877 | NOVEL | Hom | Mother and Father | TANGO2-Related Metabolic Encephalopathy and Arrhythmias |  |  |
| AR | | Whole exome Trio | *METTL23* (NM_001080510.5): c.434_438del; p.(Leu145Glnfs*12) | PVS1_Strong, PM3, PM2, | 17:74729626 | Reported | Hom | Mother and Father | Intellectual disability 44 |  |  |
| AR | | Indication based Exome | *MMUT* (NM_000255.4): c.1675A>G; p.(Arg559Gly) | PM2, PM1, PP2, PP4 | 6:49412353 | Reported | Hom | NA | Methylmalonic aciduria | Emergency plan in case of metabolic decompensation, avoid nitrous oxide during surgery, caution with vaplorate or steroids |  |
| AR | | Indication based Exome | *NPHP1* NM_001128178.3:c.(?_-45)_(*443_?)del | PVS1, PM2, PP4 | Chr2: 110,504,319-111,365,996 | REPORTED | Hom | NA | Joubert Syndrome |  |  |
| AR | | Indication based Exome | *DNASE1L3* (NM_004944.4): c.290_291del; p.(Thr97Ilefs*2) | PVS1, PM3_VeryStrong, PM2 | 3:58191227 | Reported | Hom | NA | Hypocomplementemic urticarial vasculitis syndrome |  |  |
| AD | | Whole exome Trio | *IMPDH1* (NM_000883.4): c.942G>C; p.(Lys314Asn) | PM2, PS2, PM5, PP4, PP3 | 7:128038600 | NOVEL | Het | De Novo | Leber congenital amaurosis 11/ Retinitis pigmentosa 10 |  |  |
| AR | | Indication based Exome | *ALDH7A1* (NM_001182.5): c.(?_1)_(192_?)del; p.? | PVS1, PM2, PP4 | chr5:125930699-125930890 | NOVEL | Hom | NA | Pyridoxine-dependent epilepsy | Started on Pyridoxine |  |
| AD | | Indication based Exome | *KCNQ2* (NM_172107.4): c.901G>A; p.(Gly301Ser) | PS2, PM2, PS3, PM5 | 20:62070977 | Reported | Het | NA | KCNQ2-Related Disorders |  |  |
| AR/AD | | Whole exome Trio | *POLG* (NM_002693.3): c.3286C>T; p.(Arg1096Cys) | PM3_VeryStrong, PM2, PS3 | 15:89861968 | Reported | Hom | Mother and Father | POLG-related disorder Progressive external ophthalmoplegia |  |  |
| AR/AD | | Indication based Exome | *SLC3A1* (NM_000341.4): c.647C>T; p.(Thr216Met) | PM3_VeryStrong, PM2, PS3 | 2:44508562 | Reported | Hom | NA | Cystinuria |  |  |
| AR | | Indication based Exome | *CD3D* (NM_000732.6): c.202C>T; p.Arg68* | PM3_Strong, PVS1, PM2 | 11:118211162 | Reported | Hom | NA | Immunodeficiency | Bone marrow transplant |  |
| AR | | Whole exome Trio | *CPS1* (NM_001875.5): c.3608C>T; p.(Ser1203Leu) | PM2, PP3, PP4, PM5 | 2:211521298 | Reported | Hom | Mother and Father | Carbamoylphosphate synthetase I deficiency | Limit ammonia intake, ammonia scavengers |  |
| AR | | Whole exome Trio | *PJVK* (NM_001042702.5): c.667+6T>A; p.? | PM2, PP3, PP4, PP1_Moderate | 2:179323360 | NOVEL | Hom | Mother and Father | Deafness | cochlear implant |  |
| AR/AD | | Whole Exome Proband | *ACO2* (NM_001098.3): c.1534G>A; p.(Asp512Asn) | PM3, PS3, PM2, PP3 | 22:41920901 | Reported | Hom | NA | Infantile cerebellar-retinal degeneration Isolated optic atrophy |  |  |
| AD | | Whole exome Trio | *FOXG1* (NM_005249.5): c.695A>C; p.(Asn232Thr) | PM2, PM5, PM1, PP4, PS2 | 14:29237180 | NOVEL | Het | De Novo | FOXG1-related disorder |  |  |
| AR | | Indication based Exome | *SLC26A4* (NM_000441.2): c.716T>A; p.(Val239Asp) | PM3_VeryStrong, PS3, PP4 | 7:107315505 | Reported | Hom | NA | Pendred syndrome / Deafness with enlarged vestibular aqueduct | cochlear implant |  |
| AR/AD | | Indication based Exome | *MEFV* (NM_000243.3): c.2230G>T; p.Ala744Ser | PM3_VeryStrong, PP4 | 16:3293257 | Reported | Het | NA | Familial Mediterranean Fever | Colchicine |  |
| AD | | Indication based Exome | *SCN8A* (NM_014191.4): c.5638A>G; p.(Lys1880Glu) | PM2, PP3, PP4, PS2 | 12:52200908 | Reported | Het | De Novo | SCN8A-Related Epilepsy with Encephalopathy |  |  |
| AR | | Indication based Exome | *SLC26A4* (NM_000441.2): c.716T>A; p.(Val239Asp) | PM3_VeryStrong, PS3, PP4 | 7:107315505 | Reported | Hom | NA | Pendred syndrome / Deafness with enlarged vestibular aqueduct | cochlear implant |  |
| AR | | Indication based Exome | *TCN2* (NM_000355.4): c.1127dup; p.(Leu376Phefs*36) | PVS1, PM2, PP4 | 22:31018973 | NOVEL | Hom | NA | Transcobalamin II deficiency | hydroxocobalamin supplements at high doses |  |
| AR | | Indication based Exome | *SLC26A4* (NM_000441.2): c.1963A>G; p.(Ile655Val) | PM2, PP4, PM3, PP1 | 7:107342431 | Reported | Hom | NA | Pendred syndrome / Deafness with enlarged vestibular aqueduct | cochlear implant |  |
| AR | | Indication based Exome | *PDZD7* (NM_001195263.2): c.2209_2211delinsAA; p.(Gln737Asnfs*16) | PVS1, PM2, PP4 | 10:102770434 | NOVEL | Hom | NA | Deafness | cochlear implant |  |
| AR | | Indication based Exome | *SPINT2* (NM_021102.4): c.442C>T; p.(Arg148Cys) | PM3, PM2, PM5, PP3 | 19:38780809 | Reported | Hom | NA | Congenital syndromic sodium secretory diarrhea / congenital tufting enteropathy | TPN life long, suggeesting intestinal transplant |  |
| AR/AD | | Indication based Exome | *MEFV* (NM_000243.3): c.2082G>A; p.(Met694Ile) | PM3_VeryStrong, PM2, PS3, PM5 | 16:3293405 | Reported | Cmpd. Het | NA | Familial Mediterranean Fever | Colchicine |  |
| AR/AD | |  | *MEFV* (NM_000243.3): c.2177T>C; p.Val726Ala | PS4, PM3_VeryStrong, PS3 | 16:3293310 | Reported |  | NA |  |  |  |
| AD | | Indication based Exome | *USP7* (NM_003470.3): c.2596C>T; p.(Gln866*) | PVS1, PM2 | 16:8992432 | Reported | Het | NA | Hao-Fountain syndrome |  |  |
| X-linked dominant | | Indication based Exome | *CDKL5* (NM_003159.3): c.2809_2810insA; p.(Cys937*) | PVS1, PM2, PP4, PS4_Supporting | X:18668542 | Reported | Het | NA | Developmental and epileptic encephalopathy |  |  |
| AR | | Whole exome Trio | *LOXL3* (NM_032603.5): c.449delinsAA; p.(Pro150Glnfs*10) | PVS1, PM2, PP4 | 2:74777339 | NOVEL | Hom | Mother and Father | Myopia |  |  |
| AR | | Indication based Exome | *TMEM67* (NM_153704.6): c.223+1G>T; p.? | PVS1, PM2, PP4 | 8:94767366 | NOVEL | Cmpd. Het | Mother | Joubert syndrome |  |  |
|  |  |  | *TMEM67* (NM_153704.6): c.2557-3T>G; p.? | PM3, PM2, PP3, PP4 | 8:94821282 | Reported |  | Father |  |  |  |
| AD/AR | | Whole exome Trio | *AIRE* (NM_000383.4): c.1637G>C; p.(*546Serext*60) | PM2, PM4, PP3, PM5 | 21:45717609 | Reported | Hom | Mother and Father | Autoimmune polyendocrinopathy syndrome, type |  |  |
| AR | | Indication based Exome | *CARMIL2* (NM_001013838.3): c.950dup; p.(Pro318Thrfs*44) | PVS1, PM2, PP4 | 16:67681660 | NOVEL | Hom | NA | Immunodeficiency 58 | Bone marrow transplant |  |
| AR | | Indication based Exome | *SLC5A1* (NM_000343.4): c.437C>T; p.(Ser146Phe) | PM2, PP3, PP4 | 22:32464547 | NOVEL | Hom | NA | Glucose – galactose malabsorption | reduction of galactose intake |  |
| AD | | Indication based Exome | *CTRC* (NM_007272.3): c.738_761del; p.(Lys247_Arg254del) | PS4, PM2, PS3 | 1:15772183 | Reported | Het | NA | Susceptibility to chronic pancreatitis |  |  |
| AR | | Whole exome Trio | arr[GRCh37] 6q21(108,276,298_108,386,559)x0 | PVS1, PM2, PP4 | chr6:108276298-108386559 | NOVEL | Hom | Mother and Father | autosomal recessive osteopetrosis |  |  |
| AD | | Indication based Exome | *FBN1* (NM_000138.5): c.4096G>A; p.(Glu1366Lys) | PS4, PM2, PM1, PP3 | 15:48766566 | Reported | Het | NA | Marfan syndrome/ FBN1-associated disorders |  |  |
| AD | | Indication based Exome | *NF1* (NM_000267.3): c.688G>T; p.(Glu230*) | PVS1, PS4_Moderate, PP4, PM2 | 17:29508761 | Reported | Het | NA | Neurofibromatosis type 1 | Follow American academy of paediatrics guidelines. Health Supervision for Children with Neurofibromatosis Type 1 |  |
| AR | | Indication based Exome | *SCNN1A* (NM_001038.6): c.604C>T; p.(Arg202*) | PVS1, PM2, PP4 | 12:6472689 | NOVEL | Hom | NA | Pseudohypoaldosteronism type I |  |  |
| AR | | Indication based Exome | (*CFHR3*/*CFHR1* homozygous deletion) arr[GRCh37] 1q31(19674397_196801319)x1 | PVS1, PP4, PM3_Strong | 1:196743970-196801319 | REPORTED | Hom. | NA | atypical hemolytic-uremic syndrome (aHUS) | Combination of PLEX and anti-AP complement therapy, eculizumab or ravulizumab |  |
| AR | | Indication based Exome | *MVK* (NM_000431.4): c.1129G>A; p.(Val377Ile) | PM3_VeryStrong, PS3 | 12:110034320 | Reported | Hom | NA | Hyper-IgD periodic fever syndrome |  |  |
| AR | | Indication based Exome | *PAH* (NM_000277.3): c.1223G>A; p.(Arg408Gln) | PM3_VeryStrong, PM2, PM5, PS3 | 12:103234270 | Reported | Hom | NA | Phenylalanine Hydroxylase Deficiency | Dietary restriction |  |
| AD/AR | | Indication based Exome | *VWF* (NM_000552.5): c.4751A>G; p.(Tyr1584Cys) | PM3, PP1, PS3, PP3 | 12:6127833 | Reported | Hom | NA | Von Willebrand Disease | Recombinant VWF in case of bleeding |  |
| AD | | Whole exome Trio | *FBN1* (NM_000138.5): c.3350G>T; p.(Cys1117Phe) | PM2, PM5_Strong, PS2 | 15:48779622 | NOVEL | Het | NA | Marfan syndrome/ FBN1-associated disorders | Frequent ECHO |  |
| AR | | Indication based Exome | *CFTR* (NM_000492.4): c.2988+1G>A; p.? | PVS1, PM3_VeryStrong, PM2, PP4, PS3 | 7:117246808 | Reported | Hom | NA | Cystic Fibrosis | Regular airway clearance and physio |  |
| AD | | Indication based Exome | *FBN1* (NM_000138.5) c.2696G>T; p.(Gly899Val) | PM2, PS4_Supporting, PM5, PP3 | 15:48786433 | Reported | Het | NA | Marfan syndrome/ FBN1-associated disorders | Frequent ECHO |  |
| AD | | Indication based Exome | *TSC2* (NM_000548.5): c.1528C>T; p.(Gln510*) | PVS1, PS2, PM2, PP4 | 16:2114357 | Reported | Het | NA | Tuberous sclerosis 2 | Referral to ophthalmology and nephrology, Underwent brain MRI and abdomen US |  |
| AR | | Indication based Exome | *DOCK6* (NM_020812.4): c.3241-1G>A; p.? | PVS1, PM2 | 19:11332925 | NOVEL | Hom | NA | Adams-Oliver syndrome 2 |  |  |
| AD | | Indication based Exome | *LORICRIN* (NM_000427.3): c.484G>T; p.(Gly162*) | PVS1, PM2 | 1:153233909 | NOVEL | Het | NA | Loricrin keratoderma |  |  |
| AR | | Indication based Exome | *UGT1A1* (NM_000463.3): c.625C>T; p.(Arg209Trp) | PM3_Strong, PS3, PM2, PP4 | 2:234669558 | Reported | Hom | NA | Crigler-Najjar Syndrome/ Gilbert Syndrome |  |  |
| AR | |  | *UGT1A1* (NM_000463.3): c.-41_-40dupTA; p.? | PS3_VeryStrong, PM4, PP4 | 2:234668894 | NOVEL | Hom | NA | Crigler-Najjar Syndrome/ Gilbert Syndrome: UGT1A1*28 Abnormal (TA)7 |  |  |
| AR | | Whole exome Trio | *GAA* (NM_000152.3): c.2402del; p.(Glu801Glyfs*5) | PVS1, PM2, PP4 | 17:78091469 | NOVEL | Hom | Mother and Father | GAA-related glycogen storage disease 2 | Enzyme replacement therapy (ERT) |  |
| AR | | Whole Exome Duo | *HSD11B2* (NM_000196.4): c.623G>A; p.(Arg208His) | PM3, PM2, PS3, PM5 | 16:67470004 | Reported | Hom | NA | Apparent mineralocorticoid excess | Start spironolactone |  |
| AR | | Whole exome Trio | *CYP1B1* (NM_000104.4): c.1120G>A; p.(Asp374Asn) | PM2, PM3, PM5, PP3 | 2:38298377 | Reported | Hom | Mother and Father | Anterior segment dysgenesis / Primary glaucoma |  |  |
| AR | | Whole Exome Proband | *PLCE1* (NM_016341.4): c.1477C>T; p.(Arg493*) | PVS1, PM3_Strong, PM2 | 10:95892201 | Reported | Hom | NA | Nephrotic syndrome, type 3 | No role of steroids |  |
| AD | | Indication based Exome | *FLNC* (NM_001458.5): c.263del; p.(Pro88Argfs*8) | PVS1, PM2 | 7:128470952 | NOVEL | Het | NA | Cardiomyopathy Myopathy |  |  |
| AR | | Whole Exome Proband | *PKHD1* (NM_138694.4): c.107C>T; p.(Thr36Met) | PM3_VeryStrong, PM2 | 6:51947999 | Reported | Hom | NA | Polycystic kidney disease |  |  |
| X- linked Recessive | | Indication based Exome | *CD40LG* (NM_000074.3): c.430G>T; p.(Gly144*) | PVS1, PM2, PP4 | X:135741218 | NOVEL | Hemi. | NA | X-Linked Hyper IgM Syndrome | Hematopoietic stem cell transplantation |  |
| AD | | Whole exome Trio | *HIVEP2* (NM_006734.4): c.2827C>T; p.(Arg943*) | PVS1, PS2_VeryStrong, PM2, PP4 | 6:143093049 | Reported | Het | De Novo | Intellectual developmental disorder 43 |  |  |
| X-linked dominant | | Whole exome Trio | *OFD1* (NM_003611.2): c.710dup; p.(Y238Vfs*2) | PVS1, PM2, PP4, PS2 | X:13764946 | Reported | Het | De Novo | OFD1-related ciliopathy |  |  |
| AR | | Indication based Exome | GHR (NM_000163.5): c.508G>C; p.(Asp170His) | PM3, PP1, PM2, PS3, PP3, PP4 | 5:42699994 | Reported | Hom | NA | Laron syndrome | Recombinant Human IGF-I |  |
| AR | | Indication based Exome | *ABCG8* (NM_022437.3): c.965-1G>C; p.? | PVS1, PM2, PM5 | 2:44099114 | Reported | Hom | NA | Sitosterolemia | Triptorelin injection, and anastrozole |  |
| AR | | Indication based Exome | *GALT* (NM_000155.4): c.602G>A; p.(Arg201His) | PM3, PM2, PS3, PM5 | 9:34648368 | Reported | Hom | NA | Galactosemia | restrict galactose intake, Ophthalmological screening |  |
| X- linked Recessive | | Whole exome Trio | *WAS* (NM_000377.3): c.383T>C; p.(Phe128Ser) | PS4_Moderate, PM2, PP4, PM5, PP3 | X:48544145 | Reported | Hemi | Mother | WAS-related disorders | Bone marrow transplant |  |
| X- Linked Dominant | | Whole exome Trio | *WDR45* (NM_007075.4): c.619del; p.(Val207*) | PVS1, PM2, PP4, PS2 | X:48933312 | NOVEL | Het | De Novo | Beta-Propeller Protein-Associated Neurodegeneration |  |  |
|  |  |  |  |  |  |  |  |  |  |  |  |

| **Table S7. Clinically significant microarrays variants in patients with positive findings (N = 56)** | | | | | |
| --- | --- | --- | --- | --- | --- |
| **ISCN Nomenclature** | **ACMG-AMP codes** | **Size** | **CNV/ LOH** | **Syndrome/Disease** | **Management and Intervention** |
| arr[GRCh37] 4p13q11(43612839_52685687)x1 | 1A 2A | 9.07Mb | Het Del | Developmental and epileptic encephalopathy 45 | Referred to Cardiology for routine screening / Dietitian |
| arr[GRCh37] 6q24.1q24.2(141799351_145591148)x1 | 1A 2A 3A | 3.79Mb | Het Del | Mental retardation, autosomal dominant 43 |  |
| arr[GRCh37] 17p13.3(1101215_2097377)x1 | 1A 2H 3A 4A | 996Kb | Het Del | postnatal growth retardation, cognitive impairment, facial dysmorphism, Chiari type 1 malformation and white matter abnormalities. | Ophthalmology referral looking retinitis pigmentosa (associated with the deletion) |
| arr[GRCh37] Xp22.33q28(168,547_155,233,731)x1 | 1A 2A 3C | 155.03Mb | Het Del | Turner Syndrome | Endocrinology referral and U/S for kidneys |
| arr[GRCh37]15q11.2q13.1(23625784_28534245)x1 | 1A 2A 3A | 4.91Mb | Het Del | Prader Willi Syndrome |  |
| arr[GRCh37] 11q24.2q25(126678645_134938470)x1 | 1A 2H 3B 4A | 8.26Mb | Het Del | Jacobsen Syndrome | Referred to Haematology |
| arr[GRCh37] 16p13.11(15358445_16507781)x1 | 1A 2A 3A | 1.15Mb | Het Del | 16p13.11 microdeletions | Underwent a brain MRI and video EEG |
| arr[GRCh37] 16p12.2(21946522_22431357)x4, | 1A 3A 4C 5D | 485Kb | Hom Dup | Developmental delay and microcephaly, malar flattening, short palpebral fissures and abnormal nose. Clinical Features includes congenital heart disease, feeding difficulties, hypotonia, and mild motor delays. | Referral to cardiology and neurology |
| arr[GRCh37] 18p11.21(12818457_13097560)x4 | 1A 3A 4C 5D | 279Kb | Hom Dup |  |  |
| arr[GRCh37] 16q11.2q12.2(46503192_54771478)x1 | 1A 2A 3C | 8.27Mb | Het Del | 16q11.2q12.2 microdeletion syndrome Townes-Brocks syndrome | Referral to nephrology, cardiology, orthopaedics |
| arr[GRCh37] 15q11.2q13.1(23286571_28659911)x1 | 1A 2A3A | 5.37Mb | Het Del | Angelman Syndrome |  |
| arr[GRCh37] 18p11.32q23(136226_78014123)x3 | 1A 2H 3C 4A | 77.88Mb | Het Dup | Edwards Syndrome Trisomy 18 | Multidisciplinary management involving cardiology, GI, Neurology. |
| Arr[GRCh37] 22q11.21(19024657_21800797)x1 | 1A 2B 3C 4L | 2.78Mb | Het Del | DiGeorge Syndrome proximal deletion A-D | (1) evaluation for immune deficiency (2) avoid live vaccines before that (3) Endocrinology evaluation |
| arr[GRCh37] 7q11.21q36.3(62461704_159119220)x2 hmz | Not applicable | 154.64Mb | LOH/ UPD | Full chromosome 7 LoH suggestive of uniparental isodisomy (UPD) and a diagnosis of Russell-Silver Syndrome | On Somatropin and Corn-starch daily at night. |
| Arr[GRCh37] 7q11.23(72700525_74142215)x1 | 1A 2A 3B | 1.44Mb | Het Del | Williams Syndrome | (1) Cardiac evaluation (2) audiological exam (3) Thyroid function (4) Endocrinology referral |
| arr[GRCh37] 11p13(31208677_31940343)x1 | 1A 2A 3A | 732Kb | Het Del | Aniridia |  |
| arr[GRCh37] 4q34.1q35.2(174744964_190957473)x1 | 1A 2H 3C | 16.21Mb | Het Del | Craniofacial anomalies, musculoskeletal abnormalities, and intellectual disability with ocular, cardiac, genitourinary defects and pelvic/limb dysmorphism |  |
| arr[GRCh37] 15q11.2q13.3(23202070_32915593)x1 | 1A 2A 3C | 9.7Mb | Het Del | Angelman Syndrome | . |
| arr[GRCh37] 21q11.2q22.3(15006458_48097372)x3 | 1A 2H 3C 4L | 16.2Mb | Het Dup | Down Syndrome Trisomy 21 | Referred to multidisciplinary team for management and support groups |
| arr[GRCh37] 22q13.31q13.33(46435927_51175776)x1 | 1A 2A 3C | 4.74Mb | Het Del | Phelan-McDermid syndrome | Physical and occupational therapy, hearing assessment, referral to Cardiology and swallowing assessment, Orthotic assessment with splints |
| arr[GRCh37] 2q23.1(148971529_149068912)x1  (Intragenic *MBD5* deletion) | 2E: PVS1, PM2 | 97Kb | Het Del | Mental retardation, autosomal dominant 1 |  |
| arr[GRCh37] 21q11.2q22.3(15006458_48097372)x3 | 1A 2H 3C 4L | 16.2Mb | Het Dup | Down Syndrome Trisomy 21 | Referred to multidisciplinary team for management and support groups |
| arr[GRCh37] 22q11.21(18916843_21465662)x1 | 1A 2B 3C 4L | 2.54Mb | Het Del | DiGeorge Syndrome  proximal deletion A-D | (1) Evaluation for immune deficiency (2) avoid live vaccines before that (3) Endocrinology evaluation |
| arr[GRCh37] Xp22.33q28(168547_155233731)x3 | 1A 2A 3C | 155.07Mb | Het Dup | TripleX Syndrome |  |
| arr[GRCh37] 4q34.3q35.2(183182681_190957473)x1 | 1A 3C | 7.77Mb | Het Del | Two pathogenic copy number variants were identified on two separate chromosomes, which is suggestive of an unbalanced translocation | Anticipated disorders in the future, and planning screening in the future. |
| Arr[GRCh37] 18q21.2q23(52890318_78014123)x3 | 1A 2K 3C | 25.1Mb | Het Dup |  |  |
| arr[GRCh37] 2q11.1q11.2(96,732,520_98,249,638)x1 | 1A 2A 3A | 1.5Mbp | Het Del | 2q11.1q11.2 microdeletion syndrome |  |
| arr[GRCh37] 15q11.2q26.3(22752399_102429049)x2 hmz | Not applicable | 79.68Mb | LOH/ UPD | Angelman Syndrome |  |
| arr[GRCh37] 12p13.33p11.1(173787_34835837)x3-4 | 1A 2H 3C | 34.66Mb | Mosaic Hom Dup | Pallister-Killian mosaic syndrome. | . |
| arr[GRCh37] 12p13.33p11.1(173787_34835837)x3 | 1A 2H 3C | 34.66Mb | Het Dup | Trisomy 12p |  |
| arr[GRCh37] 10p15.3p14(116046_8337621)x1 | 1A 2A 3C | 8.2Mb | Het Del | 10p15.3p14 contiguous deletion syndrome (HDR Syndrome) |  |
| arr[GRCh37] Xp22.33q21.33(168547_96275443)x1-2 | 1A 2A 3C | 96.1Mb | Mosiac Het Del | Turner Syndrome? | Estrogen replacement, cardiac anomalies screening, growth hormone |
| arr[GRCh37] Xq22.1q28(98849076_155233731)x1 | 1A 2A 3C | 56.3 Mb | Het Del |  |  |
| arr[GRCh37] Xp22.33p11.21(168547_55476636)x1 | 1A 2A 3C | 55.3Mb | Het Del | Turner Syndrome? | Estrogen replacement, cardiac anomalies screening, growth hormone |
| arr[GRCh37]Xp11.21q28(55548946_155233731)x3 | 1A 2A 3C | 99.7 Mb | Het Dup |  |  |
| arr[GRCh37] 13q34(112059331_115107733)x1 | 1A 3B 4C 4L | 3.0Mb | Het Del | 13q34 terminal deletion |  |
| arr[GRCh37] 3p24.3p22.1(23810041_43429821)x3 | 1A 2H 3C | 19.62Mb | Het Dup | 19.6Mb duplication at 3p24.3p22.1 |  |
| arr[GRCh37] 7q11.23(72643632_74142190)x1 | 1A 2A 3B | 1.5Mb | Het Del | Williams-Beuren Syndrome | (1) Cardiac evaluation (2) audiological exam (3) Thyroid function (4) Endocrinology referral |
| arr[GRCh37] 18q21.2q23(48935926_78014123)x3 | 1A 2H 3C 4A | 29.1Mb | Het Dup | 29.1Mb 18q21.2q23 duplication |  |
| arr[GRCh37] Xp22.33q28(168547_155233731)x2-3 | 1A 2A 3C | 155 Mb | Het Mosiac | Mosaic chromosome X duplication/ Mosiac Turner Syndrom | Estrogen replacement, cardiac anomalies screening, growth hormone |
| arr[GRCh37] 15q11.2(22,770,422_23,688,962)x1 | 1A 2A 3A | 919kbp | Het Del | 15q11.2 microdeletion syndrome |  |
| arr[GRCh37]4q32.2q34.2(162,097,160_177,074,674)x1 | 1A 3C 4C | 14.98Mb | Het Del | 14.98Mb Partial 4q32.2q34.2 deletion | Multidiciplinary follow up with Dietician,ENT, Opthalmolog, Cardiology |
| arr[GRCh37] 22q11.21(18,648,867_21,800,471)x3 | 1A 2A 3C | 3.2Mb | Het Dup | 22q11.2 duplication syndrome |  |
| arr[GRCh37] 15q11.2(22,770,422-23,282,799)x1 | 1A 2A 3A | 512kbp | Het Del | 15q11.2 microdeletion syndrome |  |
| arr[GRCh37] 7q11.23(72,643,632_74,142,190)x1 | 1A 2A 3B | 1.5Mb | Het Del | Williams-Beuren Syndrome | (1) Cardiac evaluation (2) audiological exam (3) Thyroid function (4) Endocrinology referral |
| arr[GRCh37] 14q11.2q13.1(20,511,673_35,017,859)x1 | 1A 2A 3C | 14.5Mb | Het Del | 14.5Mb 14q11.2q13.1 interstitial deletion |  |
| arr[GRCh37] 5p15.33p15.2(113,577_14,093,389)x1 | 1A 2H 3C 4A | 14Mb | Het Del | Cri-Du-chat syndrome |  |
| arr[GRCh37] 7q11.23(72,645,014_74,142,190)x1 | 1A 2A 3B | 1.5Mb | Het Del | Williams-Beuren Syndrome | (1) Cardiac evaluation (2) audiological exam (3) Thyroid function (4) Endocrinology referral |
| arr[GRCh37] 18p11.32q23(136,227_78,014,123)x3 | 1A 2H 3C 4A | 77.9Mb | Het Dup | Edwards Syndrome Trisomy 18 | Multidisciplinary management involving cardiology, GI, Neurology. |
| Arr[GRCh37] 13q11q34(19,436,287_115,107,733)x3 | 1A 2H 3C 4A | 95.67Mb | Het Dup | Patau Syndrome Trisomy 13 | Referral to multidisciplinary management |
| arr[GRCh37] 21q11.2q22.3(15,206,017_48,097,372)x3 | 1A 2H 3C 4A | 32.9Mb | Het Dup | Down Syndrome Trisomy 21 | Referred to multidisciplinary team for management and support groups |
| arr[GRCh37] 15q11.2q13.1(22,770,422_28,981,826)x1 | 1A 2A 3B | 6.2Mb | Het Del | Prader Willi or Angelman syndrome | Dietitian / neurodevelopment follow up |
| arr[GRCh37] 6q21(108,276,298_108,386,559)x0 | 1A 3A 4A 5A | 110Kb | Hom Del | autosomal recessive osteopetrosis |  |
| arr[GRCh37] Xp22.33q28(168,567_155,233,731)x1 | 1A 2A 3C | 155Mb | Het Del | Turner Syndrome | Estrogen replacement, cardiac anomalies screening, growth hormone |
| arr[GRCh37] 7q34q36.3(139,213,938_159,119,707)x3 | 1A 2H 3C | 19.9Mb | Het Dup | 7q microduplication syndrome | muscle and nerve biopsy |
| arr[GRCh37] 17p12p11.2(15,754,174_20,552,548)x3 | 1A 2A 3C | 4.8Mb | Het Dup | Potocki-Lupski syndrome |  |
| arr[GRCh37] 13q11q34(19,436,287_115,038,009)x3 | 1A 2H 3C 4A | 95.6Mb | Het Dup | Patau Syndrome Trisomy 13 | Referral to multidisciplinary management |
| arr[GRCh37] 8p23.3p23.2(158,049_2,411,410)x1 | 1A 3A 4C 4L | 2.25Mb | Het Del | 8p inverted duplication/deletion syndrome |  |
| arr[GRCh37] 8p23.2p22(2,412,280_17,131,869)x3 | 1A 2A 3C | 14.7Mb | Het Dup |  |  |
| arr[GRCh37] 4q31.1(140,294,194_140,401,566)x1 | 1A 2D-4 3A | 107kb | Het Del | 4q31.1 microdeletion |  |
| arr[GRCh37] Xp22.33q28(535,235_155,233,731)x3 | 1A 2A 3C | 154.7Mb | Het Dup | 48,XXXY syndrome | Hormone therapy |
| arr[GRCh37] 8p23.1(8175258_11858261)x1 | 1A 2A 3A | 3.68Mb | Het Del | 8p23.1 deletion syndrome |  |

| **Table S8. Cases with extended regions of loss of heterozygosity by microarrays** | | | | | | | | | |
| --- | --- | --- | --- | --- | --- | --- | --- | --- | --- |
|  |  | Overall | | Positive CMA Diagnosis | | Inconclusive CMA Diagnosis | | Negative CMA Diagnosis | |
|  |  | Total no. | Average (LOH Interval) | Total no. | Average (LOH Interval) | Total no. | Average (LOH Interval) | Total no. | Average (LOH Interval) |
| Autosome LOH% | |  |  |  |  |  |  |  |  |
|  | 1-4% | 32 | 2.21% | 6 | 2.29% | 8 | 2.08% | 18 | 2.25% |
|  |  |  | (1.00% - 3.78%) |  | (1.08% - 3.30%) |  | (1.00% -3.78%) |  | (1.00% -3.63%) |
|  | 4-10% | 54 | 6.95% | 7 | 7.27% | 15 | 6.68% | 32 | 7.01% |
|  |  |  | (4.00% - 9.98%) |  | (4.04% - 9.95%) |  | (4.00% - 9.51%) |  | (4.00% - 9.98%) |
|  | >10% | 34 | 12.83% | 2 | 10.82% | 10 | 12.24% | 22 | 13.29% |
|  |  |  | (10.12% - 18.60%) |  | (10.23% - 11.40%) |  | (10.23% - 15.43%) |  | (10.12% - 18.6%) |
| Overall | | 120 | 7.36% | 15 | 5.75% | 33 | 7.25% | 72 | 7.74% |
|  |  |  | (1.00% - 18.60%) |  | (1.08% - 11.40%) |  | (1.00% - 15.43%) |  | (1.00% - 18.6%) |

**Table S9. Candidate genes**

| **Patient organ system involved/Testing performed** | **Candidate gene** | **Variant (Allele Frequency)** | **Gene Information** |
| --- | --- | --- | --- |
| Nervous system/Chromosome microarray analysis (negative) and trio whole exome sequencing (inconclusive) | *SOAT2* | NM_003578.3:c.1017_1020delTATC; NP_003569.1:p.Ile340Cysfs*81  gnomAD allele frequency: 0.004% | *SOAT2* gene encodes sterol O-acyltransferase 2. This gene is involved in cholesterol metabolism pathway.  Animal models suggest a role in liver/biliary system. Gene is ubiquitously expressed across human tissues (The Genotype-Tissue Expression, GTEx, Project). |
| Multisystem/ Trio whole exome sequencing (inconclusive) | *AOX1* | (NM_001159.3:c.1303G>T; NP_001150.3:p.Glu435*)  gnomAD allele frequency: 0.003% | *AOX1* gene encodes aldehyde oxidase 1 and is involved in Purine and NAD metabolism pathway. This gene is intolerant to heterozygous loss of function variants in gnomAD. It is ubiquitously expressed across human tissues (The Genotype-Tissue Expression, GTEx, Project). |
| Renal system/Chromosome microarray analysis (negative) and trio whole exome sequencing (inconclusive) | *MYBPC2* | NM_004533.3:c.1522_1523dupTA; NP_004524.3:p.Phe510Argfs*54  gnomAD allele frequency: 0.002% | *MYBPC2* gene encodes myosin binding protein C, fast type. This gene is involved in Cardiac conduction and Striated muscle contraction pathway. This gene is intolerant to heterozygous loss of function variants in gnomAD. It is ubiquitously expressed across human tissues (The Genotype-Tissue Expression, GTEx, Project). |
| Nervous system/Chromosome microarray analysis (negative) and trio whole exome sequencing (inconclusive) | *CYP4X1* | NM_178033.1:c.938delT; NP_828847.1:p.Phe313Serfs*22  Not reported in gnomAD | *CYP4X1* gene encodes cytochrome P450, family 4, subfamily X, polypeptide 1. This gene is involved in Oxidation by cytochrome P450 pathway. It is ubiquitously expressed across human tissues (The Genotype-Tissue Expression, GTEx, Project). |
| Nervous and endocrine systems/Trio whole exome sequencing (inconclusive) | *DTHD1* | NM_001170700.3:c.1218+1G>T; p.?  gnomAD allele frequency: 0.03% | *DTHD1* gene encodes death domain containing 1. This gene is mainly involved in apoptosis pathway. This gene is intolerant to heterozygous loss of function variants in gnomAD. It is strictly expressed in lung, visceral tissues, thyroid, spleen, stomach, whole blood among others. In brain, it is restricted to amygdala, basal ganglia, hippocampus, and the hypothalamus. It is absent from vascular system, heart, kidney, liver, pancreas and skeletal muscles. |
| Multisystem/Chromosome microarray analysis (negative) and trio whole exome sequencing (inconclusive). ***Note this case is now considered positive in Table S6 based on ongoing unpublished functional work proving gene-disease association*** | *FBXO22* | NM_147188.3:c.159_162delGGAG; NP_671717.1:p.Arg53Serfs*13  Not reported in gnomAD | *FBXO22* gene encodes F-box protein 22 which is a component of the ubiquitin-mediated proteasomal degradation pathway. This gene is intolerant to heterozygous loss of function variants in gnomAD and is ubiquitously expressed across human tissues (The Genotype-Tissue Expression, GTEx, Project). |
| Nervous system/Trio Whole exome analysis (inconclusive) | *MAN2B2* | NM_015274.3:c.138+1G>A; p.?  -  gnomAD allele frequency: 0.003% | *MAN2B2* gene encodes mannosidase, alpha class 2B, member 2. This gene is involved in Lysosomal oligosaccharide catabolism pathway. It is ubiquitously expressed across human tissues (The Genotype-Tissue Expression, GTEx, Project). |
| Neuromuscular system/Exome-based custom neuromuscular panel (inconclusive) | *SATL1* | NM_001367857.2:c.1445G>A  NP_001354786.1:p.Trp482Ter  Not reported in gnomAD | *SATL1* gene encodes spermidine/spermine N1-acetyl transferase-like 1. Diseases associated with this gene include Non-Syndromic X-Linked Intellectual Disability 97 and Exotropia. This gene is intolerant to heterozygous loss of function variants in gnomAD and is ubiquitously expressed across human tissues (The Genotype-Tissue Expression, GTEx, Project). |

|  | | | | | | | | |
| --- | --- | --- | --- | --- | --- | --- | --- | --- |
| **Table S10. Sequencing Variants of Uncertain Clinical Significance (VUS)** | | | | | | | | |
| Variants (cDNA; protein) | ACMG-AMP CODES | Genomic Coordinates | Novel Or reported | Mode of Inheritance | Zygosity | Classification | Parent of Origin | Clinical indication for testing |
| *L2HGDH* (NM_024884.2):c.713A>G; p.(Tyr238Cys) | PM2, PP3 | 14:50745263 | Reported | AR | Het | VUS | ? | hearing loss |
| *L2HGDH* (NM_024884.2):c.41G>A; p.(Arg14Gln) | PM2, BP4 | 14:50778828 | Novel | AR | Het | VUS | ? | hearing loss |
| *UPB1* (NM_016327.2):c.5C>G; p.(Ala2Gly) | PM2, BP4 | 22:24891376 | Novel | AR | Hom | VUS | ? | hearing loss |
| *LARS2* (NM_015340.3):c.2620G>A; p.(Glu874Lys) | PM2, BP4 | 3:45588930 | Reported | AR | Het | VUS | ? | hearing loss |
| *SLITRK6* (NM_032229.2):c.2413T>C; p.(Tyr805His) | PM2, PP3 | 13:86368231 | Reported | AR | Het | VUS | ? | hearing loss |
| *S1PR2* (NM_004230.3):c.985C>T; p.(Arg329Cys) | PM2, PP3 | 19:10334597 | Novel | AR | Het | VUS | ? | hearing loss |
| *ALDH18A1* (NM_002860.3):c.1237G>C; p.(Glu413Gln) | PM2, BP4 | 10:97385128 | Novel | AD/AR | Het | VUS | ? | Muscle spasms |
| *PYGM* (NM_005609.3):c.2203C>T; p.(Arg735Cys) | BP4 | 11:64514805 | Novel | AR | Het | VUS | ? | Muscle spasms |
| *SERPINA6* (NM_001756.3):c.1165G>A; p.(Asp389Asn) | PM3, PS3, BS1 | 14:94770808 | Reported | AD/AR | Het | VUS | ? | Muscle spasms |
| *TRAPPC11* (NM_021942.5):c.2234C>T; p.(Thr745Ile) | PM2, PP3 | 4:184614297 | Novel | AR | Het | VUS | ? | Muscle spasms |
| *MEFV* (NM_000243.2):c.460T>C; p.(Ser154Pro) | PM2, BP4 | 16:3304608 | Reported | AD/AR | Het | VUS | Mother | Juvenile Idiopathic Arthritis |
| *ESPN* (NM_031475.2):c.1025C>T; p.(Ser342Phe) | PM2_Supporting, PP3 | 1:6504575 | Novel | AR | Het | VUS | ? | Hearing loss |
| *WHRN* (NM_015404.3):c.919A>G; p.(Thr307Ala) | PM2, PP3 | 9:117228591 | Novel | AR | Het | VUS | ? | Hearing loss |
| *PEX6* (NM_000287.3):c.1802G>A; p.(Arg601Gln) | PM3, PS3, BS1 | 6:42935188 | Reported | AR | Het | VUS | Mother | Global developmental delay |
| *CTNNA3* (NM_013266.3):c.2375T>C; p.(Leu792Pro) | PM2, PP3 | 10:67726395 | Novel | AD | Het | VUS | ? | Prolonged QT |
| *COL11A2* (NM_080680.2):c.2536C>T; p.(Arg846Trp) | PP3 | 6:33141697 | Reported | AD/AR | Het | VUS | ? | Hearing loss |
| *LMNA* (NM_170707.3):c.112C>T; p.(Leu38Phe) | PM2, PP3, PP4 | 1:156084821 | Reported | AD/AR | Het | VUS | ? | Muscle weakness |
| *LDB3* (NM_007078.3):c.47G>A; p.(Arg16His) | BP4 | 10:88428495 | Reported | AD | Het | VUS | ? | Muscle weakness |
| *PYGM* (NM_005609.3):c.2203C>T; p.(Arg735Cys) | BP4 | 11:64514805 | Novel | AR | Het | VUS | ? | Muscle weakness |
| *SETD2* (NM_014159.6):c.7346T>C; p.(Met2449Thr) | PM2, BP4 | 3:47079160 | Novel | AD | Het | VUS | ? | Developmental delay |
| *INPPL1* (NM_001567.3):c.2326+9C>T; p.? | PM2 | 11:71945447 | Reported | AR | Hom | VUS | ? | Spondyloepiphyseal dysplasia |
| *TNXB1* (NM_019105.6):c.7459C>T; p.(Arg2487Cys) | PM2, PP3 | 6:32029207 | Reported | AD/AR | Het | VUS | ? | Spondyloepiphyseal dysplasia |
| *PRICKLE1* (NM_153026.2):c.391T>G; p.(Leu131Val) | BP4 | 12:42862625 | Reported | AR | Hom | VUS | Father & Mother | Seizures |
| *PGK1* (NM_000291.3):c.1043T>G; p.(Phe348Cys) | PM2, PP3 | X:77380477 | Novel | X-linked Recessive | Hemi | VUS | Mother | Seizures |
| *SLC12A3* (NM_000339.2):c.322C>T; p.(Arg108Trp) | BP4, PM3, BP2 | 16:56901021 | Reported | AR | Het | VUS | Father | Acute kidney injury, Developmental Delay |
| *C3* (NM_000064.3):c.1402G>A; p.(Gly468Arg) | PM2, PP3 | 19:6711075 | Reported | AD/AR | Het | VUS | Mother | Acute kidney injury, Developmental Delay |
| *MYH9* (NM_002473.5):c.1306G>A; p.(Ala436Thr) | PM2, PP3 | 22:36712636 | Reported | AD | Het | VUS | Father | Acute kidney injury, Developmental Delay |
| *EP300* (NM_001429.3):c.4453G>T; p.(Asp1485Tyr) | PM2, PM6 | 22:41568503 | Novel | AD | Het | VUS | De novo | Acute kidney injury, Developmental Delay |
| *SYNE1* (NM_033071.3):c.17390G>A; p.(Gly5797Glu) | PM2, PP3 | 6:152621855 | Novel | AD/AR | Het | VUS | De novo | Skeletal dysplasia |
| *EFL1* (NM_024580.5):c.2902G>T; p.(Ala968Ser) | PM2, PP3 | 15:82443893 | Novel | AR | Het | VUS | De novo | Skeletal dysplasia |
| *TMPRSS6* (NM_153609.3):c.1869-21_1869-2del; p.? | PM2 | 22:37465398 | Novel | AR | Hom | VUS | ? | Anemia |
| *PRDM5* (NM_018699.3):c.670A>T; p.(Lys224*) | PVS1_Moderate, PM2 | 4:121738060 | Reported | AR | Het | VUS | ? | Global developmental delay |
| *EPB41L1* (NM_001258330.1):c.84+1G>A; p.? | PVS1_Moderate, PM2 | 20:34713450 | Novel | AD | Het | VUS | ? | Global developmental delay |
| *GBE1* (NM_000158.3):c.986A>G; p.(Tyr329Cys) | PM3, PS3, BS1, BS2, PP3 | 3:81691938 | Reported | AR | Het | VUS | ? | Leukodystrophy |
| *NPC1* (NM_000271.4):c.2747A>G; p.(Asn916Ser) | BP4 | 18:21119823 | Reported | AR | Het | VUS | ? | Leukodystrophy |
| *SPART* (NM_015087.5):c.466C>T; p.(Pro156Ser) | PM2, BP4 | 13:36909502 | Reported | AR | Het | VUS | ? | Leukodystrophy |
| *TBCK* (NM_001163435.2):c.1804G>A; p.(Ala602Thr) | PB4 | 4:107133963 | Reported | AR | Het | VUS | Mother | Epilepsy |
| *IKBKE* (NM_014002.3):c.541-5G>A; p.? | PM2, PP3 | 1:206650016 | Reported | AR | Het. | VUS | ? | Platelet glycoprotein IV deficiency |
| *DMGDH* (NM_013391.3):c.972G>A; p.(Trp324*) | PVS1, BS1 | 5:78340149 | Reported | AR | Het. | VUS | Mother | Global developmental delay |
| *DMGDH* (NM_013391.3):c.696del; p.(Glu233Lysfs*7) | PVS1_Supporting, PM2 | 5:78347159 | Novel | AR | Het. | VUS | Father | Global developmental delay |
| *PTPN23* (NM_015466.3):c.4844G>A; p.(Arg1615Gln) | PM2, BP4 | 3:47454608 | Novel | AR | Het. | VUS | Mother | Global developmental delay |
| *SYNJ1* (NM_003895.3):c.202G>A; p.(Glu68Lys) | PM2 | 21:34099122 | Novel | AR | Het. | VUS | Father | Global developmental delay |
| *TRAP1* (NM_016292.2):c.1204C>T; p.(Arg402Trp) | PM2, PP3 | 16:3721754 | Novel | AR | Het | VUS | Father | Juvenile idiopathic arthritis |
| *FBXO7* (NM_012179.3):c.674C>T; p.(Pro225Leu) | PM2, PP3 | 22:32881083 | Novel | AR | Hom | VUS | Mother & Father | Motor delay, Muscle spasticity |
| *CHRNA4* (NM_000744.6):c.639_640delinsCA; p.(Ala214Thr) | PM2, PP3 | 20:61982123 | Novel | AD | Het | VUS | Mother | Autism spectrum, Epilepsy |
| *PHKA1* (NM_002637.3):c.1282C>T; p.(Arg428Cys) | PM2, PP3 | X:71870282 | Novel | X-linked Recessive | Hemizygous | VUS | Mother | Right spastic hemiparesis |
| *LPIN2* (NM_014646.2):c.839G>A; p.(Arg280Gln) | PM2, BP4 | 18:2938019 | Novel | AR | Het | VUS | ? | Autoinflammatory syndrome |
| *NLRP1* (NM_033004.3):c.3248C>T; p.(Thr1083Met) | BP4 | 17:5436190 | Novel | AD/AR | Het | VUS | ? | Autoinflammatory syndrome |
| *PSMB8* (NM_148919.3):c.544G>A; p.(Gly182Arg) | PM2 | 6:32809506 | Reported | AR | Het | VUS | ? | Autoinflammatory syndrome |
| *PKHD1* (NM_138694.3):c.7912-5T>G; p.? | BP4 | 6:51712773 | Reported | AR | Het | VUS | Father | Hydrocephaly |
| *PKHD1* (NM_138694.3):c.5134G>A; p.(Gly1712Arg) | PM3, PP3, BS1 | 6:51889474 | Reported | AR | Het | VUS | Mother | Hydrocephaly |
| *LRP6* (NM_002336.2):c.4377dup; p.(Tyr1460Leufs*2) | PVS1_Moderate, PM2 | 12:12278307 | Novel | AD | Het | VUS | Mother | Hydrocephaly |
| *MACF1* (NM_012090.5):c.1832A>T; p.(Asp611Val) | PM2, PP3 | 1:39757613 | Novel | AD | Hom | VUS | Mother & Father | Microencephaly |
| *PARS2* (NM_152268.4):c.706A>G; p.(Lys236Glu) | PM2, BP4 | 1:55224129 | Novel | AR | Hom | VUS | Mother & Father | Microencephaly |
| *HSPG2* (NM_005529.6):c.12664A>C; p.(Ser4222Arg) | PM2, PP3 | 1:22151198 | Novel | AR | Het | VUS | Father | Bilateral congenital dislocation of hip, |
| *TFAP2A* (NM_003220.2):c.575C>T; p.(Ala192Val) | PM2, BP4 | 6:10404930 | Novel | AD | Het | VUS | De novo | Global developmental delay |
| *CD247* (NM_198053.2):c.58+8C>T; p.? | PM3, PM2, BP4 | 1:167487637 | Reported | AR | Het | VUS | Mother | Immune deficiency disorder |
| *DOCK2* (NM_004946.2):c.2704-3C>T; p.? | PM2, BP4 | 5:169267758 | Reported | AR | Het | VUS | Father | Immune deficiency disorder |
| *TOM1* (NM_005488.2):c.53-8C>A; p.? | PM2, PP3 | 1:167487637 | Reported | AD | Het | VUS | Father | Immune deficiency disorder |
| *CAV1* (NM_001753.5):c.407G>A; p.(Ser136Asn) | PM2, PP3 | 7:116199211 | Novel | AD | Het | VUS | ? | Pulmonary Hypertension |
| *MYO15A* (NM_016239.3):c.3620T>C; p.(Ile1207Thr) | PM2, PP3 | 17:18027807 | Novel | AR | Het | VUS | Father | Diabetes, Hearing loss |
| *MYO15A* (NM_016239.3):c.3622C>T; p.(Arg1208Cys) | PP3 | 17:18027809 | Reported | AR | Het | VUS | Mother | Diabetes, Hearing loss |
| *LOXHD1* (NM_144612.6):c.6340G>A; p.(Val2114Met) | PM2 | 18:44057731 | Novel | AR | Het | VUS | Father | Diabetes, Hearing loss |
| *OTOG* (NM_001277269.1):c.7090C>Tp.(Arg2364Trp) | PP3 | 11:17653755 | Reported | AR | Het | VUS | Father | Diabetes, Hearing loss |
| *GIPC3* (NM_133261.2):c.680G>A; p.(Gly227Glu) | PM2, BP4 | 19:3589528 | Novel | AR | Het | VUS | ? | hearing loss |
| *LARS2* (NM_015340.3):c.2285C>A; p.(Ala762Asp) | PM2, BP4 | 3:45561781 | Novel | AR | Het | VUS | ? | hearing loss |
| *TRIOBP* (NM_001039141.2):c.5422A>G; p.(Lys1808Glu) | PM2, BP4 | 22:38150926 | Novel | AR | Het | VUS | ? | hearing loss |
| *CLDN14* (NM_144492.2):c.523C>T; p.(Leu175Phe) | PM2 | 21:37833471 | Novel | AR | Het | VUS | Father | Sensory hearing loss, Global developmental delay |
| *CLRN1* (NM_174878.2):c.356T>C; p.(Phe119Ser) | PM2, PP3 | 3:150659446 | Novel | AR | Het | VUS | Father | Sensory hearing loss, Global developmental delay |
| *PHKA2* (NM_000292.2):c.473C>T; p.(Thr158Ile) | PM2, PP3 | X:18966926 | Novel | X-linked Recessive | Hemi | VUS | Mother | Thrombocytopenia, Systemic inflammatory response syndrome |
| *ZNF292* (NM_015021.2):c.6758_6760del; p.(Asp2253del) | PM2, PP3 | 6:87970104 | Novel | AD | Het | VUS | Mother | Social communication disorder |
| *PIK3CD* (NM_005026.3):c.1339+4G>A; p.? | PM2, BP4 | 1:9780079 | Reported | AD/AR | Het | VUS | ? | Acute leukemia, Abdominal pain |
| *PIK3CD* (NM_005026.3):c.1955+5C>T; p.? | PM2, BP4 | 1:9781650 | Reported | AD/AR | Het | VUS | ? | Acute leukemia, Abdominal pain |
| *CTNS* (NM_004937.2):c.1062C>A; p.(Phe354Leu) | PM2, BP4 | 17:3563621 | Novel | AR | Hom | VUS | Mother & Father | Alternating exotropia |
| *PCARE* (NM_001029883.3):c.2938C>T; p.(Pro980Ser) | PM2, BP4 | 2:29294190 | Novel | AR | Hom | VUS | Mother & Father | Alternating exotropia |
| *ATP13A2* (NM_022089.3):c.1649C>A; p.(Pro550His) | PM2, BP4 | 1:17320224 | Novel | AR | Het | VUS | Mother | Ankle contracture |
| *IRF7* (NM_001572.3):c.709G>T; p.(Gly237Trp | PM2, BP4 | 11:614008 | Novel | AR | Het | VUS | ? | Neutropenia |
| *CHRNA1* (NM_000079.4):c.670C>T; p.(His224Tyr) | PM2, PP3 | 2:175618339 | Novel | AD/AR | Hom | VUS | Mother & Father | Global developmental delay, FTT (failure to thrive) |
| *MRE11* (NM_005591.4):c.835G>C; p.(Ala279Pro) | PM2, PP3 | 11:94204750 | Reported | AR | Hom | VUS | Mother & Father | Global developmental delay, FTT (failure to thrive) |
| *SPG11* (NM_025137.4):c.1906C>G; p.(Leu636Val) | PM2, PP3 | 15:44921028 | Novel | AR | Het | VUS | Father | Global developmental delay, FTT (failure to thrive) |
| *POLD1* (NM_002691.3):c.1873G>A; p.(Gly625Arg) | PM2, PP3 | 19:50912139 | Reported | AD | Het | VUS | ? | Epidermoid cyst |
| *CC2D2A* (NM_001080522.2):c.3601G>A; p.(Gly1201Arg) | PM2, PP3 | 4:15575779 | Novel | AR | Het | VUS | ? | Joubert syndrome, Oculomotor apraxia |
| *CEP164* (NM_014956.4):c.717G>C; p.(Arg239Ser) | PM2 | 11:117234174 | Novel | AR | Het | VUS | ? | Joubert syndrome, Oculomotor apraxia |
| *DEPDC5* (NM_001242896.1):c.3515C>A; p.(Thr1172Asn) | PM2 | 22:32269270 | Reported | AD | Het | VUS | Father | Epilepsy |
| *AIRE* (NM_000383.3):c.927C>G; p.(Ile309Met) | PM2, BP4 | 21:45711025 | Reported | AD/AR | Het | VUS | ? | Inflammatory bowel disease |
| *DKC1* (NM_001363.4):c.1432_1434del; p.(Lys478del) | PM2, PP3 | X:154004555 | Novel | X-linked Recessive | Hemi | VUS | ? | Inflammatory bowel disease |
| *CACNA1C* (NM_000719.6):c.2854-11C>G; p.? | PM2, PP3 | 12:2711009 | Novel | AD | Het | VUS | ? | ATAXIA / ARRHYTHMIA PANEL |
| *TNFRSF13B* (NM_012452.3):c.283C>G; p.(Gln95Glu) | PM2, BP4 | 17:16852214 | Reported | AD/AR | Het | VUS | ? | Inflammatory bowel disease |
| *NFKB2* (NM_001322934.1):c.2042C>T; p.(Pro681Leu) | PM2, PP3 | 10:104160777 | Novel | AD | Het | VUS | ? | Severe rectal inflammation |
| *TERT* (NM_198253.3):c.604G>A; p.(Ala202Thr) | BP4 | 5:1294397 | Reported | AD/AR | Het | VUS | ? | Severe rectal inflammation |
| *MYLK* (NM_053025.4):c.3112A>Gp.(Met1038Val) | PM2, BP4 | 3:123419203 | Reported | AD/AR | Het | VUS | ? | Hypermobility Spectrum Disorder |
| *HNF1A* (NM_000545.8):c.716C>T; p.(Ala239Val) | PS3_Moderate, PP3 | 12:121431969 | Reported | AD | Het | VUS | ? | Type 1 Diabetes Mellitus |
| *CAPN3* (NM_000070.3):c.2107C>T; p.(Leu703Phe) | PM1, PP3, PM2 | 15:42702185 | Reported | AD/AR | Het | VUS | Mother | Weakness of trunk musculature |
| *LAMA2* (NM_000426.4):c.2512G>A; p.(Gly838Arg) | PM2, PP3 | 6:129601267 | Reported | AR | Het | VUS | ? | Weakness of trunk musculature |
| *TRIO* (NM_007118.4):c.157+3G>A; p.? | PM2, BP4 | 5:14143994 | Novel | AD | Het | VUS | Father | Autistic disorder |
| *CSF3R* (NM_000760.4):c.1931G>A; p.(Gly644Glu) | PM2, BP4 | 1:36933186 | Novel | AR | Het | VUS | ? | Neutropenia |
| *RHOBTB2* (NM_001160036.2):c.574C>T; p.(Pro192Ser) | PM2, PP3 | 8:2286426 | Reported | AD | Het | VUS | ? | Optic atrophy, Encephalopathy |
| *AARS1* (NM_001605.3):c.877A>T; p.(Met293Leu) | PM2, PP3 | 16:70303606 | Novel | AD | Het | VUS | ? | Neuropathy |
| *DNAJB2* (NM_001039550.2):c.154G>A; p.(Ala52Thr) | PM2, PP3 | 2:220145388 | Novel | AR | Hom | VUS | ? | Neuropathy |
| *COL4A3* (NM_000091.5):c.1418G>A; p.(Gly473Asp) | PM2, PP3 | 2:228131718 | Novel | AD | Het | VUS | ? | ESRD on peritoneal dialysis |
| *RBP3* (NM_002900.3):c.1733C>A; p.(Thr578Lys) | PP3 | 10:48389145 | Reported | AR | Hom | VUS | ? | ESRD on peritoneal dialysis |
| *RLBP1* (NM_000326.5):c.167A>T; p.(Glu56Val) | PM2, PP3 | 15:89760530 | Reported | AD/AR | Het | VUS | ? | ESRD on peritoneal dialysis |
| *MYH7B* (NM_020884.5):c.324+1G>T; p.? | PVS1_Moderate, PM2 | 20:33567300 | Reported | Unknown | Het | VUS | ? | ESRD on peritoneal dialysis |
| *COL6A2* (NM_001849.4):c.1111G>A; p.(Gly371Ser) | PM2, BP4 | 21:47537845 | Reported | AD/AR | Het | VUS | ? | Toe-walking, Muscle hypertrophy |
| *DYSF* (NM_003494.4):c.4939C>T; p.(Leu1647Phe) | PM2, BP4 | 2:71738977 | Novel | AR | Het | VUS | ? | Toe-walking, Muscle hypertrophy |
| *ANKRD11* (NM_013275.6):c.5357A>G; p.(Asn1786Ser) | PM2, BP4 | 16:8934759 | Novel | AD | Het | VUS | ? | Cornelia de Lange syndrome |
| *HECW2* (NM_020760.4):c.206C>T; p.(Thr69Met) | PM2, PP3 | 2:197297942 | Novel | AD | Het | VUS | ? | Developmental delay |
| *NRXN1* (NM_001135659.3):c.4357G>A; p.(Gly1453Ser) | PM2, PP3 | 2:50149369 | Reported | AR | Het | VUS | ? | Developmental delay |
| *LARGE1* (NM_004737.7):c.26G>A; p.(Arg9Gln) | PM2, PP3 | 22:34157438 | Reported | AR | Het | VUS | Father | Hypotonia |
| *LARGE1* (NM_004737.7):c.500C>G; p.(Pro167Arg) | PM2, PP3 | 22:34000536 | Novel | AR | Het | VUS | Mother | Hypotonia |
| *RYR1* (NM_000540.3):c.13213G>A; p.(Gly4405Ser) | PM2, BP4 | 19:39056187 | Novel | AD/AR | Het | VUS | Father | Hypotonia |
| *PRRT2* (NM_145239.3):c.745T>C; p.(Ser249Pro) | PM2 | 16:29825120 | Reported | AD | Het | VUS | ? | Chorea, Development delay |
| *SETX* (NM_015046.7):c.6248G>T; p.(Arg2083Ile) | PM2 | 9:135163699 | Reported | AD/AR | Het | VUS | ? | Chorea, Development delay |
| *SYNE1* (NM_033071.4):c.13648A>G; p.(Thr4550Ala) | PM2, BP4 | 6:152652172 | Novel | AD/AR | Het | VUS | ? | Muscle hypotonia |
| *SMARCA1* (NM_001282874.2):c.916C>T; p.(Arg306*) | PVS1_Modertate, PM2 | X:128641968 | Novel | X-linked Recessive | Hemi | VUS | Mother | Macrocephaly |
| *COL1A2* (NM_000089.4):c.896A>C; p.(Asn299Thr) | PM2, PP3 | 7:94038880 | Novel | AD | Het | ? | ? | Osteogenesis imperfecta |
| *SIK1* (NM_173354.5):c.1294C>T; p.(Arg432Trp) | PM2 | 21:44839069 | Reported | AD | Het | VUS | ? | Genetic Epilepsy with Febrile Seizures Plus |
| *CYBB* (NM_000397.4):c.1390C>A; p.(Gln464Lys) | PM2, BP4 | X:37665715 | Novel | X-linked Recessive | Hemi | VUS | ? | Crohn's disease of ileum |
| *NOD2* (NM_022162.3):c.1117C>T; p.(Arg373Cys) | BP4 | 16:50745020 | Reported | AD/Multifactorial | Het | VUS | ? | Crohn's disease of ileum |
| *ALPI* (NM_001631.5):c.895C>T; p.(Arg299*) | PVS1_Moderate | 11:62159724 | Novel | AR | Het | VUS | ? | Crohn's disease of ileum |
| *MAPK1* (NM_002745.5):c.17_22dup; p.(Ala6_Ala7dup) | PM2 | 22:22221723 | Novel | AD | Het | VUS | ? | Noonan syndrome |
| *A2ML1* (NM_144670.6):c.3701del; p.(Gly1234Alafs*9) | PVS1_Moderate, PM2 | 12:9016588 | Novel | AD/Digenic | Het | VUS | ? | Noonan syndrome |
| *LRP4* (NM_002334.4):c.5504dup; p.(Leu1836Profs*24) | PVS1_Moderate, PM2 | 11:46920581 | Novel | AD/AR | Het | VUS | ? | Suspected infectious disease |
| *SCN10A* (NM_006514.4):c.4210T>G; p.(Phe1404Val) | PM2, PP3 | 3:38751040 | Novel | AD/AR | Het | VUS | ? | Suspected infectious disease |
| *GABRB2* (NM_021911.3):c.1334G>T; p.(Gly445Val) | PM2, PP3 | 5:160721293 | Novel | AD | Het | VUS | Father | Cortical dysplasia |
| *KMT2C* (NM_170606.3):c.3466G>A; p.(Val1156Ile) | PM2, PP3 | 7:151919119 | Novel | AD | Het | VUS | De novo | Cortical dysplasia |
| *BIRC6* (NM_016252.3):c.7591T>G; p.(Tyr2531Asp) | PM2, PP3 | 2:32707545 | Novel | AD | Het | VUS | De novo | Epilepsy |
| *CDKN1C* (NM_000076.2):c.875C>T; p.(Pro292Leu) | PM2, PP3 | 11:2905277 | Novel | AD | Het | VUS | ? | Metaphyseal chondrodysplasia |
| *UFSP2* (NM_018359.5):c.1376A>C; p.(Asn459Thr) | PM2, PP3 | 4:186321580 | Novel | AD | Hom | VUS | Mother & Father | Metaphyseal chondrodysplasia |
| *G6PC1* (NM_000151.4):c.643T>C; p.(Phe215Leu) | PM2, BP4 | 17:41063012 | Novel | AR | Het | VUS | ? | Ketotic hypoglycemia |
| *GHSR* (NM_198407.2):c.422G>T; p.(Arg141Leu) | PM2, PP3 | 3:172165782 | Novel | AD/AR | Het | VUS | ? | Growth failure |
| *HOXA11* (NM_005523.6):c.679G>C; p.(Gly227Arg) | PM2, PP3 | 7:27224085 | Novel | AD | Het | VUS | ? | Thrombotic thrombopenic purpura |
| *IFNGR2* (NM_005534.4):c.529G>A; p.(Val177Ile) | PM2, BP4 | 21:34799307 | Novel | AR | Het | VUS | ? | Tuberculosis of bones of foot |
| *UFSP2* (NM_018359.5):c.1376A>C; p.(Asn459Thr) | PM2, PP3 | 4:186321580 | Novel | AD/AR | Hom | VUS | Mother & Father | Disproportionate short stature |
| *DNAH1* (NM_015512.5):c.3595G>A; p.(Asp1199Asn) | PM2 | 3:52388973 | Novel | AR | Het | VUS | ? | Chronic cough |
| *DNAH1* (NM_015512.5):c.12089+9C>Tp.? | PM2, BP4 | 3:52432187 | Novel | AR | Het | VUS | ? | Chronic cough |
| *KMT2A* (NM_001197104.2):c.172C>G; p.(Pro58Ala) | PM2, BP4 | 11:118307399 | Novel | AD | Het | VUS | ? | Growth hormone deficiency |
| *MAP2K2* (NM_030662.4):c.520A>G; p.(Ser174Gly) | PM2, PP3 | 19:4102382 | Novel | AD | Het | VUS | ? | Growth hormone deficiency |
| *PACS2* (NM_001100913.3):c.1269-4A>G; p.? | PM2, PP3 | 14:105848247 | Reported | AD | Het | VUS | ? | Epilepsy, Dyskinesia |
| *RELN* (NM_005045.4):c.6549A>C; p.(Leu2183Phe) | PM2, PP3 | 7:103183300 | Novel | AR/AD | Het | VUS | ? | Epilepsy, Dyskinesia |
| *SON* (NM_032195.3):c.5943_5963del; p.(Ser1992_Arg1998del) | PM2, PM4 | 21:34927468 | Novel | AD | Het | VUS | ? | Autism, speech delay |
| *JMJD1C* (NM_032776.3):c.803A>T; p.(Gln268Leu) | PM2, BP4 | 10:64975332 | Novel | AD | Het | VUS | ? | Autism, speech delay |
| *TNFRSF13B* (NM_012452.3):c.577T>C; p.(Cys193Arg) | PM2, BP4 | 17:16843694 | Reported | AD/AR | Het | VUS | ? | Perianal fistula |
| *RAC2* (NM_002872.5):c.328A>G; p.(Ile110Val) | PM2, BP4 | 22:37627391 | Novel | AD/AR | Het | VUS | Mother | Perianal fistula |
| *NLRP12* (NM_144687.4):c.2619T>G; p.(Cys873Trp) | PM2, BP4 | 19:54304618 | Novel | AD | Het | VUS | ? | Perianal fistula |
| *ATP1A3* (NM_152296.5):c.2418+3G>A; p.? | PM2, BP4 | 19:42474537 | Reported | AD | Het | VUS | ? | Infantile-onset seizures |
| *KCNB1* (NM_004975.4):c.1883G>A; p.(Gly628Glu) | BP4 | 20:47990214 | Novel | AD | Het | VUS | ? | Infantile-onset seizures |
| *SIK1* (NM_173354.5):c.2126T>C; p.(Leu709Pro) | PM2 | 21:44836848 | Novel | AD | Het | VUS | ? | Focal epilepsy |
| *CPT2* (NM_000098.3):c.578G>A; p.(Arg193His) | PM2, PM5, PP3 | 1:53675924 | Reported | AR | Het | VUS | Mother | Muscle pain |
| *RYR1* (NM_000540.3):c.2335G>A; p.(Val779Ile) | PM2, PP3 | 19:38949953 | Novel | AD/AR | Het | VUS | Father | Muscle pain |
| *PGM3* (NM_001199917.2):c.1178T>C; p.(Ile393Thr) | PM2, BP4 | 6:83884157 | Novel | AR | Het | VUS | Mother & Father | Immunodeficiency |
| *NFAT5* (NM_138714.4):c.2863A>G; p.(Met955Val) | PM2, BP4 | 16:69726591 | Novel | AD/AR | Het | VUS | ? | Inflammatory bowel disease |
| *STIL* (NM_003035.2):c.2452A>G; p.(Thr818Ala) | PM2, BP4 | 1:47735470 | Reported | AR | Het | VUS | ? | Dystonia |
| *MBD5* (NM_018328.5):c.2627G>A; p.(Gly876Glu) | PM2, PP3, BS2, PS2 | 2:149240787 | Reported | AD | Het | VUS | De novo | Refractory atypical absence seizure |
| *C3* (NM_000064.4):c.3478G>A; p.(Glu1160Lys) | PS3_Moderate, PM2, BP4 | 19:6690651 | Reported | AD/AR | Het | VUS | ? | Atypical hemolytic uremic syndrome |
| *FAT2* (NM_001447.3):c.2891T>C; p.(Leu964Pro) | PM2, PP3 | 5:1509456 | Novel | AD | Het | VUS | ? | Ataxia |
| *TMEM240* (NM_001114748.2):c.457G>A; p.(Gly153Arg) | PM2, PP3 | 1:1470804 | Novel | AD | Het | VUS | ? | Ataxia |
| *UNC13D* (NM_199242.3):c.1820G>A; p.(Arg607Gln) | PM2, PP3, PM5 | 17:73831518 | Novel | AR | Het | VUS | ? | Hypoalbuminemia |
| *SYNE1* (NM_033071.4):c.25480C>T; p.(Arg8494Trp) | PM2, PP3 | 6:152457788 | Reported | AD/AR | Het | VUS | ? | Global developmental delay |
| *RAI1* (NM_030665.4):c.2589G>C; p.(Glu863Asp) | PM2, BP4 | 17:17698851 | Novel | AD | Het | VUS | Father | Global developmental delay |
| *ATXN7* (NM_000333.4):c.2245dup; p.(Thr749Asnfs*7) | PVS1_Moderate, PM2 | 3:63981742 | Novel | AD | Het | VUS | ? | Spasticity |
| *CFTR* (NM_000492.4):c.640C>G; p.(Leu214Val) | PM2, PP3 | 7:117175362 | Reported | AR | Het | VUS | ? | Hydronephrosis |
| *GALE* (NM_000403.4):c.889G>A; p.(Val297Met) | PM2, BP4 | 1:24122740 | Novel | AR | Hom | VUS | ? | Abnormal findings on newborn screening |
| *PLCG2* (NM_002661.5):c.3393T>A; p.(Phe1131Leu) | PM2, PP3 | 16:81973576 | Reported | AD | Het | VUS | ? | Recurrent fever |
| *PRRT2* (NM_145239.3):c.891C>G; p.(Ser297Arg) | PM2, PM5, PP3 | 16:29825665 | Reported | AD | Het | VUS | ? | Seizure |
| *GRIN2A* (NM_000833.5):c.77C>T; p.(Ala26Val) | PM2, BP4 | 16:10274192 | Reported | AD | Het | VUS | ? | Seizure |
| *GRIN2B* (NM_000834.5):c.411+4A>C; p.? | PM2, PP3 | 12:14018728 | Novel | AD | Het | VUS | ? | Seizure |
| *GABRB2* (NM_021911.3):c.1334G>T; p.(Gly445Val) | PM2, PP3 | 5:160721293 | Novel | AD | Het | VUS | ? | Seizure |
|  |  |  |  |  |  |  |  |  |
| *SPTAN1* (NM_001130438.3):c.3358G>A; p.(Gly1120Arg) | PM2, PP3 | 9:131356596 | Reported | AD | Het | VUS | ? | Seizure |
| *CD2AP* (NM_012120.3):c.1583C>T; p.(Ala528Val) | PM2, BP4 | 6:47575715 | Novel | AD/AR | Het | VUS | ? | Nephrotic Syndrome |
| *PAX2* (NM_003987.5):c.*20A>T:p.? | PM2 | 10:102587396 | Novel | AD | Het | VUS | ? | Nephrotic Syndrome |
| *POLG* (NM_002693.3):c.3176A>G; p.(Asn1059Ser) | PM2, PP3 | 15:89862259 | Reported | AD/AR | Het | VUS | ? | Status epilepticus |
| *FOXP2* (NM_014491.4):c.1436A>T; p.(His479Leu) | PM2, PP3 | 7:114298290 | Novel | AD | Het | VUS | De novo | Seizure disorder |
| *MICU1* (NM_006077.4):c.330+3A>Gp.? | PM2, PP3 | 10:74322650 | Novel | AR | Hom | VUS | Mother & Father | Epilepsy |
| *KIF1C* (NM_006612.6):c.3081C>A; p.(His1027Gln) | PM2 | 17:4927215 | Reported | AR | Het | VUS | Mother | Epilepsy |
| *KIF1C* (NM_006612.6):c.3028A>G; p.(Thr1010Ala) | PM2, BP4 | 17:4927162 | Novel | AR | Het | VUS | Father | Epilepsy |
| *SLC12A2* (NM_001046.3):c.80_103del; p.(Leu27_Leu34del) | PM2, PM4 | 5:127419723 | Novel | AD/AR | Hom | VUS | Mother & Father | Kallmann’s syndrome |
| *DEPDC5* (NM_001242896.3):c.4583G>A; p.(Arg1528Gln) | PM2, PP3 | 22:32302254 | Reported | AD | Het | VUS | Mother | Epilepsy |
| *CACNA1D* (NM_000720.4):c.4852+3C>T; p.? | PM2, BP4 | 3:53815697 | Novel | AD/AR | Het | VUS | Mother | Epilepsy |
| *SPART* (NM_015087.5):c.1643G>A; p.(Gly548Glu) | PM2, PP3 | 13:36886372 | Novel | AR | Hom | VUS | Mother & Father | Intellectual disability |
| *TXNRD2* (NM_006440.5):c.528+2T>C; p.? | PVS1_Moderate, PM2 | 22:19903286 | Reported | AR | Hom | VUS | Mother & Father | Intellectual disability |
| *NNT* (NM_012343.4):c.188A>T; p.(Lys63Ile) | PM2, PP3 | 5:43613046 | Novel | AR | Het | VUS | ? | Congenital adrenal hypoplasia |
| *ATP1A2* (NM_000702.4):c.1577C>T; p.(Pro526Leu) | PM2, BP4 | 1:160100007 | Reported | AD/AR | Het | VUS | Father | Learning difficulty, History of epilepsy |
| *EPM2A* (NM_005670.4):c.148G>A; p.(Gly50Arg) | PM2, BP4 | 6:146056487 | Reported | AR | Hom | VUS | Mother & Father | Learning difficulty, History of epilepsy |
| *ZFYVE27* (NM_001002261.4):c.551+1G>A; p.? | PVS1_Moderate, PM2 | 10:99508122 | Novel | AD | Hom | VUS | Mother & Father | Learning difficulty, History of epilepsy |
| *COL6A3* (NM_004369.4):c.1897+3A>G; p.? | PM2, PP3 | 2:238289555 | Reported | AD/AR | Het | VUS | ? | Muscle weakness |
| *TTN* (NM_133378.4):c.25993C>A; p.(Gln8665Lys) | PM2, PP3 | 2:179569474 | Novel | AD/AR | Het | VUS | ? | Muscle weakness |
| *TRIO* (NM_007118.4):c.8798A>G; p.(Lys2933Arg) | PM2, PP3 | 5:14508035 | Novel | AD | Het | VUS | ? | Abnormal gait |
| *GFAP* (NM_002055.5):c.253G>A; p.(Glu85Lys) | PM2, PP3 | 17:42992602 | Novel | AD | Het | VUS | De novo | Behavioural change |
| *COL6A3* (NM_004369.4):c.7655T>A; p.Ile2552Asn | PM2, BP4 | 2:238253006 | Novel | AD/AR | Het | VUS | ? | Abnormal gait |
| *COL6A3* (NM_004369.4):c.8278C>G; p.Leu2760Val | PM2, BP4 | 2:23824928 | Novel | AD/AR | Het | VUS | ? | Abnormal gait |
| *MACF1* (NM_012090.5):c.9515A>C; p.(Tyr3172Ser) | PM2, PP3 | 1:39854215 | Novel | AD | Het | VUS | ? | Intellectual disability |
| *RELN* (NM_005045.4):c.673T>G; p.(Cys225Gly) | PM2, PP3 | 7:103368638 | Reported | AD/AR | Het | VUS | ? | Seizures |
| *SPTAN1* (NM_001130438.3):c.5725G>A; p.(Ala1909Thr) | PM2, PP3 | 9:131381274 | Novel | AD | Het | VUS | ? | Seizures |
| *F2* (NM_000506.5):c.650G>A; p.(Arg217Gln) | PM2 | 11:46747499 | Novel | AD/AR | Het | VUS | ? | Thrombophilia |

| **Table S11. CMA Variants of Uncertain Clinical Significance (VUS)** | | | |
| --- | --- | --- | --- |
| **Variants (cDNA; protein)** | **Size** | **Zygosity** | **ACMG-AMP CODES** |
| arr[GRCh37] 2p12(82049690_82408274)x4 | 359Kb | Hom Dup | 1B |
| arr[GRCh37] 6q27(167156393_167360766)x3 | 204kb | Het Dup | 1A, 3A |
| arr[GRCh37] 2q13(110498141_110983418)x3 | 485kb | Het Dup | 1A,3A |
| arr[GRCh37] 2q11.2(100408581_101224931)x3 | 816Kb | Het Dup | 1A, 3A |
| arr[GRCh37] 3q24(143485705_143617556)x3 | 132Kb | Het Dup | 1A,3A |
| arr[GRCh37] 6p12.1(53433566_53737669)x1 | 304Kb | Het Del | 1A,3A |
| arr[GRCh37] 20p12.3(8097724_8586513)x3 | 489Kb | Het Dup | 1A,3A |
| arr[GRCh37]12q21.31(86027767_86634908)x3 | 607Kb | Het Dup | 1A, 3A |
| arr[GRCh37] 16q23.1(77399877_77883983)x3 | 484Kb | Het Dup | 1A, 3A |
| arr[GRCh37] 2q13(110874326_111365996)x1 | 492kb | Het Del | 1A, 3A |
| arr[GRCh37] 2q33.1(198879699_199459477)x3 | 580kb | Het Dup | 1A,3A |
| arr[GRCh37] 20q12(39172727_40361264)x3 | 1.2Mb | Het Dup | 1A,3A |
| arr[GRCh37] 10p15.3p15.2(2691575_3134776)x3 | 443kb | Het Dup | 1A,3A |
| arr[GRCh37] 8p22(13283094_13566234)x3 | 283kb | Het Dup | 1A, 3A |
| arr[GRCh37] 12p13.33(657070_885168)x3 | 228kb | Het Dup | 1A,3A |
| arr[GRCh37]18q21.31(55345285_55708590)x3 | 363kb | Het Dup | 1A, 3A |
| arr[GRCh37] 19q11q12(28271146_28959499)x3 | 688kb | Het Dup | 1B |
| arr[GRCh37] 9p24.3(203861_353602)x3 | 150Kb | Het Dup | 1A, 3A |
| arr[GRCh37] 9q34.3(140018931_140418061)x1 | 399Kb | Het Del | 1A, 3A |
| arr[GRCh37] 18p11.31(3363780_3425856)x1 | 62Kb | Het Del | 1A,2B, 2C-2, 3A |
| arr[GRCh37] 6p22.3(16490847_16607397)x1 | 117Kb | Het Del | 1A, 3A |
| arr[GRCh37] 6q21(111698944_111882703)x3 | 184Kb | Het Dup | 1A,3A |
| arr[GRCh37] Xp22.11(23008799_23149191)x1 | 140Kb | Het Del | 1A,3A |
| Arr[GRCh37]Yq11.223q11.23(24651462_28458663)x2 | 3.8Mb | Het Dup | 1A,3A |
| arr[GRCh37] 20p12.1(14702914_14833222)x1 | 130Kb | Het Del | 1A, 3A |
| arr[GRCh37] 6q13(74803480_75459023)x4 | 655Kb | Het Dup | 1B |
| arr[GRCh37] 10p11.21(34907533_35182968)x3 | 275Kb | Het Dup | 1A 3A |
| arr[GRCh37] 16p13.11(16221990_16309165)x3 | 87Kb | Het Dup | 1A 3A |
| arr[GRCh37] Xq21.31(87990552_88259035)x1 | 268Kb | Het Del | 1A,3A |
| arr[GRCh37] 3q26.32(176822675_177088332)x3 | 266Kb | Het Dup | 1A,2J/2K,3A |
| arr[GRCh37] 18p11.32(288322_642932)x3 | 355Kb | Het Dup | 1A,3A |
| arr[GRCh37] 18p11.32(815828_1197643)x3 | 382Kb | Het Dup | 1A,3A |
| arr[GRCh37] 22q11.23q12.1(25536777_26087539)x3 | 551Kb | Het Dup | 1A,3A |
| arr[GRCh37] 20p12.3(7106058_8586513)x3 | 1.5Mb | Het Dup | 1A,3A |
| arr[GRCh37] 10q21.3(68339607_68394905)x0 | 55Kb | Homo Del | 1A,3A |
| arr[GRCh37] 7q36.1(151938223_152252996)x3 | 315Kb | Het Dup | 1A,2J,2K,3A |
| arr[GRCh37] 11q14.1(81391435_81909193)x1 | 518Kb | Het Del | 1B |
| arr[GRCh37] Xq21.31(87990553_88259035)x1 | 268Kb | Het Del | 1A,3A |
| arr[GRCh37] 7p21.2(14984747_15070182)x1 | 85Kb | Het Del | 1A,3A |
| arr[GRCh37] 13q22.1q22.2(75163345_76383373)x3 | 1.2Mb | Het Dup | 1A,3A |
| arr[GRCh37] 7p11.2(54036107_54619356)x3 | 583.25 kb | Het Dup | 1A,3A |
| arr[GRCh37] 7p12.3(45446041_45783375)x3 | 337Kb | Het Dup | 1A,3A |
| arr[GRCh37] 20q13.2(54759454_54920341)x1 | 160 kb | Het Del | 1A,3A |
| arr[GRCh37] 9p24.3(203861_357994)x3 | 154kb | Het Dup | 1A,3A |
| arr[GRCh37] 2p16.3(50885714_50946373)x1 | 61Kb | Het Del | 1A,2E,3A |
| arr[GRCh37] 6q26(162793214_163053738)x3 | 260 Kb | Het Dup | 1A,3A |
| arr[GRCh37] 10q26.11(120642944_121065905)x3 | 422kb | Het Dup | 1A,3A |
| arr[GRCh37] 12q14.1(60406405_61095046)x1 | 689Kb | Het Del | 1B |
| arr[GRCh37] 16p13.3(6547044_6844992)x1 | 298kb | Het Del | 1A,3A |
| arr[GRCh37] 3p26.3(61892_399110)x3 | 337Kb | Het Dup | 1A,3A |
| arr[GRCh37] 11q14.1(78230643_78366709)x3 | 136kb | Het Dup | 1A,3A |
| arr[GRCh37] Xq27.3(143732141_144487222)x3 | 755Kb | Het Dup | 1A,3A |
| arr[GRCh37] 9p24.3(203862_388406)x3 | 185Kb | Het Dup | 1A,3A |
| arr[GRCh37] Xq27.2(140673423_140783042)x0 | 110Kb | Hom Del | 1A,3A |
| arr[GRCh37] 7q31.1(110964518_111296229)x1 | 332Kb | Het Del | 1A,3A |
| arr[GRCh37] 5p13.1(38408950_38483082)x1 | 74 kb | Het Del | 1A,3A |
| arr[GRCh37] 18p11.31(4222207_4429639)x1 | 207Kb | Het Del | 1A,3A |
| arr[GRCh37] 10q21.3(68215242_68347537)x1 | 132.2 kb | Het Del | 1A,3A |
| arr[GRCh37] 10p15.3p15.2(2689086_3134776)x3 | 446Kb | Het Dup | 1A,3A |
| arr[GRCh37] 4q34.3(179501536_182588424)x3 | 3.1Mb | Het Dup | 1B |
| arr[GRCh37] 5p15.2(13790105_14186638)x3 | 397Kb | Het Dup | 1A,2J,2K,3A |
| arr[GRCh37] 7q33(133272218_133379895)x1 | 108Kb | Het Del | 1A,3A |
| arr[GRCh37] 5q35.3(178712059_178936878)x3 | 225Kb | Het Dup | 1A,3A |
| arr[GRCh37] 12q24.32(128001659_128760368)x3 | 759Kb | Het Dup | 1B |
| arr[GRCh37] 2q22.1(138077613_138418875)x1 | 341Kb | Het Del | 1A,3A |
| arr[GRCh37] 5p15.2(13799724_14180307)x3 | 381Kb | Het Dup | 1A,2J, 2K,3A |
| arr[GRCh37] 15q13.1(28325566_28522838)x1 | 197Kb | Het Del | 1A,2B,3A |
| arr[GRCh37] 4q21.1(77119485_77216105)x3 | 97Kb | Het Dup | 1A,3A |
| arr[GRCh37] 4q31.1(140050388_140169116)x3 | 119Kb | Het Dup | 1A,3A |
| arr[GRCh37] 16q23.1(78219339_78280148)x1 | 61Kb | Het Del | 1A,3A |
| arr[GRCh37] 10q21.3(68268281_68495602)x1 | 227kb | Het Del | 1A,3A |
| arr[GRCh37] 15q26.3(100167696_100632500)x3 | 465Kb | Het Dup | 1A,3A |
| arr[GRCh37] Xp21.1(32004087_32097831)x1 | 94Kb | Het Del | 1A,2E,3A |
| arr[GRCh37] 16q23.1(77672183_78022343)x1 | 350Kb | Het Del | 1A,3A |
| arr[GRCh37] 2q36.3(226413083_226783247)x3 | 370 kb | Het Dup | 1A,3A |
| arr[GRCh37] 1q21.1(145625129_145888926)x1 | 264kb | Het Del | 1A,3A |
| arr[GRCh37] 2q14.1(116033580_116355897)x1 | 322kb | Het Del | 1A,3A |
| arr[GRCh37] 18p11.32(826,085_1,197,643)x3 | 372Kb | Het Dup | 1A,3A |
| arr[GRCh37] 18p11.32(290,284_642,932)x3 | 353Kb | Het Dup | 1A,3A |
| arr[GRCh37] Yp11.2(3,328,726_6,101,384)x2 | 2.8Mb | Het Dup | 1A,3A |
| arr[GRCh37] 15q11.2(23867593_24098328)x3 | 231kb | Het Dup | 1A,2B,3A |
| arr[GRCh37] 6q14.3(84951055_85471464)x3 | 520kb | Het Dup | 1A,3A |
| arr[GRCh37] 1q41(220027846_220122951)x3 | 95Kb | Het Dup | 1B |
| arr[GRCh37] Xp22.33(168,547_695,082)x3 | 527kb | Het Dup | 1A,3A |
| arr[GRCh37] 10q22.3(81,297,994_81,437,912)x1 | 140kb | Het Del | 1A,3A |
| arr[GRCh37] 10q22.3(81,597,767_81,971,690)x3 | 374kb | Het Dup | 1A,3A |
| arr[GRCh37] 8q24.23(138,093,449_139,888,133)x3 | 1.8Mb | Het Dup | 1A,3A |
| arr[GRCh37] 1q43(237,324,788_237,787,527)x1 | 463kb | Het Del | 1A,3A |
| arr[GRCh37] 18p11.32(2,275,729_2,710,888)x3 | 435Kb | Het Dup | 1A,3A |
| arr[GRCh37] 2q31.1(174,416,135_175,037,435)x3 | 621Kb | Het Dup | 1A,3A |
| arr[GRCh37] 20p12.2(9,726,101_10,534,312)x3 | 808Kb | Het Dup | 1A,3A |
| arr[GRCh37] 5p14.3(19,454,271_19,575,593)x1 | 121kb | Het Del | 1A,3A |
| arr[GRCh37] 1q31.3(194,699,165_195,489,907)x1 | 791kb | Het Del | 1B |
| arr[GRCh37] 9q21.2(80488632_80877566)x3 | 389Kb | Het Dup | 1A,3A |
| arr[GRCh37] 8q23.3(113,678,568_114,296,483)x1 | 618Kb | Het Del | 1A,3A |
| arr[GRCh37] 5q31.2(136,305,333_136,765,053)x3 | 460Kb | Het Dup | 1A,3A |
| arr[GRCh37] 15q11.2(22,770,422_23,082,328)x3 | 312Kb | Het Dup | 1A,2B,3A |
| arr[GRCh37] 7p12.3(47,843,166_48,375,867)x3 | 533Kb | Het Dup | 1A,3A |
| arr[GRCh37] 17p13.3(1,540,106_2,494,662)x1 | 955Kb | Het Del | 1A,2H,3A |
| arr[GRCh37] 8p23.2(3,673,769_3,816,081)x1 | 142Kb | Het Del | 1A,3A |
| arr[GRCh37] Xp22.33 or Yp11.32(1,752,332_2,387,869 or 1,702,332_2,337,869)x3 | 636Kb | Het Dup | 1A,3A |
| arr[GRCh37] 15q13.3q14(33,197,987_33,676,561)x3 | 479Kb | Het Dup | 1A,3A |
| arr[GRCh37] 15q14(36,664,636_36,924,785)x3 | 260Kb | Het Dup | 1A,3A |
| arr[GRCh37] 10q24.2(100,907,945_101,178,330)x3 | 270Kb | Het Dup | 1A,3A |
| arr[GRCh37] 10q21.3(68,339,607_68,405,697)x1 | 66Kb | Het Del | 1A,3A |
| arr[GRCh37] 1p22.1(92,188,177_92,573,770)x3 | 386kb | Het Dup | 1A,3A |
| arr[GRCh37] 7p11.2(54,036,107_54,611,414)x3 | 575Kb | Het Dup | 1A,3A |
| arr[GRCh37] 9p23p22.3(14,098,731_14,294,068)x3 | 195Kb | Het Dup | 1A,3A |
| arr[GRCh37] 15q11.2(24,025,136_24,144,958)x3 | 120Kb | Het Dup | 1B,2B |
| arr[GRCh37] 2q36.3(230,538,161_230,683,400)x3 | 145kb | Het Dup | 1A,2J,2K,3A |
| arr[GRCh37] 6q13(74,803,481_75,459,023)x3 | 656kb | Het Dup | 1B |
| arr[GRCh37] 17q25.3(80,226,654_80,373,487)x3 | 147Kb | Het Dup | 1A,3A |
| arr[GRCh37] Xp22.31(7,524,470_8,131,014)x4 | 607Kb | Hom Dup | 1A,2J,2K,3A |
| arr[GRCh37] 4p15.32p15.31(16,826,922_17,825,983)x3 | 999Kb | Het Dup | 1A,3A |
| arr[GRCh37] 8q24.3(145,738,056_145,960,474)x3 | 222Kb | Het Dup | 1A,3A |
| arr[GRCh37] 6q23.3(136,850,948_136,979,240)x 3 | 128Kb | Het Dup | 1A,3A |
