# Supplementary figures and images for: The genomic landscape of rare disorders in the Middle East
Source: Genome Med. 2023 Jan 27;15:5. doi: 10.1186/s13073-023-01157-8 (PMC9881316; doi:10.1186/s13073-023-01157-8)

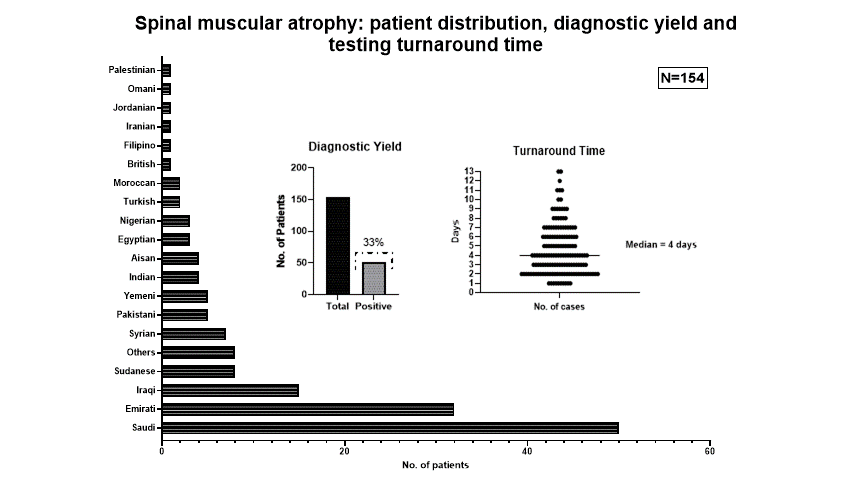

Supplement: Supplementary file 2 — Additional file 2: Figure S1. SpinalMuscular Atrophy (SMA) cohort (N = 138). A) Distribution of patient origins bycountry. Patients represent 19 countries; majority are Arabs mostly from SaudiArabi, United Arab Emirates, Iraq, Sudan, and Syria. B) Testing was positive in48 cases for a diagnostic yield of ~35%. C) Average Turnaround time was 4 days, with majority (61%) receiving results in ≤ 4days. [file 13073_2023_1157_MOESM2_ESM.gif]

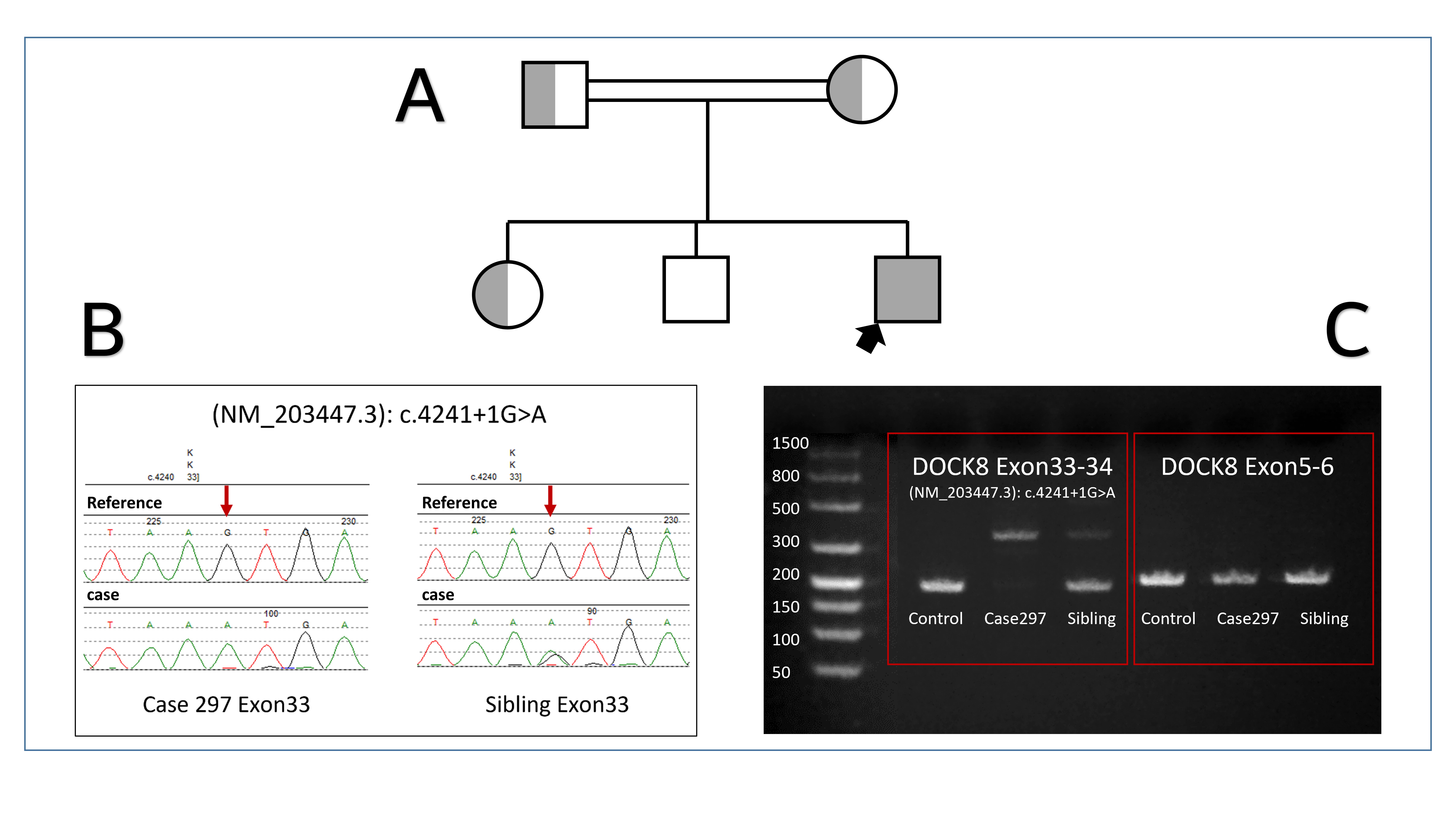

Supplement: Supplementary file 3 — Additional file 3: Figure S2. Genomicand RNA analysis of DOCK8 in case 297. A) Family pedigree. Filledsquare denotes affected proband (arrow); half filed squares/circles denote confirmed (female sibling) or obligate (parents) heterozygous carriers. Parentswere first cousins. B) PCR amplification and Sanger sequencing ofgenomic DNA, using primers targeting exon 33 and splice regions (table below),confirmed the DOCK8 (NM_203447.3):c.4241+1G>A variant in thehomozygous state in case 297 and heterozygous state in the female sibling. C)cDNA from patient, his female sibling, and a control sample was synthesized andimpact of the DOCK8 (NM_203447.3):c.4241+1G>A variant on splicing was assessed using PCR primers targeting exons 33 and 34 (table below). A normalPCR product with an expected size of 169bp was observed in thecontrol sample suggesting normal splicing between exons 33 and 34. The normalcDNA 169bp product was not detected in the patient suggesting abnormal splicingdue to the c.4241+1G>A variant. Instead, a cryptic cDNA product >300bp insize is identified in the patient. Female sibling carried both the normal(169bp) and cryptic (>300bp) products, a finding which is consistent withher heterozygous carrier status (panel B). A control PCR product (186bp) wasamplified from cDNA of all samples using primers targeting exons 5 and 6. Allprimer sequences are provided below. [file 13073_2023_1157_MOESM3_ESM.jpg]
